# Profiling RNA-Seq at multiple resolutions markedly increases the number of causal eQTLs in autoimmune disease

**DOI:** 10.1371/journal.pgen.1007071

**Published:** 2017-10-23

**Authors:** Christopher A. Odhams, Deborah S. Cunninghame Graham, Timothy J. Vyse

**Affiliations:** 1 Department of Medical & Molecular Genetics, King’s College London, London, United Kingdom; 2 Academic Department of Rheumatology, Division of Immunology, Infection and Inflammatory Disease, King’s College London, London, United Kingdom; New York Genome Center & Columbia University, UNITED STATES

## Abstract

Genome-wide association studies have identified hundreds of risk loci for autoimmune disease, yet only a minority (~25%) share genetic effects with changes to gene expression (eQTLs) in immune cells. RNA-Seq based quantification at whole-gene resolution, where abundance is estimated by culminating expression of all transcripts or exons of the same gene, is likely to account for this observed lack of colocalisation as subtle isoform switches and expression variation in independent exons can be concealed. We performed integrative *cis*-eQTL analysis using association statistics from twenty autoimmune diseases (560 independent loci) and RNA-Seq data from 373 individuals of the Geuvadis cohort profiled at gene-, isoform-, exon-, junction-, and intron-level resolution in lymphoblastoid cell lines. After stringently testing for a shared causal variant using both the Joint Likelihood Mapping and Regulatory Trait Concordance frameworks, we found that gene-level quantification significantly underestimated the number of causal *cis*-eQTLs. Only 5.0–5.3% of loci were found to share a causal *cis*-eQTL at gene-level compared to 12.9–18.4% at exon-level and 9.6–10.5% at junction-level. More than a fifth of autoimmune loci shared an underlying causal variant in a single cell type by combining all five quantification types; a marked increase over current estimates of steady-state causal *cis*-eQTLs. Causal *cis*-eQTLs detected at different quantification types localised to discrete epigenetic annotations. We applied a linear mixed-effects model to distinguish *cis*-eQTLs modulating all expression elements of a gene from those where the signal is only evident in a subset of elements. Exon-level analysis detected disease-associated *cis*-eQTLs that subtly altered transcription globally across the target gene. We dissected in detail the genetic associations of systemic lupus erythematosus and functionally annotated the candidate genes. Many of the known and novel genes were concealed at gene-level (e.g. *IKZF2*, *TYK2*, *LYST*). Our findings are provided as a web resource.

## Author summary

It is well acknowledged that non-coding genetic variants contribute to disease susceptibility through alteration of gene expression levels (known as eQTLs). Identifying the variants that are causal to both disease risk and changes to expression levels has not been easy and we believe this is in part due to how expression is quantified using RNA-Sequencing (RNA-Seq). Whole-gene expression, where abundance is estimated by culminating expression of all transcripts or exons of the same gene, is conventionally used in eQTL analysis. This low resolution may conceal subtle isoform switches and expression variation in independent exons. Using isoform-, exon-, and junction-level quantification can not only point to the candidate genes involved, but also the specific transcripts implicated. We make use of existing RNA-Seq expression data profiled at gene-, isoform-, exon-, junction-, and intron-level, and perform eQTL analysis using association data from twenty autoimmune diseases. We find exon-, and junction-level thoroughly outperform gene-level analysis, and by leveraging all five quantification types, we find >20% of autoimmune loci share a single genetic effect with gene expression. We highlight that existing and new eQTL cohorts using RNA-Seq should profile expression at multiple resolutions to maximise the ability to detect causal eQTLs and candidate genes.

## Introduction

The autoimmune diseases are a family of heritable, often debilitating, complex disorders in which immune dysfunction leads to loss of tolerance to self-antigens and chronic inflammation [1]. Genome-wide association studies (GWAS) have now detected hundreds of susceptibility loci contributing to risk of autoimmunity [2] yet their biological interpretation still remains challenging [3]. Mapping single nucleotide polymorphisms (SNPs) that influence gene expression (eQTLs) can provide meaningful insight into the potential candidate genes and etiological pathways connected to discrete disease phenotypes [4]. For example, such analyses have implicated dysregulation of autophagy in Crohn’s disease [5], the pathogenic role of CD4^+^ effector memory T-cells in rheumatoid arthritis [6], and an overrepresentation of transcription factors in systemic lupus erythematosus [7].

Expression profiling in appropriate cell types and physiological conditions is necessary to capture the pathologically relevant regulatory changes driving disease risk [8]. Lack of such expression data is thought to explain the observed disparity of shared genetic architecture between disease association and gene expression at certain autoimmune loci [9]. A much overlooked cause of this disconnect however, is not only the use of microarrays to profile gene expression, but also the resolution to which expression is quantified using RNA-Sequencing (RNA-Seq) [10]. Expression estimates of whole-genes, individual isoforms and exons, splice-junctions, and introns are obtainable with RNA-Seq [11–18]. The SNPs that affect these discrete units of expression vary strikingly in their proximity to the target gene, localisation to specific epigenetic marks, and effect on translated isoforms [18]. For example, in over 57% of genes with both an eQTL influencing overall gene expression and a transcript ratio QTL (trQTL) affecting the ratio of each transcript to the gene total, the causal variants for each effect are independent and reside in distinct regulatory elements of the genome [18].

RNA-Seq based eQTL investigations that solely rely on whole-gene expression estimates are likely to mask the allelic effects on independent exons and alternatively-spliced isoforms [16–19]. This is in part due to subtle isoform switches and expression variation in exons that cannot be captured at gene-level [20]. A large proportion of trait associated variants are thought to act via direct effects on pre-mRNA splicing that do not change total mRNA levels [21]. Recent evidence also suggests that exon-level based strategies are more sensitive than conventional gene-level approaches, and allow for detection of moderate but systematic changes in gene expression that are not necessarily derived from alternative-splicing events [15,22]. Furthermore, gene-level summary counts can be biased in the direction of extreme exon outliers [22]. Use of isoform-, exon-, and junction-level quantification in eQTL analysis also support the potential to not only point to the candidate genes involved, but also the specific transcripts or functional domains affected [10,18]. This of course facilitates the design of targeted functional studies and better illuminates the causative relationship between regulatory genetic variation and disease. Lastly, though intron-level quantification is not often used in conventional eQTL analysis, it can still provide valuable insight into the role of unannotated exons in reference gene annotations, retained introns, and even intronic enhancers [23,24].

Low-resolution expression profiling with RNA-Seq will impede the subsequent identification of causal eQTLs when applying genetic and epigenetic fine-mapping approaches [25]. In this investigation, we aim to increase our knowledge of the regulatory mechanisms and candidate genes of human autoimmune disease through integration of GWAS and RNA-Seq expression data profiled at gene-, isoform-, exon-, junction-, and intron-level in lymphoblastoid cell lines (LCLs). This is firstly performed in detail using association data from a GWAS in systemic lupus erythematosus, and is then scaled up to a total of twenty autoimmune diseases. Our findings are provided as a web resource to interrogate the functional effects of autoimmune associated SNPs (www.insidegen.com), and will serve as the basis for targeted follow-up investigations.

## Results

### Gene-level expression quantification underestimates the number of causal cis-eQTLs

Using densely imputed genetic association data from a large European GWAS in systemic lupus erythematosus (SLE) [7], we performed integrative *cis*-eQTL analysis with RNA-Seq expression data profiled at five resolutions: gene-, transcript-, exon-, junction-, and intron-level. Expression data were derived from 373 healthy European donors of the Geuvadis project profiled in lymphoblastoid cell lines (LCLs) [18]. See S1 Fig for a summary of how expression at the five resolutions was quantified. A total of 38 genome-wide significant SLE loci (S1 Table) were put forward for analysis. To test for evidence of a single shared causal variant between disease and gene expression at each locus, we employed the Joint Likelihood Mapping (JLIM) framework [9] using summary-level statistics for SLE association and full genotype-level data for gene expression. Using JLIM, *cis*-eQTLs were defined if a nominal association (*P*<0.01) with at least one SNP existed within 100kb of the SNP most associated with disease and the transcription start site of the gene was located within +/-500kb of that SNP (as defined by authors of JLIM). JLIM *P*-values were corrected for multiple testing by a false discovery rate (FDR) of 5% per RNA-Seq quantification type (i.e. at exon-level, JLIM *P*-values were adjusted for total number of exons tested in *cis* to the 38 SNPs). Causal associations of the integrative *cis*-eQTL SLE GWAS analysis across the five RNA-Seq quantification types are available in S2 Table and the full output (including non-causal associations) are available in S3 Table. The distribution of JLIM *P*-values across the five RNA-Seq quantification types are depicted in S2 Fig.

We found the number of causal *cis*-eQTLs was markedly underrepresented when considering conventional gene-level quantification (Table 1). Only two of the 38 SLE susceptibility loci (5.3%) were deemed to be causal *cis*-eQTLs at gene-level for three candidate genes. This is a similar proportion observed by *Chun et al* [9] who found that 16 of the 272 (5.9%) autoimmune susceptibility loci tested were *cis*-eQTLs driven by a shared causal variant in the Geuvadis RNA-Seq dataset using gene-level quantification (based upon the seven autoimmune diseases interrogated—not including SLE).

**Table 1:** Number of cis-eQTLs driven by the same causal variant as the SLE disease association (total number of SLE loci: 38).

	Gene	Transcript	Exon	Junction	Intron	Total
Causal *cis*-eQTLs^*a*^	2	2	7	4	4	9^*b*^
% of 38 SLE GWAS loci	5.3	5.3	18.4	10.5	10.5	23.7
% of total causal eQTLs	22.2	22.2	77.8	44.4	44.4	100
Candidate genes	3	4	9	5	5	12
Expression targets^*c*^	2	7	24	18	13	64

The lead SNPs from the *Bentham and Morris et al 2015* GWAS in persons of European descent were functionally annotated by *cis*-eQTL analysis in the Geuvadis RNA-Seq cohort in lymphoblastoid cell lines using RNA-Seq quantification profiled at five resolutions (gene, transcript, exon, junction, and intron). Only SNPs reaching genome-wide significance, not conditional peaks, outside of the major histocompatibility complex loci, and with minor allele frequency > 5% were included leaving 38 SLE lead SNPs in total. All SLE loci were densely imputed to the 1000 Genomes Phase 3 Imputation Panel as described in methods.

All 38 loci (+/-100kb of each lead SNP) comprised a nominally significant *cis*-eQTL (*P*<0.01) for at least one gene within +/-500kb of the lead SNP at each resolution of RNA-Seq. Evidence of a single shared causal variant at each locus was assessed using the Joint Likelihood Mapping (JLIM) algorithm as described in methods.

^*a*^Number of loci where the disease association is consistent with a single shared effect for at least one *cis*-eQTL (*P*<0.01 and JLIM FDR adjusted *P*<0.05).

^*b*^The total number of unique causal *cis*-eQTLs across all RNA-Seq quantification types.

^*c*^Expression targets corresponds to the quantification type in hand (i.e. number of exons at exon-level).

Of note, transcript-level quantification did not increase the number of causal *cis*-eQTLs (Table 1). Transcript-level analysis did, however, yield a greater number of candidate genes (seven individual transcripts derived from a total of four genes). Both junction- and intron-level quantification increased the number of causal *cis*-eQTLs to four (10.5% of the 38 total SLE loci). Using exon-level quantification, we were able to detect seven significant *cis*-eQTLs driven by a single shared causal variant (18.4%). Exon-level analysis also produced the greatest number of candidate gene targets: nine unique genes derived from 24 individual SNP-exon pairs (Table 1). Therefore, even with the severe multiple testing burden, we firstly conclude that exon-, junction-, and intron-level analysis detects more causal *cis*-eQTLs than gene-level.

### A fifth of associated SNPs possess shared genetic effects with cis-eQTLs using RNA-Seq in LCLs

By combining all five types of RNA-Seq quantification (gene, transcript, exon, junction, and intron) we classified nine of the 38 SLE susceptibility loci (24%) as being driven by the same causal variant as the *cis*-eQTL in LCLs (Table 1). This value, derived from interrogating only a single cell type, is almost equal to the total number of causal autoimmune *cis*-eQTLs detected by *Chun et al* [9] (~25%) across three different cell types (CD4^+^ T-cells–measured by microarray, CD14^+^ monocytes–microarray, and LCLs–RNA-Seq gene-level).

We found that when considering the specificity of *cis*-eQTLs and target genes across the five RNA-Seq quantification types, both gene- and transcript-level quantification were redundant with respect to exon-level data; i.e. there were no causal *cis*-eQTLs or target genes detected at gene- or transcript-level that were not captured by exon-level analysis (S3 Fig). Both junction- and intron-level quantification captured a single causal *cis*-eQTL each that was not captured by exon-level. We conclude that profiling at all resolutions of RNA-Seq is required to capture the full set of potentially causal *cis*-eQTLs.

### Associated SNPs are most likely to colocalize with exon- and junction-level cis-eQTLs

**Fig 1:**
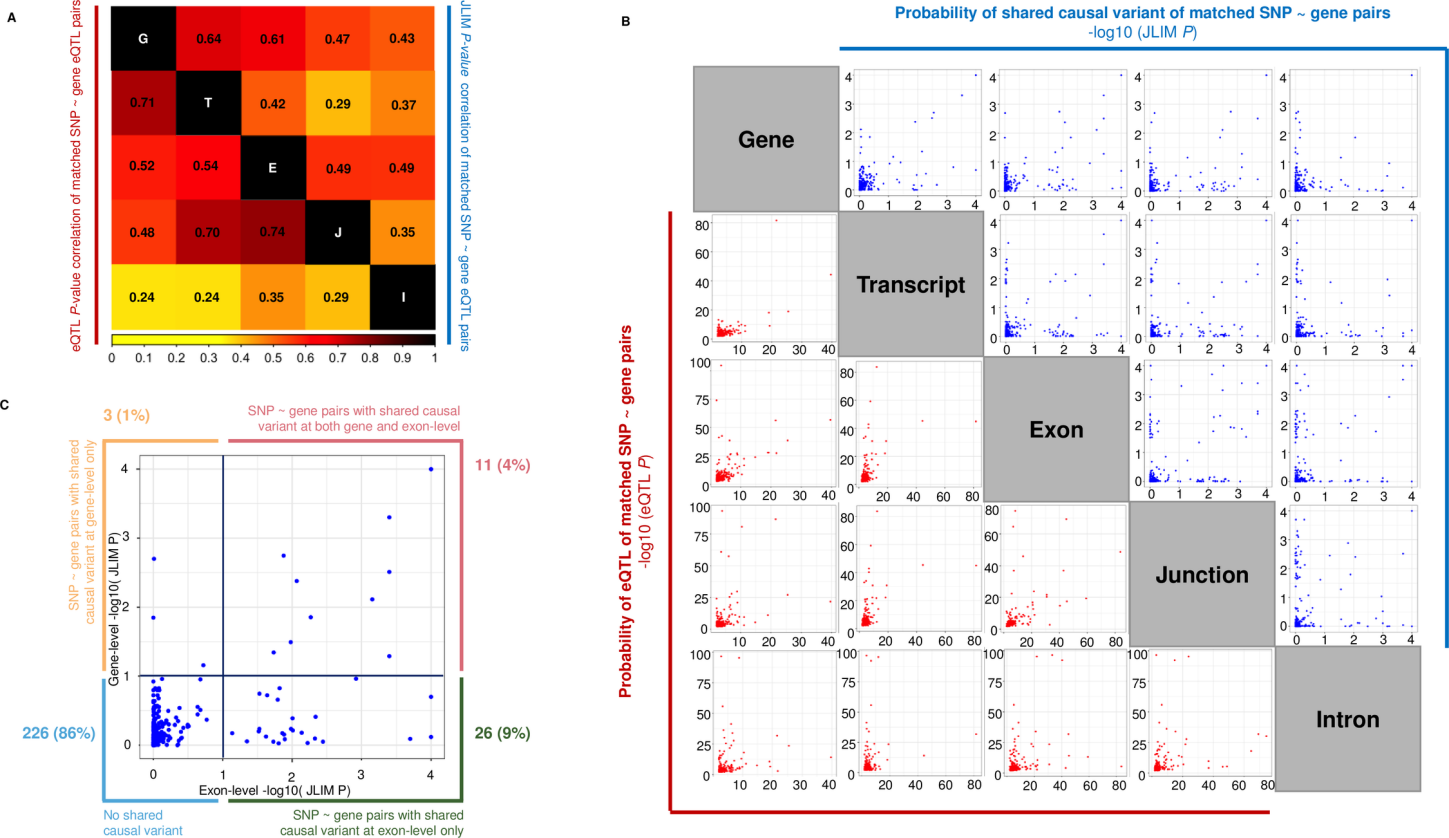
Pairwise comparison of cis-eQTL and JLIM P-values for matched SNP-gene pairs. This figure is complementary to the data in Table 2 and is derived from *cis*-eQTL analysis of the 38 SLE associated SNPs using RNA-Seq and implementation of the JLIM method to assess evidence of a shared causal variant. (A) We measured the Pearson’s correlation separately of all *cis*-eQTL and JLIM *P*-values between matched SNP-gene *cis*-eQTL pairs across the five RNA-Seq quantification types. We only considered matched SNP-gene *cis*-eQTL association pairs that had a nominal *cis*-eQTL association *P*-value < 0.01 in both quantification types, and to be conservative, when multiple transcripts, exons, junctions, and introns were annotated with the same gene symbol, we selected the associations that minimized the difference in JLIM *P*-value between matched SNP-gene *cis*-eQTLs across RNA-Seq quantification types. Note the weak JLIM *P*-value correlation of matched transcript-level and junction-level *cis*-eQTLs suggesting they stem from independent causal variants. (B) Correlation plots of matches SNP-gene *cis*-eQTL pairs as described above (red: *cis*-eQTL *P*-value; blue: JLIM *P*-value). Note that JLIM *P*-values often aggregate on the axis rather than on the diagonal suggesting independent causal variants across different quantification types. (C) An example of the sensitivity of exon-level analysis relative to gene-level. The majority of nominally significant JLIM *P*-values (<0.01) for matched SNP-gene pairs are captured by exon-level analysis and concealed at gene-level (green box: 9%).

We compared the detection of *cis*-eQTLs using a pairwise comparison between the five RNA-Seq quantification types for matched SNP-gene *cis*-eQTL pairs (Fig 1). We only considered matched SNP-gene *cis*-eQTL association pairs that had a nominal *cis*-eQTL association *P*-value < 0.01 in both quantification types, and to be conservative, when multiple transcripts, exons, junctions, and introns were annotated with the same gene symbol, we selected the associations that minimized the difference in JLIM *P*-value between matched SNP-gene *cis*-eQTLs across RNA-Seq quantification types. There were over 250 matched SNP-gene *cis*-eQTL pairs per comparison. We firstly observed that the correlation of both *cis*-eQTL association *P*-values from regression and JLIM *P*-values across RNA-Seq quantification types reflected the methods in which expression quantification was obtained (Fig 1A). Both *cis*-eQTL and JLIM *P*-values between matched SNP-gene pairs at gene- and transcript-level were highly correlated as gene-level estimates are obtained from the sum of all transcript-level estimates for the same gene. Exon-level and junction-level associations were also highly correlated due to split-reads being incorporated into the exon-level estimate. As expected, intron-level *cis*-eQTL and JLIM *P*-values for matched SNP-gene pairs were only weakly correlated against other quantification types—as reads mapping to introns are not included in the other quantification models. Interestingly, although *cis*-eQTL association *P*-values for matched SNP-gene pairs between transcript-level and junction-level were found to be relatively high (*r*^2^ = 0.70), we found the JLIM *P*-values for the matched pairs to be comparatively low (*r*^2^ = 0.29); suggesting that whilst the statistical significance of matched *cis*-eQTLs maybe similar between these quantification types, the underlying causal variants driving the disease and *cis*-eQTL association are likely to be independent.

By plotting the JLIM *P*-values for matched SNP-gene pairs between different quantification types, we found many instances of *P*-values distributed along the axes rather than on the diagonal (Fig 1B). Our findings therefore suggest that often, one quantification type is more likely to explain the observed disease association than the other. When we compared conventional gene-level *cis*-eQTL analysis against exon-level results (Fig 1C), we found that of the 296 matched SNP-gene *cis*-eQTL associations (*P*<0.01), eleven (4%) shared the same causal variant at both gene- and exon-level using a nominal JLIM *P*-value threshold <0.01. Only three of the 296 matched SNP-gene *cis*-eQTL associations (1%) were captured by gene-level only—in contrast to the 26 (9% of total associations) captured uniquely at exon-level. As expected, the overwhelming majority of *cis*-eQTL associations (86%) did not possess a single shared causal variant at either gene- or exon-level. We performed this analysis for all possible combinations of quantification types (Table 2). In each instance, gene-level analysis detected only the minority of nominally causal associations for matched SNP-gene association pairs (JLIM *P*<0.01). Exon-level and junction-level analysis consistently detected more causal *cis*-eQTL associations than gene-, transcript-, and intron-level. In fact, when combined, exon- and junction-level analysis explained the most nominally causal associations for all significant SNP-gene *cis*-eQTL association pairs (24%).

**Table 2:** Pairwise comparison of the number of cis-eQTLs with a nominal JLIM *P*-value < 0.01.

Quantification type X	Quantification type Y	Total matched *cis*-eQTLs (SNP ~ gene pairs *P* < 0.01)	% Shared causal variant in X and Y (JLIM *P* < 0.01)	% Shared causal variant in X only (JLIM *P* < 0.01)	% Shared causal variant in Y only (JLIM *P* < 0.01)	% No shared causal variant in X and Y (JLIM *P* < 0.01)	Correlation of JLIM *P* (X ~ Y)
Gene	Transcript	267	3.00	1.87	5.62	89.51	0.63
Gene	Exon	296	3.72	1.01	8.78	86.49	0.57
Gene	Junction	229	3.49	1.75	11.79	82.97	0.46
Gene	Intron	252	1.59	3.57	5.56	89.29	0.35
Transcript	Exon	325	3.08	5.54	9.54	81.85	0.38
Transcript	Junction	261	3.07	5.75	12.64	78.54	0.29
Transcript	Intron	279	2.15	6.45	5.73	85.66	0.24
Exon	Junction	294	6.12	7.82	9.86	76.19	0.44
Exon	Intron	314	2.87	10.83	4.78	81.53	0.34
Junction	Intron	275	3.27	13.45	5.09	78.18	0.20

This table is complementary to the data in Fig 1. We only considered matched SNP-gene *cis*-eQTL association pairs that had a nominal *cis*-eQTL association *P*-value < 0.01 in both quantification types, and to be conservative, when multiple transcripts, exons, junctions, and introns were annotated with the same gene symbol, we selected the associations that minimized the difference in JLIM *P*-value between matched SNP-gene *cis*-eQTLs across RNA-Seq quantification types. The first row for example is a pairwise comparison of matched SNP-gene pairs between gene-level and transcript-level quantification (of which there are 267 matched pairs). 3% of these are deemed nominally causal (JLIM *P* < 0.01) at both gene-level and transcript, 1.87% at gene-level only and 5.62% at transcript-level only. 89.51% of matched SNP-gene pairs between gene- and transcript-level do not possess a nominally causal *cis*-eQTL. Pearson’s correlation was performed for matched SNP-gene JLIM *P*-value pairs. These data show that exon- and junction-level analysis consistently capture the majority of potentially causal cis-eQTL associations. JLIM: joint likelihood mapping.

### Leveraging RNA-Seq aids GWAS interpretation and reveals novel candidate genes

We functionally dissected the 12 candidate genes taken from the nine SLE associated loci that showed strong evidence of a shared causal variant with a *cis*-eQTL in LCLs (Table 3). We systematically annotated these genes using a combination of cell/tissue expression patterns, mouse models, known molecular phenotypes, molecular interactions, and associations with other autoimmune diseases (S4 Table). We found the majority of novel SLE candidate genes detected by RNA-Seq were predominately expressed in immune-related tissues such as whole blood as well as the spleen and thymus. Based on our annotation and what is already documented at certain loci, we were sceptical on the pathogenic involvement of three candidate genes (*PHTF1*, *ARHGAP30*, and *RABEP1*). Although the *cis*-eQTL effect for these genes is evidently driven by the shared causal variant as the disease association, it is possible that these effects of expression modulation are merely passengers that are carried on the same functional haplotype as the true causal gene(s) and do not contribute themselves to the breakdown of self-tolerance (detailed in S4 Table). We show the regional association plots and the candidate genes detected from *cis*-eQTL analysis in S4 Fig.

**Table 3:** Nine SLE loci contain cis-eQTLs driven by the same variant as the disease association.

		Gene		Transcript		Exon		Junction		Intron	
Lead SNP	Gene	eQTL *P*^*a*^	JLIM *P*	eQTL *P*	JLIM *P*	eQTL *P*	JLIM *P*	eQTL *P*	JLIM *P*	eQTL *P*	JLIM *P*
rs2476601	*PHTF1*	-	-	2.2 x 10^−3^	6.2 x 10^−1^	5.0 x 10^−8^	1	8.4 x 10^−47^	1	**1.4 x 10**^**−4**^	**1.0 x 10**^**−4**^
rs1801274	*ARHGAP30*	2.4 x 10^−6^	8.1 x 10^−1^	-	-	**1.1 x 10**^**−4**^	**2.0 x 10**^**−4**^	9.4 x 10^−3^	7.4 x 10^−3^	1.2 x 10^−3^	4.8 x 10^−1^
rs9782955	*LYST*	5.4 x 10^−3^	3.90 x 10^−1^	8.0 x 10^−6^	9.8 x 10^−1^	1.6 x 10^−3^	4.6 x 10^−3^	**1.3 x 10**^**−3**^	**2.0 x 10**^**−4**^	1.0 x 10^−5^	5.0 x 10^−1^
rs3768792	*IKZF2*	-	-	1.5 x 10^−3^	7.7 x 10^−1^	**1.9 x 10**^**−4**^	**3.0 x 10**^**−4**^	1.0 x 10^−5^	9.0 x 10^−1^	**1.1 x 10**^**−5**^	**2.0 x 10**^**−4**^
rs10028805	*BANK1*	1.8 x 10^−3^	3.1 x 10^−3^	4.9 x 10^−3^	3.2 x 10^−3^	**1.8 x 10**^**−5**^	**4.0 x 10**^**−4**^	**2.5 x 10**^**−4**^	**2.0 x 10**^**−4**^	1.8 x 10^−4^	9.7 x 10^−1^
rs2736340	*BLK*	**3.2 x 10**^**−26**^	**< 10**^**−4**^	**1.0 x 10**^**−9**^	**< 10**^**−4**^	**1.4 x 10**^**−31**^	**< 10**^**−4**^	**7.6 x 10**^**−28**^	**< 10**^**−4**^	**3.1 x 10**^**−24**^	**< 10**^**−4**^
	*FAM167A*	**2.3 x 10**^**−40**^	**< 10**^**−4**^	**4.4 x 10**^**−45**^	**< 10**^**−4**^	**5.1 x 10**^**−46**^	**< 10**^**−4**^	**1.5 x 10**^**−22**^	**< 10**^**−4**^	**7.4 x 10**^**−15**^	**< 10**^**−4**^
rs2286672	*RABEP1*	1.4 x 10^−3^	5.1 x 10^−2^	1.3 x 10^−4^	9.4 x 10^−1^	**7.4 x 10**^**−5**^	**4.0 x 10**^**−4**^	4.5 x 10^−4^	7.0 x 10^−4^	1.3 x 10^−4^	8.5 x 10^−1^
rs2304256	*TYK2*	1.2 x 10^−3^	7.6 x 10^−1^	9.9 x 10^−6^	9.9 x 10^−1^	**2.5 x 10**^**−9**^	**< 10**^**−4**^	1.3 x 10^−4^	3.0 x 10^−3^	**2.2 x 10**^**−9**^	**2.0 x 10**^**−4**^
	*ATG4D*	-	-	3.8 x 10^−3^	7.2 x 10^−3^	6.4 x 10^−5^	3.8 x 10^−3^	**3.8 x 10**^**−4**^	**2.0 x 10**^**−4**^	6.6 x 10^−5^	9.7 x 10^−1^
rs7444	*UBE2L3*	5.7 x 10^−3^	2.0 x 10^−1^	**5.9 x 10**^**−14**^	**< 10**^**−4**^	**9.9 x 10**^**−5**^	**< 10**^**−4**^	5.1 x 10^−5^	9.5 x 10^−1^	1.2 x 10^−3^	9.0 x 10^−1^
	*CCDC116*	**2.5 x 10**^**−5**^	**5.0 x 10**^**−4**^	1.4 x 10^−6^	3.0 x 10^−4^	**4.9 x 10**^**−4**^	**4.0 x 10**^**−4**^	-	-	-	-

Nine of the 38 SLE loci (24%) were found to be driven by the same causal variant as the disease association across all five RNA-Seq quantification types in LCLs (*cis*-eQTL *P*<0.01 and joint likelihood of shared association FDR<0.05). Bold type indicates associations that show evidence of a shared causal variant for *cis*-eQTL and disease.

^*a*^Minimum *cis*-eQTL *P*-value for any SNP within 100 kb of the lead SNP. Dashes (–) indicate genes that were either not detected or had minimum *cis*-eQTL *P*>0.01 in the RNA-Seq quantification type in hand. JLIM *P*-values <10^−4^ indicates the JLIM statistic is more extreme than permutation. JLIM: joint likelihood mapping. If multiple SNP-unit associations are deemed to be causal (i.e. one SNP shows a causal association to two exons of the same gene, the association with the smallest JLIM *P*-value is reported).

The causal *cis*-eQTL rs2736340 for genes *BLK* and *FAM167A* was detected at all RNA-Seq profiling types. It is well established that the risk allele of this SNP reduces proximal promoter activity of *BLK*; a member of the Src family kinases that functions in intracellular signalling and the regulation of B-cell proliferation, differentiation, and tolerance [26]. The allelic consequence of *FAM167A* expression modulation is unknown. We found multiple instances of known SLE susceptibility genes that were concealed when using gene-level quantification. For example, we defined rs7444 as a causal *cis*-eQTL for *UBE2L3* at transcript- and exon-level—but not at gene-level (Table 3). The risk allele of rs7444 has been associated with increased expression of *UBE3L3* (Ubiquitin conjugating enzyme E2 L3) in *ex vivo* B-cells and monocytes and correlates with NF-κB activation along with increased circulating plasmablast and plasma cell numbers [27]. Similarly, the rs10028805 SNP is a known splicing *cis*-eQTL for *BANK1* (B-cell scaffold protein with ankyrin repeats 1). We replicated at exon-, and junction-level this splicing effect which has been proposed to alter the B-cell activation threshold [28]. Again, this mechanism was not detected using gene-level quantification.

*IKZF2* (detected at the exon-level only) is a transcription factor thought to play a key role in T-reg stabilisation in the presence of inflammatory responses [29]. *IKZF2* deficient mice acquire an auto-inflammatory phenotype in later life similar to rheumatoid arthritis, with increased numbers of activated CD4^+^ and CD8^+^ T-cells, T-follicular helper cells, and germinal centre B-cells, which culminates in autoantibody production [30]. Of note, other members of this gene family, *IKZF1* and *IKZF3*, are also associated with SLE and can hetero-dimerize (S4 Table) [7]. We also believe *LYST*, *ATG4D*, and *TYK2* to also be intriguing candidate genes. *LYST* encodes a lysosomal trafficking regulator [31] whilst *ATG4D* is a cysteine peptidase involved in autophagy and this locus is associated with multiple sclerosis, psoriasis, and rheumatoid arthritis [32]. *TYK2* is discussed in greater detail in the following section.

### RNA-Seq can resolve the potential causal regulatory mechanism(s)

Interestingly, for the three causal SNP-gene pairs detected at gene-level (rs2736340 –*BLK*, rs2736340 –*FAM167A*, and rs7444 –*CCDC116*), we found that at exon-level, all expressed exons possessed causal *cis*-eQTLs. For example, rs2736340 is a causal *cis*-eQTL for all thirteen exons of *BLK* and for all three exons of *FAM167A* (S5 Table). These data suggest that gene-level analysis is capturing associations where all—or the majority of exons—are modulated by the *cis*-eQTL.

We found that within the SLE associated loci that showed evidence of a shared causal variant with a *cis*-eQTL (Table 3), there were many instances in which the proposed causal *cis*-eQTL modulated expression of only a single expression element. This enabled us to resolve the potential regulatory effect of the causal *cis*-eQTL to a particular transcript, exon, junction, or intron (S5 Table). We were able to resolve to a single expression element in nine of the twelve candidate SNP-gene pairs. For example, rs9782955 is a causal *cis*-eQTL for *LYST* at junction-level for only a single junction (chr1:235915471–235916344; *cis*-eQTL *P* = 1.3x10^-03^; JLIM *P* = 2.0x10^-04^). We provide depicted examples of this isolation analysis for candidate genes *IKZF2* (S5 Fig), *UBE2L3* (S6 Fig), and *LYST* (S7 Fig).

**Fig 2:**
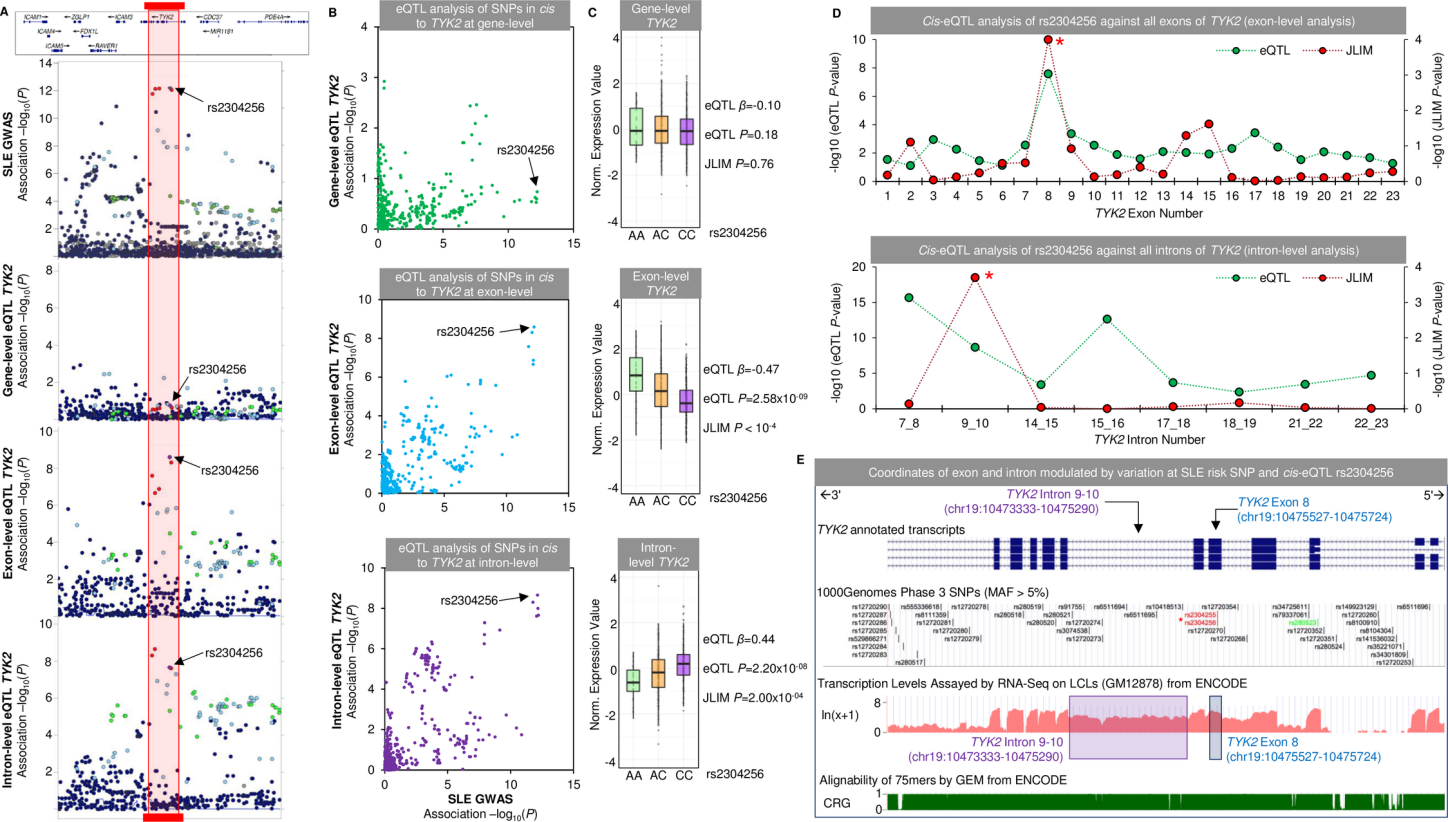
Isolation of potential causal molecular mechanism in *TYK2* by SLE cis-eQTL rs2304256. (A) SLE GWAS association plot and *cis*-eQTL association plot around the 19p13.2 susceptibility locus tagged by rs2304256. The top panel shows the association plot with SLE that spans the gene body and 3′ region of *TYK2* (Tyrosine Kinase 2). The haplotype block composed of highly correlated SNPs is highlighted in the red block. The second panel shows the *cis*-eQTL association plot at gene-level of all proximal SNPs to *TYK2* (no significant association with rs2304256 is detected). The third panel shows the same regional association but at exon-level for the most associated exon of *TYK2* with rs2304256 –the bottom panel is at intron-level for *TYK2* (both are highly associated). (B) Correlation of SLE GWAS *P*-value and *cis*-eQTL association *P*-value for all SNPs in *cis* to *TYK2*. We show at gene-level the most associated SLE SNPs are not *cis*-eQTLs (top panel). The middle and bottom panels show the same correlation at exon-level and intron-level and reveal the most associated SNPs to SLE are also the most associated *cis*-eQTLs to *TYK2*. (C) The direction of effect of *cis*-eQTL rs2304256 with *TYK2* at gene-level (top), exon-level (middle), and intron-level (bottom panel). The risk allele is rs2304256 [C]. (D) The top panel shows *cis*-eQTL association and JLIM *P*-values for all exons of *TYK2* against rs2304256. Exon 8 (marked by an asterisk) is defined as having a causal association with rs2304256. The bottom panel shows the intron-level *cis*-eQTL of *TYK2* against rs2304256. Note many introns are *cis*-eQTLs but are not causal with rs2304256. Exons and introns are numbered consecutively from start to end of gene if they are expressed (note some are not and therefore not included). (E) The genomic location of the single exon and single intron of *TYK2* that are modulated by rs2304256 are highlighted (rs2304256 is marked by an asterisk in red). The bottom two panels show the transcription levels assayed by RNA-Seq on LCLs assayed by ENCODE. Note intron 9–10 of *TYK2* is clearly expressed. The alignability of 75-mers by GEM is also shown to show the mapability of reads around rs2304256.

We provide a worked example of resolving the causal mechanism(s) using RNA-Seq for the novel association rs2304256 with *TYK2* (Fig 2). The top panel of Fig 2A shows the genetic association to SLE at the 19p13.2 susceptibility locus tagged by lead SNP rs2304256 (*P* = 1.54x10^-12^). Multiple tightly correlated SNPs span the gene body and the 3′ region of *TYK2* –which encodes Tyrosine Kinase 2—thought to be involved in the initiation of type I IFN signalling [33]. In the panel below, we plot the gene-level association of all SNPs in *cis* to *TYK2* and show no significant association of rs3204256 with *TYK2* expression (*P* = 0.18). At exon-, and intron-level, we were able to classify rs2304256 as a causal *cis*-eQTL for a single exon (chr19: 10475527–10475724; *cis*-eQTL *P* = 2.58x10^-09^; JLIM *P*<10^−04^) and a single intron (chr19: 10473333–10475290; *P* = 2.20x10^-08^; JLIM *P* = 2x10^-04^) of *TYK2* respectively as shown in the bottom two panels of Fig 2A. We show the exon and intron labelling of *TYK2* in further detail in S8 Fig. We found strong correlation of association *P*-values of the SLE GWAS and the *P*-values of *TYK2 cis*-eQTLs against at exon-level and intron-level, but not at gene-level (Fig 2B). The risk allele rs2304256 [C] was found to be associated with decreased expression of the *TYK2* exon and increased expression of the *TYK2* intron (Fig 2C). By plotting the *cis*-eQTL *P*-values alongside the JLIM *P*-values for all exons and introns of *TYK2* against rs2304256 (Fig 2D), we clearly show that only a single exon and a single intron of *TYK2* colocalize with the SLE association signal–marked by an asterisk (note that rs2304256 is a strong *cis*-eQTL for many introns of *TYK2* but only shares a causal variant with one intron). We show the genomic location of the affected exon and intron of *TYK2* in Fig 2E (exon 8 and the intron between exons 9 and 10). Intron 9–10 of *TYK2* is clearly expressed in LCLs according to transcription levels assayed by RNA-Seq on LCLs (GM12878) from ENCODE (Fig 2E).

Interestingly, rs2304256 (marked by an asterisk in Fig 2E) is a missense variant (V362F) within exon 8 of *TYK2*. The PolyPhen prediction of this substitution is predicted to be benign and, to the best of our knowledge, no investigation has isolated the functional effect of this particular amino acid change. We do not believe the *cis*-eQTL at exon 8 to be a result of variation at rs3204256 and mapping biases, as the alignability of 75mers by GEM from ENCODE is predicted to be robust around exon 8 (Fig 2E). In fact, rs3204256 [C] is the reference allele yet is associated with decreased expression of exon 8.

In conclusion, we have found an interesting and novel mechanism that would have been concealed by gene-level analysis that involves the risk allele of a missense SNP associated with decreased expression of a single exon of *TYK2* but increased expression of the neighbouring intron. Whether the *cis*-eQTL effect and missense variation act in a combinatorial manner and whether the intron is truly retained or if it is derived from an unannotated transcript of *TYK2* is an interesting line of investigation.

### Detection of cis-eQTLs and candidate-genes of autoimmune disease using RNA-Seq

We re-performed our integrative *cis*-eQTL analysis with the Geuvadis RNA-Seq dataset in LCLs using association data from twenty autoimmune diseases. This was to firstly reiterate the importance of leveraging RNA-Seq in GWAS interpretation and to secondly demonstrate that our findings in SLE persisted across other immunological traits. As the raw genetic association data were not available for all twenty diseases, we were unable to implement the JLIM pipeline which requires densely typed or imputed GWAS summary-level statistics. We therefore opted to use the Regulatory Trait Concordance (RTC) method, which requires full genotype-level data for the expression trait, but only the marker identifier for the lead SNP of the disease association trait (see methods for a description of the RTC method). We stringently controlled our integrative *cis*-eQTL analysis for multiple testing to limit potential false positive findings of overlapping association signals. To do this, we applied a Bonferroni correction to nominal *cis*-eQTL *P*-values separately per disease and per RNA-Seq quantification type. We rigorously defined causal *cis*-eQTLs, as associations with *P*_BF_ < 0.05 and RTC ≥ 0.95. An overview of the analysis pipeline is depicted in S9 Fig and S10 Fig. Using an *r*^*2*^ cut-off of 0.8 and a 100kb limit, we pruned the 752 associated SNPs from the twenty human autoimmune diseases from the Immunobase resource (S6 Table) to obtain 560 independent susceptibility loci.

**Fig 3:**
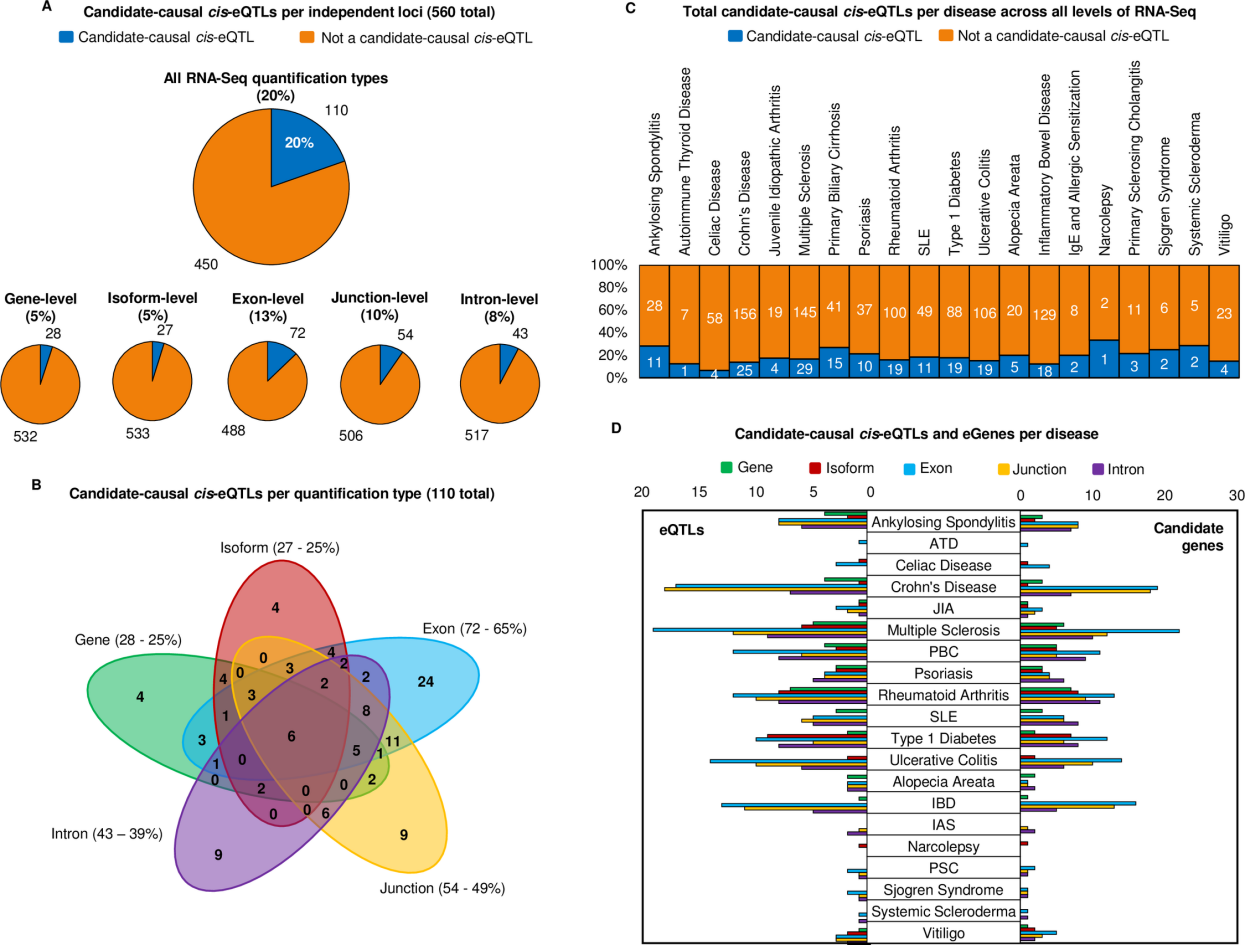
Breakdown of autoimmune associated causal cis-eQTLs using RNA-Seq. (A) Percentage and number of causal *cis*-eQTL associations detected per RNA-Seq quantification type, following LD pruning of associated SNPs from twenty autoimmune diseases to 560 independent susceptibly loci. The top chart shows the number of causal *cis*-eQTLs when combining all RNA-Seq profiling types together (20%). (B) Sharing of causal *cis*-eQTL associations per quantification type (110 detected in total). Percentage of causal *cis*-eQTLs captured are shown as a percentage of the 110 total. (C) Total causal *cis*-eQTLs per disease across all five levels of RNA-Seq quantification, using the 20 diseases of the ImmunoBase resource. In orange are disease-associated SNPs that show no shared association with expression across any quantification type. In blue are the disease-associated SNPs that are also causal *cis*-eQTLs. (D) Causal *cis*-eQTLs and candidate genes per disease broken down by quantification type.

Our findings confirmed our previous results from the SLE investigation, and again support the gene-level study using the JLIM package. As before, we found that only 5% (28 of the 560 loci) of autoimmune susceptibility loci were deemed to share causal variants with *cis*-eQTLs using either gene- or transcript-level analysis (Fig 3A). Exon-level analysis more than doubled the yield to 13% (72 of the 560 loci) with junction-, and intron-level analysis also outperforming gene-level (10% and 8% respectively). When combining all RNA-Seq quantification types, we could define 20% of autoimmune associated loci (110 of the 560 loci) as being candidate causal *cis*-eQTLs—which corroborates our previous estimate in SLE using JLIM (24%).

By separating causal *cis*-eQTL associations out by quantification type, we found over half (65%) were detected at exon-level, and considerable overlap of *cis*-eQTL associations existed between both types (Fig 3B). Unlike in our SLE analysis, gene- and isoform-level analysis did capture a small fraction of causal *cis*-eQTLs that were not captured at exon-level. Our data therefore suggest that although exon- and junction-level, and to a lesser extent intron-level analysis, capture most candidate-causal *cis*-eQTLs. It is necessary to prolife gene-expression at all quantification types to avoid misinterpretation of the functional impact of disease associated SNPs.

We mapped the causal *cis*-eQTLs detected by all RNA-Seq quantification types back to the diseases to which they are associated (Fig 3C). Interestingly, we observed the diseases that fell below the 20% average comprised autoimmune disorders related to the gut: celiac disease (7%), inflammatory bowel disease (14%), Crohn’s disease (16%), and ulcerative colitis (18%). We attribute this observation as a result of the cellular expression specificity of associated genes in colonic tissue and in T-cells [34]. Correspondingly, we observed an above-average frequency of causal *cis*-eQTLs detected in SLE (22%) and primary biliary cirrhosis (37%); diseases in which the pathogenic role of B-lymphocytes and autoantibody production is well documented [34]. Note that there are 60 SLE GWAS associations in this analysis as these originate from three independent GWA studies (S6 Table). We further broke down our results per disease by RNA-Seq quantification type (Fig 3D) and in all cases, the greatest frequency of causal *cis*-eQTLs and candidate genes were captured by exon- and junction-level analyses.

### Web resource for functional interpretation of association studies of autoimmune disease

We provide the results from our analysis as a web resource (found at www.insidegen.com) for researchers to lookup causal *cis*-eQTLs and candidate genes from the twenty autoimmune diseases detected across the five RNA-Seq quantification types. The data are sub-settable and exportable by SNP ID, gene, RNA-Seq resolution, genomic position, and association to specific autoimmune diseases. See methods for a walkthrough of how to access results.

### Exon-level quantification detects systematic and heterogeneous effects on gene expression

By implementing a mixed model test of heterogeneity that accounts for the dependency structure arising from within-individual and within-gene expression correlations, we attempted to distinguish causal *cis*-eQTLs at transcript-, exon-, junction-, and intron-level that fitted either a systematic gene-model (characterized by a similar effect on expression across all elements within a gene) or a heterogeneous gene-model (where the *cis*-eQTL signal is only evident in a subset of expression elements). The full results of this analysis are found in S7 Table.

**Fig 4:**
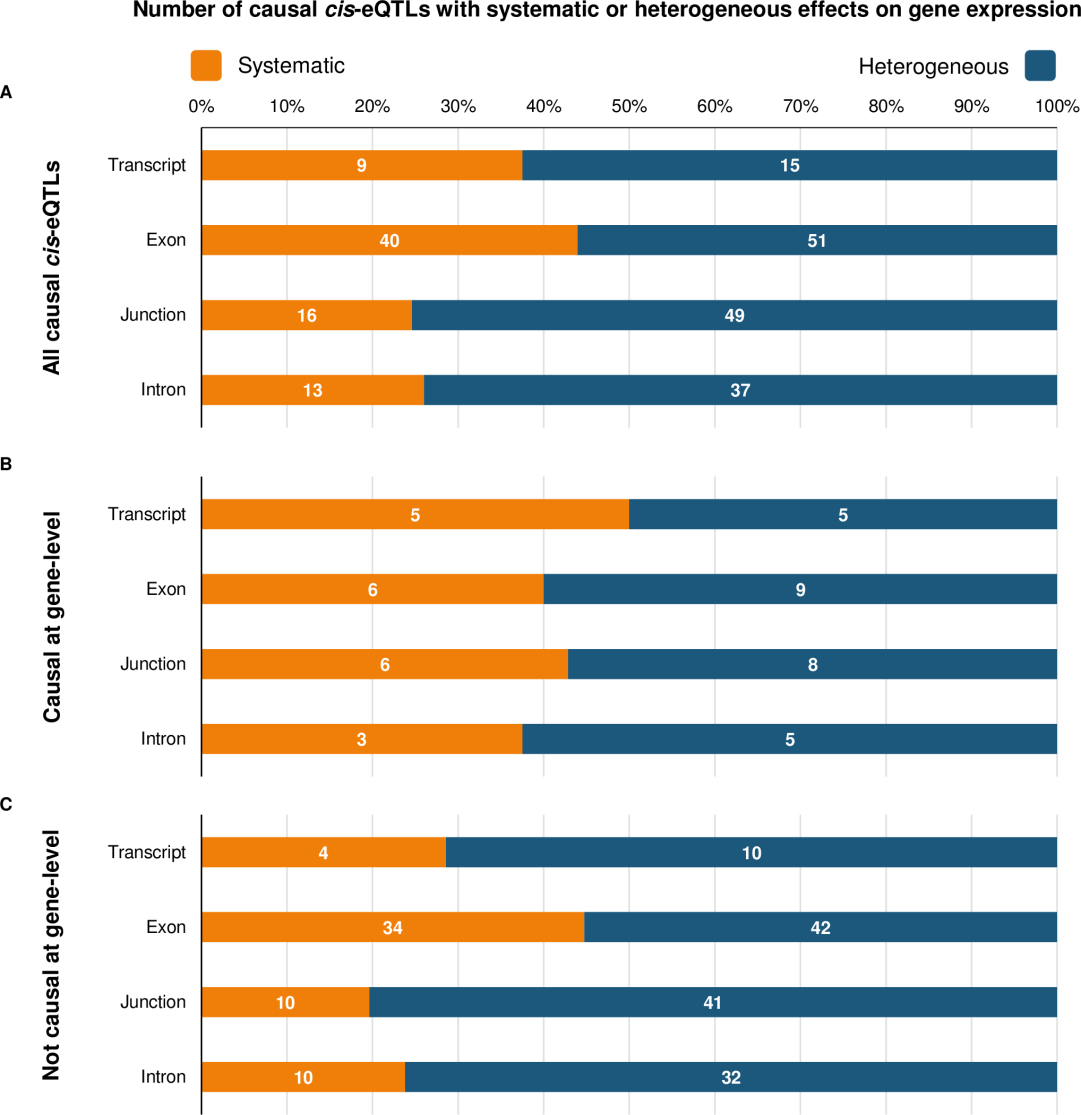
Number of causal cis-eQTLs with systematic or heterogeneous effects. (A) Using a modified test of heterogeneity that accounts for the dependency structure arising from within-individual and within-gene expression correlations, we distinguished causal *cis*-eQTLs that fitted either a systematic gene-model (orange) or a heterogeneous gene-model (blue) per quantification type. The full results of this analysis are found in S7 Table. Numbers represent the total number of SNP-gene associations per quantification type. (B) Causal *cis*-eQTLs that are also causal *cis*-eQTLs at gene-level. (C) Causal *cis*-eQTLs that are not causal *cis*-eQTLs at gene-level.

**Fig 5:**
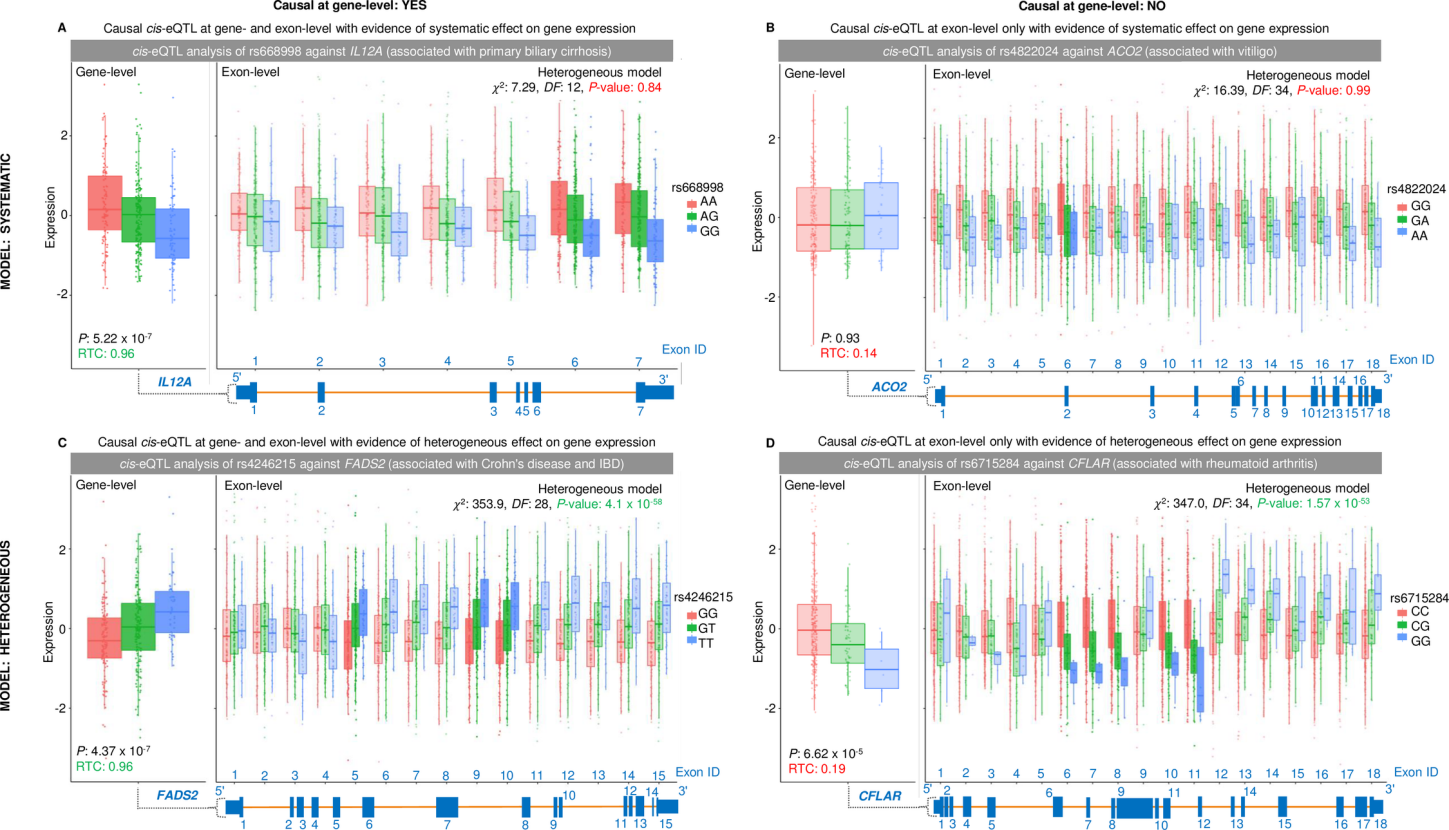
Examples of causal cis-eQTLs with systematic or heterogeneous effects on expression. This figure shows exon-level analysis using a modified test of heterogeneity to distinguish systematic causal *cis*-eQTLs and heterogeneous *cis*-eQTLs. It then stratifies these results based on whether the association is detected at gene-level or not. Each panel shows the gene-level association with *cis*-eQTL association *P*-value and RTC score (RTC > = 0.95 is deemed causal, highlighted in green), the exon-level association for each exon of the gene against the *cis*-eQTL, the heterogeneous model output from the likelihood ratio test with χ^2^ statistic, degrees of freedom (DF), and model *P*-value (highlighted in red is heterogeneous, green is systematic), and finally the collapsed gene model underneath with labelled exons. N.B box-plots in a darker shade are those that are deemed to be causal associations (*P*_BF_ < 0.05 & RTC > = 0.95). (A) Systematic *cis*-eQTL detected at gene-level (B) systematic *cis*-eQTL not detected at gene-level (C) heterogeneous *cis*-eQTL detected at gene-level (D) heterogeneous *cis*-eQTL not detected at gene-level.

We found that across each RNA-Seq profiling type, the majority of causal *cis*-eQTLs exhibited heterogeneous effects on gene expression; indicative of alternative isoform usage (Fig 4A). Junction-level causal *cis*-eQTLs had the greatest proportion of heterogeneous associations (49 of 65 causal cis-eQTLs were heterogeneous—75%). Both systematic and heterogeneous causal *cis*-eQTLs were then stratified by whether or not they were also causal at gene-level. As expected, we observed that causal *cis*-eQTLs that were also detected at gene-level (Fig 4B) showed a greater proportion of systematic effects on gene expression than associations not detected at gene-level (Fig 4C). In both cases however, the heterogeneous model was more apposite. Interestingly, we found that the greatest frequency of systematic associations, which were not captured at gene-level, were observed at exon-level (42 of 76: 55%). This implies that exon-level analysis captures a near equal proportion of both systematic and heterogeneous effects that are not detected by gene-level analysis. We show four examples of systematic and heterogeneous causal *cis*-eQTLs stratified by their detection at gene-level quantification in Fig 5.

### Causal cis-eQTLs localise to discrete chromatin regulatory elements

**Fig 6:**
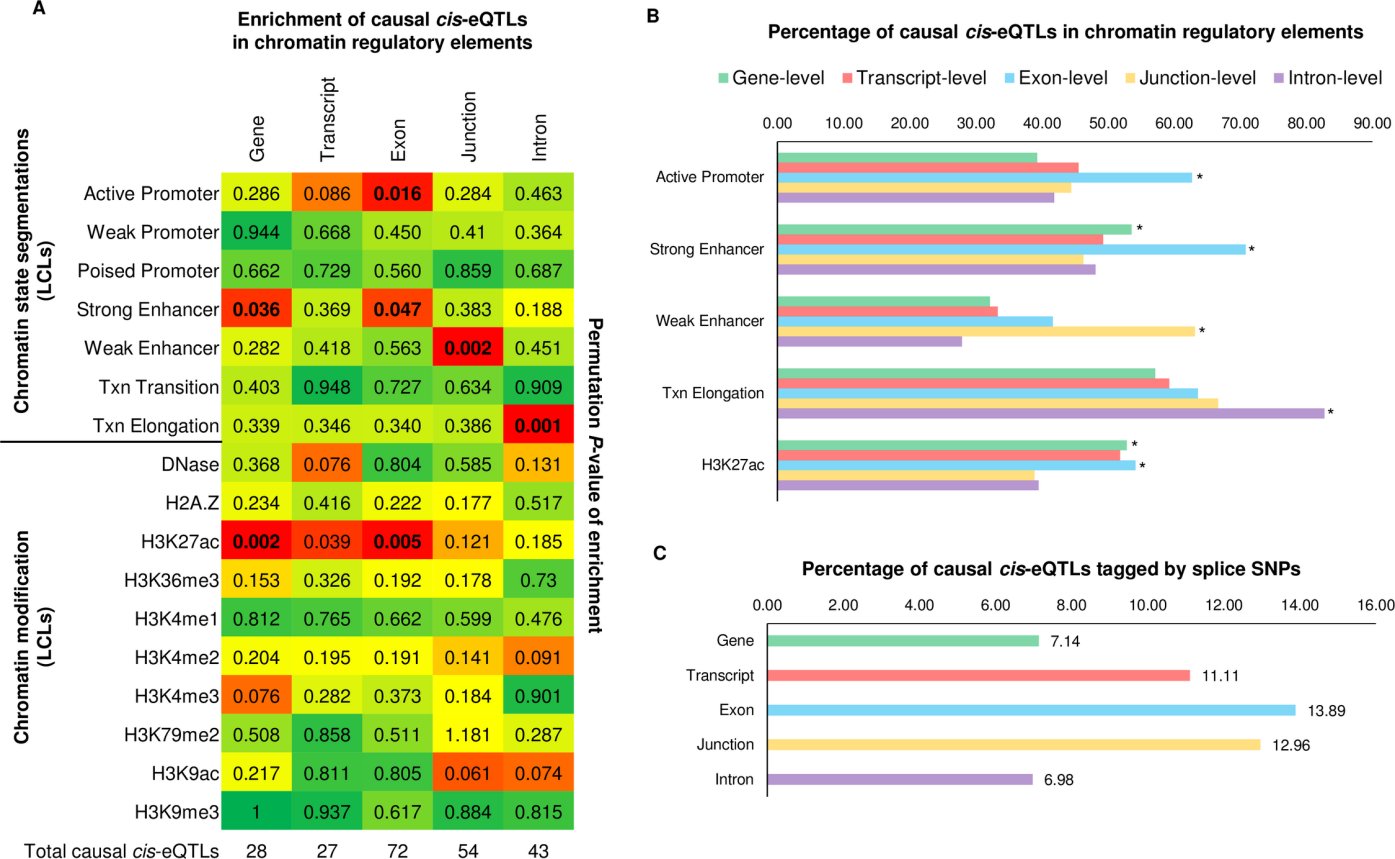
Functional annotation of causal autoimmune cis-eQTLs. (A) We took the causal autoimmune *cis*-eQTLs detected for each RNA-Seq quantification type and performed enrichment testing for chromatin state segmentation and histone marks in LCLs taken from the NIH Roadmap Epigenomics Project. We used the GoShifter algorithm to do this (see methods); which takes all SNPs in strong LD (*r*^2^>0.8) with the causal *cis*-eQTLs and calculates the proportion of SNPs overlapping chromatin marks, the positions of the marks are then shuffled whilst retaining the SNP positions, and the fraction of overlap recalculated over 1,000 permutations. A permutation *P*-value is then generated–which is annotated in each box (*P*<0.05 deemed significant). The heat colour is representative of the permutation *P*-value. Significant enrichment tests are highlighted in bold. The total number of causal *cis*-eQTLs per quantification type are annotated at the bottom of the heatmap. (B) The percentage of causal *cis*-eQTLs in chromatin regulatory marks per quantification type. An asterisk shows that this level of enrichment is deemed to be significant as shown in panel A. (C) The percentage of causal *cis*-eQTLs in chromatin regulatory marks per quantification type that are or are highly correlated (*r*^2^>0.8) with SNPs that alter splice site consensus sequences of the target genes (assessed by Sequence Ontology for the hg19 GENCODE v12 reference annotation).

A previous investigation has suggested that causal variants of gene-level and transcript-level *cis*-eQTLs reside in discrete functional elements of the genome [18]. We therefore investigated whether this notion held true across the five RNA-Seq quantification types tested in this study. To accomplish this, we selected the causal *cis*-eQTLs from the twenty autoimmune diseases interrogated, and per quantification type, tested for enrichment of these SNPs across various chromatin regulatory elements taken from the Roadmap Epigenomics Project in LCLs (using both the Roadmap chromatin state model and the positions of histone modifications). We implemented the permutation-based GoShifter algorithm to test for enrichment of causal *cis*-eQTLs and tightly correlated variants (*r*^2^>0.8) in genomic functional annotations in LCLs (see methods) [25]. Results of this analysis are depicted in Fig 6. We found the 28 gene-level *cis*-eQTLs were enriched in two chromatin marks: strong enhancers (*P* = 0.036) and H3K27ac occupancy sites–a marker of active enhancers (*P* = 0.002). Transcript-level *cis*-eQTLs were also enriched in H3K27ac occupancy sites (*P* = 0.039) but were not enriched in any other marks. The 72 exon-level *cis*-eQTLs were additionally enriched in active promoters (*P* = 0.017). Interestingly, the 54 causal *cis*-eQTLs detected at junction-level were found to be enriched in weak enhancers only (*P* = 0.002); whilst the 43 intron-level *cis*-eQTLs were enriched in chromatin states predicted to be involved in transcriptional elongation (*P* = 0.001; 83% of intron-level *cis*-eQTLs). Disease relevant *cis*-eQTLs detected at different expression phenotypes using RNA-Seq clearly localise to largely discrete functional elements of the genome.

We quantified the number of causal *cis*-eQTLs and tightly correlated variants (*r*^2^>0.8) per quantification type that were predicted to be alter splice site consensus sequences of the target genes (assessed by Sequence Ontology for the hg19 GENCODE v12 reference annotation). We found only two of the 28 (7%) gene-level *cis*-eQTLs disrupted consensus splice-sites for their target genes compared to the 14% and 13% detected at exon- and junction-level respectively (Fig 6C). Our data suggest that although exon- and junction- level analysis leads to the greatest frequency of causal *cis*-eQTLs, the majority at this resolution cannot be explained directly by variation in annotated splice site consensus sequences (splice region/donor/acceptor/ variants).

### Confirmation that autoimmune causal cis-eQTLs reach genome-wide level of significance

We extended our investigation and performed genome-wide *cis*-eQTL analysis for all SNPs against gene-, transcript-, exon-, junction-, and intron-level quantifications. As with our integrative analysis of autoimmune risk loci, we found the greatest number of genome-wide significant *cis*-eQTLs and target genes (at a genome-wide FDR threshold of 5%) were detected at exon-level, followed by junction- and intron-level; with gene- and transcript-level being thoroughly outperformed (S8 Table and S11 Fig). We confirmed that all of the causal *cis*-eQTL associations detected in our integrative analysis with autoimmune risk loci reached genome-wide significance—owing to the stringent Bonferroni multiple testing correction adopted (S9 Table).

## Discussion

Elucidation of the functional consequences of non-coding genetic variation in human disease is a major objective of medical genomics [35]. Integrative studies that map disease-associated eQTLs in relevant cell types and physiological conditions are proving essential in progression towards this goal through identification of causal SNPs, candidate-genes, and illumination of molecular mechanisms [36]. In autoimmune disease, where there is considerable overlap of immunopathology, integrative eQTL investigations have been able to connect discrete aetiological pathways, cell types, and epigenetic modifications, to particular clinical manifestations [2,34,36,37]. Emerging evidence however has suggested that only a minority (~25%) of autoimmune associated SNPs share casual variants with basal-level *cis*-eQTLs in primary immune cell-types [9].

Genetic variation can influence expression at every stage of the gene regulatory cascade—from chromatin dynamics, to RNA folding, stability, and splicing, and protein translation [21]. It is now well documented that SNPs affecting these units of expression vary strikingly in their genomic positions and localisation to specific epigenetic marks [18]. The eQTLs that affect pre-transcriptional regulation—affecting all isoforms of a gene—differ in the proximity to the target gene and effect on translated isoforms than their co-transcriptional trQTL (transcript ratio QTL) counterparts. Where the effect size of eQTLs generally increases in relation to transcription start site proximity, trQTLs are distributed across the transcript body and generally localise to intronic binding sites of splicing factors [18,21]. In over 57% of genes with both an eQTL influencing overall gene expression and an trQTL affecting the ratio of each transcript to the gene total, the causal variants for each effect are independent and reside in distinct regulatory elements of the genome [18]. In fact, three primary molecular mechanisms are thought to link common genetic variants to complex traits. A large proportion of trait associated SNPs act via direct effects on pre-mRNA splicing that do not change total mRNA levels [21]. Common variants also act via alteration of pre-mRNA splicing indirectly through effects on chromatin dynamics and accessibility. Such chromatin accessibility QTLs are however more likely to alter total mRNA levels than splicing ratios. Lastly, it is thought that only a minority of trait associated variants have direct effects on total gene expression that cannot be explained by changes in chromatin. As RNA-Seq becomes the convention for genome-wide transcriptomics, it is essential to maximise its ability to resolve and quantify discrete transcriptomic features so to expose the genetic variants that contribute to changes in expression and isoform usage. The reasoning for our investigation therefore was to delineate the limits of microarray and RNA-Seq based eQTL cohorts in the functional annotation of autoimmune disease association signals.

To map autoimmune disease associated *cis*-eQTLs, we interrogated RNA-Seq expression data profiled at gene-, isoform, exon-, junction-, and intron-level, and tested for a shared genetic effect at each significant association. As we had densely imputed summary statistics from our SLE GWAS, we opted to use the Joint Likelihood Mapping (JLIM) framework [9] to test for a shared causal variant between the disease and *cis*-eQTL signals. This framework has been rigorously benchmarked against other colocalisation procedures. Summary statistics were not available for the remaining autoimmune diseases and therefore we implemented the Regulatory Trait Concordance (RTC) method for these diseases and set a stringent multiple testing threshold to define causal *cis*-eQTLs. We found the estimates of causal *cis*-eQTLs were near identical between the two methods used (Table 1 and Fig 3A). Exon- and junction-level quantification led to the greatest frequency of causal *cis*-eQTLs and candidate genes (exon-level: 13–18%, junction-level: 10–11%). We conclusively found that associated variants were in fact more likely to colocalize with exon- and junction-level *cis*-eQTLs when applying a nominal JLIM *P*-value threshold of <0.01 (Fig 1B and Table 2). Gene-level analysis was thoroughly outperformed in all cases (5%). Our findings that gene-level analysis explain only 5% of causal *cis*-eQTLs corroborate the findings from *Chun et al* [9] who composed and used the JLIM framework to annotate variants associated with seven autoimmune diseases (multiple sclerosis, IBD, Crohn’s disease, ulcerative colitis, T1D, rheumatoid arthritis, and celiac disease). They found that only 16 of the 272 autoimmune associated loci (6%) shared causal variants with *cis*-eQTLs using gene-level RNA-Seq (with the same Geuvadis European cohort in LCLs as used herein). In our investigation, we argue that it is necessary to profile expression at all possible resolutions to diminish the likelihood of overlooking potentially causal *cis*-eQTLs. In fact, by combining our results across all resolutions, we found that 20–24% of autoimmune loci were candidate-causal *cis*-eQTLs for at least one target gene. Our study therefore increases the number of autoimmune loci with shared genetic effects with *cis*-eQTLs in a single cell type by over four-fold. Interestingly, using microarray data from CD4^+^ T-cells *Chun et al* classified 37 of the 272 autoimmune loci (14%) as causal *cis*-eQTLs [9]—strengthening the hypothesis that autoimmune loci (especially those associated with inflammatory diseases of the gut) are enriched in CD4^+^ T-cell subsets and the cells themselves are likely to be pathogenic [25,34]. Microarray data are known to underestimate the number of true causal *cis*-eQTLs [10]. If we assume that by leveraging RNA-Seq we can increase the number of steady-state causal *cis*-eQTLs four-fold, we hypothesise that as many as ~54% of autoimmune loci may share causal *cis*-eQTLs with gene expression at multiple resolutions in CD4^+^ T-cell populations. A large RNA-Seq based eQTL cohort profiled across multiple CD4^+^ T-cell subsets will therefore be of great use when annotating autoimmune-related traits. Immune activation conditions further increase the number of causal *cis*-eQTLs detected in autoimmune disease [38]. We reason that although using relevant cell types and context-specific conditions will undoubtedly increase our understanding of how associated variants alter cell physiology and ultimately contribute to disease risk; it is clearly shown herein that we are only picking the low hanging fruit in current eQTL analyses. We argue it necessary to reanalyse existing RNA-Seq based eQTL cohorts at multiple resolutions and ensure new datasets are similarly dissected. Despite the severe multiple testing burden, we also argue that expression profiling at multiple resolutions using RNA-Seq may be advantageous even when looking for *trans*-eQTL effects. As *trans*-eQTLs are generally more cell-type specific and have a weaker effect size, we decided not to perform such analyses using the Geuvadis LCL data. Large RNA-Seq based eQTL cohorts in whole-blood will be more suitable for such analysis [19].

As well as biological reasons for using multiple expression phenotypes for integrative eQTL analysis, there are also technical factors to consider. Gene-level expression estimates can generally be obtained in two ways–union-exon based approaches [14,17] and transcript-based approaches [11,12]. In the former, all overlapping exons of the same gene are merged into union exons, and intersecting exon and junction reads (including split-reads) are counted to these pseudo-gene boundaries. Using this counting-based approach, it is also possible to quantify meta-exons and junctions easily and with high confidence by preparing the reference annotation appropriately [13,15,39]. Introns can be quantified in a similar manner by inverting the reference annotation between exons and introns [18]. Of note, we found intron-level quantification generated more candidate-causal *cis*-eQTLs than gene-level (Fig 3A). As the library was synthesised from poly-A selection, these associations are unlikely due to differences in pre-mRNA abundance. Rather, they are likely derived from either true retained introns in the mature RNA or from coding exons that are not documented in the reference annotation used. Transcript-based approaches make use of statistical models and expectation maximization algorithms to distribute reads among gene isoforms—resulting in isoform expression estimates [11,12]. These estimates can then be summed to obtain the entire expression estimate of the gene. Greater biological insight is gained from isoform-level analysis; however, disambiguation of specific transcripts is not trivial due to substantial sequence commonality of exons and junctions. In fact, we found only 5% of autoimmune loci shared a causal variant at transcript-level.

The different approaches used to estimate expression can also lead to significant differences in the reported counts. Union-based approaches, whilst computationally less expensive, can underestimate expression levels relative to transcript-based, and this difference becomes more pronounced when the number of isoforms of a gene increases, and when expression is primarily derived from shorter isoforms [20]. The Geuvadis study implemented a transcript-based approach to obtain whole-gene expression estimates. Clearly therefore, a gold standard of reference annotation and eQTL mapping using RNA-Seq is essential for comparative analysis across datasets. Our findings support recent evidence that suggests exon-level based strategies are more sensitive and specific than conventional gene-level approaches [22]. Subtle isoform variation and expression of less abundant isoforms are likely to be masked by gene-level analysis. Exon-level allows for detection of moderate but systematic changes in gene expression that are not captured at gene-level, and also, gene-level summary counts can be shifted in the direction of extreme exon outliers [22]. It is therefore important to note that a positive exon-level eQTL association does not necessarily mean a differential exon-usage or splicing mechanism is involved; rather a systematic expression effect across the whole gene may exist that is only captured by the increased sensitivity. By implementing a mixed model test of heterogeneity that accounts for the dependency structure arising from within-individual and within-gene expression correlations we found that causal *cis*-eQTLs captured by exon-level analysis that are not detected at gene-level, are derived from both systematic and heterogeneous effects on gene expression in almost equal proportions (Fig 4). Additionally, by combining exon-level with other RNA-Seq quantification types, inferences can be made on the particular isoforms and functional domains affected by the eQTL which can later aid biological interpretation and targeted follow-up investigations [10]. We clearly show this from our analysis of SLE candidate genes *IKZF2* (S5 Fig), *UBE2L3* (S6 Fig), *LYST* (S7 Fig) and *TYK2* (Fig 2). For *TYK2* we reveal a novel mechanism whereby the associated variant rs2304256 [C] leads to decreased expression of a single exon and increased expression of a neighbouring intron (Fig 2). By isolating particular exons, junctions, and introns, one can design more refined follow-up investigations to study the functional impact of non-coding disease associated variants. We show how our findings can be leveraged to comprehensively examine GWAS results of autoimmune diseases. We found nine of the 38 SLE susceptibility loci were causal *cis*-eQTLs (Table 3) for 12 candidate genes which we later functionally annotated in detail (S4 Table).

Taken together, we have provided a deeper mechanistic understanding of the genetic regulation of gene expression in autoimmune disease by profiling the transcriptome at multiple resolutions using RNA-Seq. Similar analyses leveraging RNA-Seq in new and existing datasets using relevant cell types and context-specific conditions (such as response eQTLs as shown in [38]) will undoubtedly increase our understanding of how associated variants alter cell physiology and ultimately contribute to disease risk.

## Materials and methods

### RNA-Sequencing expression data in lymphoblastoid cell lines

RNA-Sequencing (RNA-Seq) expression data from 373 lymphoblastoid cell lines (LCLs) derived from four European sub-populations (Utah Residents with Northern and Western European Ancestry, British in England and Scotland, Finnish in Finland, and Toscani in Italia) of the Geuvadis project [18] were obtained from the EBI ArrayExpress website under accession: E-GEUV-1. The 89 individuals of the Geuvadis project from the Yoruba in Ibadan, Nigeria were excluded from this analysis. All individuals were included as part of the 1000Genomes Project. Expression was profiled using RNA-Seq at five quantification types: gene-, transcript-, exon-, junction-, and intron-level (the files downloaded and used in this analysis have the suffix: ‘QuantCount.45N.50FN.samplename.resk10.txt.gz’). Full methods of expression quantification can be found in the original publication and on the Geuvadis wiki page: http://geuvadiswiki.crg.es/)). We have also provided a breakdown of the quantification methods in S1 Fig. Expression data downloaded represent quantifications that are corrected for sequencing depth and gene/exon etc length (RPKM). Only expression elements quantified in >50% of individuals were kept and Probabilistic Estimation of Expression Residuals (PEER) had been used to remove technical variation [40]. We transformed all expression data to a standard normal distribution.

In summary, transcripts, splice-junctions, and introns were quantified using Flux Capacitor against the GENCODE v12 basic reference annotation [16]. Reads belonging to single transcripts were predicted by deconvolution per observations of paired-reads mapping across all exonic segments of a locus. Gene-level expression was calculated as the sum of all transcripts per gene. Annotated splice junctions were quantified using split read information, counting the number of reads supporting a given junction. Intronic regions that are not retained in any mature annotated transcript, and reported mapped reads in different bins across the intron to distinguish reads stemming from retained introns from those produced by not yet annotated exons. Meta-exons were quantified by merging all overlapping exonic portions of a gene into non-redundant units and counting reads within these bins. Reads were excluded when the read pairs map to two different genes.

### SLE associated SNPs

SNPs genetically associated to systemic lupus erythematosus (SLE) were taken from the *Bentham and Morris et al 2015* GWAS in persons of European descent [7]. The study comprised a primary GWAS, with validation through meta-analysis and replication study in an external cohort (7,219 cases, 15,991 controls in total). Independently associated susceptibility loci taken forward for this investigation were those that passed either genome-wide significance (*P*<5x10^-08^) in the primary GWAS or meta-analysis and/or those that reached significance in the replication study (q<0.01). We defined the lead SNP at each locus as either being the SNP with the lowest *P*-value post meta-analysis or the SNP with the greatest evidence of a missense effect as defined by a Bayes Factor (see original publication). We omitted non-autosomal associations and those within the Major Histocompatibility Complex (MHC), and SNPs with a minor allele frequency (MAF) < 0.05. In total, 38 independently associated SLE associated GWAS SNPs were taken forward for investigation (S1 Table). Each susceptibility locus had previously been imputed to the level of 1000 Genomes Phase3 using a combination of pre-phasing by the SHAPEIT algorithm and imputation by IMPUTE (see original publication for full details) [7].

### Cis-eQTL analysis and Joint Likelihood Mapping (JLIM) of SLE associated SNPs

Primary trait summary statistics file. A JLIM index file for each of the 38 SLE associated SNPs was firstly generated by taking the position of each SNP (hg19) and a creating a 100kb interval in both directions. Summary-level association statistics were obtained form the *Bentham and Morris et al* 2015 European SLE GWAS (imputed to 1000Genomes Phase 3). We downloaded summary-level association data (chromosome, position, SNP, *P*-value) for all directly typed or imputed SNPs with an IMPUTE info score ≥0.7 within each of the 38 intervals. The two-sided *P*-value was transformed into a *Z*-statistic as described by JLIM.

Reference LD file. Genotype files in VCF format for all 373 European individuals of the Geuvadis RNA-Seq project were obtained from the EBI ArrayExpress under accession: E-GEUV-1. The 41 individuals genotyped on the Omni 2.5M SNP array had been previously imputed to the Phase 1 v3 release as described [18]; the remaining had been sequenced as part of the 1000 Genomes Phase1 v3 release (low-coverage whole genome and high-coverage exome sequencing data). Using VCFtools, we created PLINK binary ped/map files for each of the 38 intervals and kept only biallelic SNPs with a MAF >0.05, imputation call-rates ≥ 0.7, Hardy–Weinberg equilibrium *P*-value >1x10^−04^ and SNPs with no missing genotypes, we also only included SNPs that we had primary trait association summary statistics for. These are referred to as the secondary trait genotype files. We then used the JLIM Perl script *fetch*.*refld0*.*EUR*.*pl* to generate the 38 reference LD files from the 373 individuals (the script had been edited to include the extra 95 Finnish individuals).

Cis-eQTL analysis. We created a separate PLINK phenotype file (sample ID, normalized expression residual) for each individual gene, transcript, exon, junction, and intron in *cis* (within +/-500kb) to the 38 lead SLE GWAS SNPs. We only included protein-coding, lincRNA, and antisense genes in our analysis as classified by Ensembl BioMart. Using the chromosome 20 genotype VCF file of the 373 European individuals (E-GEUV-1), we conducted principle component analysis (PCA) and generated an identity-by-state matrix using the Bioconductor package SNPRelate (S9 Fig) [41]. Based on these results, we decided to include the first three principle components and the binary imputation status (as 41 individuals had been genotyped on the Omni 2.5M SNP array were imputed to the Phase 1 v3 release) of the European individuals (derived from Phase1 and Phase2 1000Genomes releases) in the *cis*-eQTL analysis so to minimize biases derived from population structure and imputation status.

We used PLINK to perform *cis*-eQTL analysis using the ‘*—linear*’ function, including the above covariates, for each expression unit (phenotype file) in *cis* to the 38 loci (secondary trait genotype files). We performed 10,000 permutations per regression and saved the output of each permutation procedure. In *cis* to the 38 SLE SNPs were: 439 genes, 1,448 transcripts (originating from 456 genes), 3,045 exons (400 genes), 2,886 junctions (332 genes), and 1,855 introns (443 genes).

Joint likelihood mapping (JLIM) and multiple testing correction. Per RNA-Seq quantification type, a JLIM configuration file was created using the *jlim_gencfg*.*sh* script and JLIM then run using *run_jlim*.*sh*–setting the *r*^2^ resolution limit to 0.8. We merged the configuration files and output files to create the final results table which included the primary and secondary trait association *P*-value, the JLIM statistic, and the JLIM *P*-value by permutation. Multiple testing was corrected for on the JLIM *P*-values per RNA-Seq quantification type using a false discovery rate (FDR) as applied by the authors of JLIM. A JLIM *P*-value <10^−04^ means that the JLIM statistic is more extreme than the permutation (10,000). We classified causal *cis*-eQTLs as SLE associated variants that share a single causal variant with a *cis*-eQTL based on the following: if there existed a nominal *cis*-eQTL (*P*<0.01) with at least one SNP within 100kb of the SNP most associated with disease, the transcription start site of the expression target was located within +/-500kb of that SNP, and the FDR adjusted JLIM *P*-value of the association passed the 5% threshold. Candidate genes modulated by the causal *cis*-eQTL.

### Functional annotation of SLE associated genes from cis-eQTL analysis

Using publically available resources, we systematically annotated the twelve SLE associated genes that were classified as being modulated by causal *cis*-eQTLs. The expression profiles at RNA-level across multiple cell and tissue types were interrogated in GTEx [42] and the Human Protein Atlas [43]—with the top three cell/tissue types documented per gene. We noted using Online Mendelian Inheritance in Man [44] any gene-phenotype relationships by caused by allelic variants and any immune-related phenotypes of animal models. Protein-protein interactions of candidate genes were taken from the BioPlex v2.0 interaction network (conducted in HEK293T cells) [45]. Using the ImmunoBase resource (https://www.immunobase.org/), we looked up each gene and noted if the gene had been prioritized as the ‘candidate gene’ within the susceptibility locus per publication. Finally, we counted the number publications from PubMed found using the keywords ‘gene name AND SLE’.

### Associated SNPs from twenty autoimmune diseases

Autoimmune associated SNPs were taken from the ImmunoBase resource (www.immunobase.org). This resource comprises summary case-control association statistics from twenty diseases: twelve originally targeted by the ImmunoChip consortium (ankylosing spondylitis, autoimmune thyroid disease, celiac disease, Crohn's disease, juvenile idiopathic arthritis, multiple sclerosis, primary biliary cirrhosis, psoriasis, rheumatoid arthritis, systemic lupus erythematosus, type 1 diabetes, ulcerative colitis), and eight others (alopecia areata, inflammatory bowel disease, IgE and allergic sensitization, narcolepsy, primary sclerosing cholangitis, Sjogren syndrome, systemic scleroderma, vitiligo).

The curated studies and their corresponding references used in this analysis are presented in S6 Table. For each disease, we took the lead SNPs which were defined as a genome-wide significant SNP with the lowest reported *P*-value in a locus. Associations on the X-chromosome and within the MHC and SNPs with minor allele frequency < 5% were omitted from analysis, leaving 752 associated SNPs. We pruned these loci using the ‘*—indep-pairwise*’ function of PLINK 1.9 with a window size of 100kb and an *r*^2^ threshold of 0.8, to create an independent subset of 560 loci.

### Integrative cis-eQTL analysis of twenty autoimmune diseases with RNA-Seq

An overview of the integration pipeline using the twenty autoimmune diseases against the Geuvadis RNA-Seq cohort in 373 European LCLs is depicted in S10 Fig. Genotype data of the 373 individuals were transformed and quality controlled as previously described in the above methods sections (biallelic SNPs kept with a MAF >0.05, imputation call-rates ≥ 0.7, Hardy–Weinberg equilibrium *P*-value >1x10^−04^).

We opted to use the Regulatory Trait Concordance (RTC) method to assess the likelihood of a shared causal variant between the disease association and the *cis*-eQTL signal [46]. This method requires full genotype-level data for the expression trait but only the marker identifier for the lead SNP of the disease association trait. SNPs within the 560 associated loci for the expression trait were firstly classified according to their position in relation to recombination hotspots (based on genome-wide estimates of hotspot intervals) [47]. Normalized gene expression residuals (PEER factor normalized RPKM) for each quantification type were transformed to standard normal and the first three principle components used as covariates in the *cis*-eQTL model as well as the binary imputation status (as previously described above). All *cis*-eQTL association testing was performed using a liner regression model in R. *Cis*-eQTL mapping was performed for the lead SNP and all SNPs within the hotspot recombination interval against protein-coding, lincRNA, and antisense expression elements (genes, transcripts, exons etc.) within +/-500kb of the lead SNP. In *cis* to the 560 loci were: 7,633 genes, 27,257 transcripts (originating from 7,310 genes), 52,651 exons (5,435 genes), 48,627 junctions (4,237 genes), 34,946 introns (6,233 genes).

For each *cis*-eQTL association, the residuals from the linear-regression of the best *cis*-asQTL (lowest association *P*-value within the hotspot interval) were extracted. Linear regression was then performed using all SNPs within the defined hotspot interval against these residuals. The RTC score was then calculated as (*N*_*SNPs*_*—Rank*_*GWAS SNP*_ */ N*_*SNPs*_). Where *N*_*SNPs*_ is the total number of SNPs in the recombination hotspot interval, and *Rank*_*GWAS SNP*_ is the rank of the GWAS SNP association *P*-value against all other SNPs in the interval from the liner association against the residuals of the best *cis*-eQTL.

We rigorously adjusted for multiple testing of *cis*-eQTL *P*-values using a Bonferroni correction per quantification type (corrected for number of genes, isoforms, exons, junctions, and introns tested) and per disease–as we wanted to keep our analysis as close to the authors of JLIM who themselves also adjusted per cell type and per disease. We stringently defined causal *cis*-eQTLs as associations with expression *P*_BF_ < 0.05 and an RTC score ≥ 0.95. Candidate genes are modulated by the *cis*-eQTL.

### Mixed-effects model test of heterogeneity

Expression of gene elements (for example exons) within a gene are naturally correlated, as are expression data from the same individual. We therefore applied a linear mixed-effects model approach within each RNA-Seq quantification type to test for heterogeneity in *cis*-eQTL signal strength of causal associations. We firstly fitted a systematic gene-model containing a SNP allele dosage main effect (encoded 0, 1, 2) and two random effects terms indexing each individual (1|Sample) and each expression element found within the same gene (1|Target). We then fitted a heterogeneous gene-model containing the same terms plus a set of fixed-effect SNP dosage * expression element interaction terms. Both models were fitted via restricted maximum likelihood (REML = FALSE) using the lmer() function of the lme4 R package. A likelihood ratio test was used to determine significance (anova). *P*-values were corrected for multiple testing using a Bonferroni correction, correcting for all tests (n = 230) across all quantification types. *P*_BF_ < 0.05 was deemed significant for the heterogeneous model.

### Functional enrichment of causal cis-eQTLs in chromatin regulatory elements

To test for enrichment of causal *cis*-eQTL associations in chromatin regulatory elements we implemented the Genomic Annotation Shifter (GoShifter) package [25]. Chromatin regulatory elements were divided into two categories: chromatin state segmentation and histone marks. The genomic coordinates of the fifteen predicted chromatin state segmentations (active promoter, strong enhancer, insulator etc.) for LCLs (in the GM12878 cell-line) were downloaded from the UCSC Table browser (track name: wgEncodeBroadHmmGm12878HMM). Histone marks and DNase hypersensitivity sites were obtained from the NIH Roadmap Epigenomics Project for LCLs (GM12878) in NarrowPeak format. Sites were filtered for genome-wide significance using an FDR threshold of 0.01 and peak widths harmonised to 200bp in length centred on the peak summit (as used in the GoShifter publication).

We obtained all SNPs in strong LD (*r*^*2*^ > 0.8) with the causal *cis*-eQTLs by using the *getLD*.*sh* script from GoShifter (interrogating the 1000Genomes Project for Phase3 Europeans). Per quantification type, we then calculated the proportion of loci in which at least one SNP in LD overlapped a chromatin regulatory element (conducted one at a time per chromatin mark). The coordinates of the chromatin marks were then randomly shifted, whilst retaining the positions of the SNPs, and frequency of overlap re-calculated. This was carried out over 1,000 permutations to draw the null distribution. The *P*-value was calculated as the proportion of iterations for which the number of overlapping loci was equal to or greater than that for the tested SNPs (*P* < 0.05 used as significance threshold).

### Genome-wide cis-eQTL analysis

Genome-wide *cis*-eQTL analysis was performed using the normalized expression residuals for each quantification type, four population principle components, and quality controlled SNP genotype data of the 373 European individuals as already described. *Cis*-eQTL association analysis was performed using the MatrixeQTL R package fitting the linear-model function for all SNPs within +/-500kb of protein-coding expression targets [48]. The total number of SNPs, genes, targets, and SNP-gene targets tested are documented in S8 Table and S11 Fig. The issue of multiple testing was addressed by calculating a False Discovery Rate for each SNP-target pair per quantification type and thresholding associations below 5%.

### Data visualisation and online resource

R version 3.3.1 and ggplot2 was used to create heatmaps, box-plots, and correlation plots. Genes were plotted in UCSC Genome Browser [49] and regional association plots in LocusZoom [50]. To access the online results table, visit www.insidegen.com and follow the link ‘Lupus’ then ‘data for scientists’. The table is found under title ‘Expression data associated with different autoimmune diseases’.

## Supporting information

S1 Table SLE GWAS in persons of European Descent (38 loci taken forward for cis-eQTL analysis). Associations taken from the Bentham & Morris et al 2015 SLE GWAS in persons of European descent (4,036 cases and 6,969 controls). See original publication for full details. Only non-MHC, MAF > 5%, non-conditional associations were kept for eQTL analysis (leaving these 38 loci in total).
(PDF)

S2 Table SLE associated cis-eQTL associations deemed to be causal as defined by the JLIM pipeline. The lead SNPs from the Bentham and Morris et al 2015 GWAS in persons of European descent were functionally annotated by cis-eQTL analysis in the Geuvadis RNA-Seq cohort in lymphoblastoid cell lines using RNA-Seq quantification profiled at five resolutions (gene, transcript, exon, junction, and intron). Only SNPs reaching genome-wide significance, not conditional peaks, outside of the major histocompatibility complex loci, and with minor allele frequency > 5% were included leaving 38 SLE lead SNPs in total. All SLE loci were densely imputed to the 1000 Genomes Phase 3 Imputation Panel as described in methods. All 38 loci (+/-100kb of each lead SNP) comprised a nominally significant cis-eQTL (P<0.01) for at least one gene within +/-500kb of the lead SNP at each resolution of RNA-Seq. Evidence of a single shared causal variant at each locus was assessed using the Joint Likelihood Mapping (JLIM) algorithm as described in methods. Causal cis-eQTLs are defined where the disease association is consistent with a single shared effect for at least one cis-eQTL (P<0.01 and JLIM FDR adjusted P<0.05). Level: RNA-Seq quantification type, Target: The expression target–defined by chromosome and genomic coordinate (hg19). IndexRs: the rs ID of the SLE GWAS SNP, idxP: The P-value of SLE association derived from the GWAS, idx2bp: the SLE association P-value of the most associated SNP within +/-100kb of the lead SNP (may be different due to the reporting of the most likely causal SNP from the original GWAS, idx2P: the P-value of the most associated SNP, minP2: The cis-eQTL P-value of the most associated SNP with the expression target, STAT: The JLIM statistic, p: The JLIM P-value, FDR: The false discovery rate adjusted P-value.
(PDF)

S3 Table All SLE associated cis-eQTL associations by the JLIM pipeline–causal and non-causal associations. SLE associated cis-eQTL associations deemed to be causal as defined by the JLIM pipeline (this is the output from JLIM). The lead SNPs from the Bentham and Morris et al 2015 GWAS in persons of European descent were functionally annotated by cis-eQTL analysis in the Geuvadis RNA-Seq cohort in lymphoblastoid cell lines using RNA-Seq quantification profiled at five resolutions (gene, transcript, exon, junction, and intron). Only SNPs reaching genome-wide significance, not conditional peaks, outside of the major histocompatibility complex loci, and with minor allele frequency > 5% were included leaving 38 SLE lead SNPs in total. All SLE loci were densely imputed to the 1000 Genomes Phase 3 Imputation Panel as described in methods. All 38 loci (+/-100kb of each lead SNP) comprised a nominally significant cis-eQTL (P<0.01) for at least one gene within +/-500kb of the lead SNP at each resolution of RNA-Seq. Evidence of a single shared causal variant at each locus was assessed using the Joint Likelihood Mapping (JLIM) algorithm as described in methods. Causal cis-eQTLs are defined where the disease association is consistent with a single shared effect for at least one cis-eQTL (P<0.01 and JLIM FDR adjusted P<0.05).
(XLSX)

S4 Table Functional annotation of SLE candidate genes detected by cis-eQTL analysis using RNA-Seq. Using publically available resources, we systematically annotated the twelve SLE associated genes that were classified as being modulated by causal cis-eQTLs. The expression profiles at RNA-level across multiple cell and tissue types were interrogated in GTEx and the Human Protein Atlas—with the top three cell/tissue types documented per gene. We noted using Online Mendelian Inheritance in Man any gene-phenotype relationships by caused by allelic variants and any immune-related phenotypes of animal models. Protein-protein interactions of candidate genes were taken from the BioPlex v2.0 interaction network. Using the ImmunoBase resource, we looked up each gene and noted if the gene had been prioritized as the ‘candidate gene’ within the susceptibility locus per disease. Finally, we counted the number publications from PubMed found using the keywords ‘gene name AND SLE’. We have highlighted in bold and underlined the candidate genes that we are sceptical about due to the lack of functional support and the known functional consequences at these loci. Although the cis-eQTLs for these genes are classified as statistically having the same underlying causal variant as the disease association–our functional genomic data do not robustly support these genes as likely to be involved in pathogenesis. It is possible that these effects are secondary to the pathogenic effect i.e. carried as a passenger on the same functional haplotype but do not contribute to autoimmunity.
(PDF)

S5 Table Number of expression elements that are deemed to have a causal association with the SLE risk SNP. This table shows how may expression elements (i.e. number of exons, junctions, introns etc.) that are deemed to have a causal cis-eQTL association with the SLE associated SNP (taken from Table 3). A dashed line indicates that no causal association exists at that particular quantification type. For example, rs3768792 is a causal cis-eQTL for *IKZF2* at both exon- and intron-level. Out of the five exons of *IKZF2* that are included in the cis-eQTL analysis (some may have been dropped form analysis due to low expression etc.), only one shows a causal cis-eQTL association with rs3768792. The same is true for one of the fifteen introns of *IKZF2*. For *BANK1*, we were able to resolve to two exons.
(PDF)

S6 Table Curated studies of the ImmunoBase Resource. Associations refers to the number of unique susceptibility loci identified per disease across studies of the same disease. Studies consist of Genome-wide Association Studies (GWAS), Immunochip studies, and meta-analyses/replication. Date refers to date of publication.
(PDF)

S7 Table Results of linear mixed-model to test for heterogeneity in cis-eQTL signal across different RNA-Seq profiling types. Results of mixed-model approach to test for heterogeneity in cis-eQTL signal across different RNA-Seq profiling types. Associations are classified as having a causal cis-eQTL at gene-level or not and are broken down by RNA-Seq profiling type. The number of causal targets refers to the number of transcripts, exons, junctions, or introns are deemed to be causal at the corresponding profiling type. Chiseq is the Chi-squared statistic following a likelihood ratio test between the systematic model and the model of heterogeneity. Systematic model: SNP allele dosage main effect and two random effects terms indexing each individual in the data set and each target in the gene. Heterogeneous model: containing the same terms plus a set of fixed-effect SNP dosage × target ID interaction terms. DF: degrees of freedom. P-value: P-value of likelihood ratio test for heterogeneous model. All P-values were corrected by Bonferroni multiple testing correction. Associations with PBF < 0.05 were deemed to fit the heterogeneous model. Table is sorted by P-value.
(XLSX)

S8 Table Summary results of genome-wide cis-eQTL analysis. The results of this table are depicted in S11 Fig. A genome-wide cis-eQTL analysis was performed as described in methods for all common SNPs (MAF > 5%) against gene quantifications profiled at gene-, transcript-, exon-, junction-, and intron-level. ‘TOTAL’ refers to the total number of elements tested in a genome-wide setting. The number of SNPs is different per quantification type as the analysis is run in cis, meaning only expression elements within +/-500kb of each SNP are considered; therefore, if there is no expression element within this distance, the SNP is not included in the analysis. ‘Genes’ refers to the number of distinct (unique) genes tested against, and ‘targets’ refers to the number of individual genes, transcripts, exons, junctions, and introns tested against measured using the corresponding profiling type. ‘SIGNIFICANT’ refers to the number of SNPs, genes etc. that pass a genome-wide false discovery rate (FDR) multiple testing threshold of 5%. ‘PERCENTAGE SIGNIFICANT’ refers to the percentage of SNPs, genes etc. that are significant as a percentage of the total tested.
(PDF)

S9 Table Candidate causal cis-eQTLs of autoimmune loci with genome-wide FDR q-value. All candidate causal cis-eQTLs detected in the GWAS-eQTL integration passed a genome-wide significance FDR threshold of 5%.
(XLSX)

S1 Fig Overview of the five quantification types used to estimate gene expression using RNA-Seq. The following text (*) has been lifted from the supplementary material from Lappalainen et al 2013 regarding how expression using RNA-Seq was estimated across the five RNA-Seq quantification types (gene-, transcript-, exon-, junction-, and intron-level; S1A Fig). Further information is available on the Geuvadis wiki page: http://geuvadiswiki.crg.es/index.php/Main_Page and on the Flux Capacitor webpage for the Geuvadis Project: http://sammeth.net/confluence/display/FLUX/Geuvadis+Quantifications. In this work, normalised RNA-Seq expression data of 373 lymphoblastoid cell lines from four European sub-populations (CEU, GBR, FIN, TSI) of the 1000Genomes Project (Geuvadis) were obtained from EBI ArrayExpress (E-GEUV-1). Quantification was performed at gene-, transcript-, exon-, junction-, and intron-level as described below. Quantifications were corrected for sequencing depth and gene length (RPKM). Only expression elements quantified in > 50% of individuals were kept and Probabilistic Estimation of Expression Residuals (PEER) was used to remove technical variation and expression residuals transformed to a standard normal distribution. (*) Quantifications of transcripts and splice junctions by the Flux Capacitor approach are based on the annotation‐mapped genomic mappings considering transcript structures of the GENCODE transcriptome annotation: mappings of read pairs that were completely included within the annotated exon boundaries and paired in the expected orientation have been considered. Reads belonging to single transcripts were predicted by deconvolution according to observations of paired reads mapping across all exonic segments of a locus. Gene quantifications were calculated as the sum of all transcript RPKMs per gene. Annotated splice junctions were quantified using split read information, counting the number of reads supporting a given junction. Exon quantifications were calculated for protein‐coding and lincRNA transcripts. All overlapping exons of a gene were merged into metaGexons with identifier of type ENSG000001.1_exon.start.pos_exon.end.pos. Read counts over these elements were calculated without using information of read pairing, except for excluding reads where the pairs map to two different genes. We counted a read in an exon if either its start or end coordinate overlapped an exon. For split reads, we counted the exon overlap of each split fragment, and added counts per read as 1/ (number of overlapping exons per gene). The following is modified from the Flux Capacitor webpage. In S1B Fig, the red box marks the all-intronic intersection of the two displayed introns. Read coverage of this region is expressed by the fraction of the intron that is covered by reads 10-bin resolution as a default). Intron parts outside of the red box are not considered for quantifying this feature, under the hypothesis that reads falling there could be assigned to the superimposed alternative exon boundaries. So, the coverage value reported for an intron is the fraction of the red box covered by reads (default resolution 0.1).
(TIF)

S2 Fig Distribution of joint likelihood P-values across RNA-Seq quantification types with 38 SLE GWAS loci. (TIF)

S3 Fig Specificity of cis-eQTLs and candidate genes identified by joint likelihood mapping using SLE GWAS across the five RNA-Seq quantification types. (TIF)

S4 Fig Regional association plots (+/-250kb) of SLE GWAS in Europeans. Showing the nine loci that are causal *cis*-eQTLs and candidate genes from JLIM analysis. The full results of this analysis are in Table 3 of the manuscript and the summary results from the GWAS as provided in S1 Table. Candidate genes are highlighted in red.
(TIF)

S5 Fig SLE associated SNP rs3768792 is a causal cis-eQTL for *IKZF2* for a single exon and a single intron. Full results of the causal cis-eQTL associations are found in S2 Table. This figure shows how cis-eQTL analysis can be used to resolve to a single expression element targeted by a disease associated SNP. (A) The genomic coordinates and isoform structure of SLE candidate gene *IKZF2* detected by cis-eQTL analysis using RNA-Seq at exon-level and intron-level. The transcription start site of *IKZF2* is on the right-hand side. In the red box is the single exon and single intron modulated by causal cis-eQTL rs3768792 –these are shown in the black boxes. (B) A zoomed in view of the red box showing the exon, coordinates: chr2: 213886368–213886444 and intron, coordinates: chr2: 213881768–213886189. The track above shows the transcription levels assayed by RNA-Seq in LCLs (GM12878 cell line) from the ENCODE project–the affected intron is clearly transcribed. (C) The SLE risk allele rs3768792 [A] leads to increased expression of both the depicted exon and the intron of *IKZF2*. The cis-eQTL association P-value and JLIM P-value are shown.
(TIF)

S6 Fig SLE associated SNP rs7444 is a causal cis-eQTL for *UBE2L3* for a single transcript and a single exon. Full results of the causal cis-eQTL associations are found in S2 Table. This figure shows how cis-eQTL analysis can be used to resolve to a single expression element targeted by a disease associated SNP. (A) The genomic coordinates and isoform structure of SLE candidate gene *UBE2L3* detected by cis-eQTL analysis using RNA-Seq at exon-level and transcript-level. The transcription start site of *UBE2L3* is on the left-hand side. The track above shows the transcription levels assayed by RNA-Seq in LCLs (GM12878 cell line) from the ENCODE project. The SLE risk allele rs7444 [T] leads to increased expression of both the depicted transcript (ENST00000458578) and the exon of *UBE2L3*. The cis-eQTL association P-value and JLIM P-value are shown.
(TIF)

S7 Fig SLE associated SNP rs9872955 is a causal cis-eQTL for *LYST* for a single junction. Full results of the causal cis-eQTL associations are found in S2 Table. This figure shows how cis-eQTL analysis can be used to resolve to a single expression element targeted by a disease associated SNP. Top panel—the genomic coordinates and isoform structure of SLE candidate gene *LYST* detected by cis-eQTL analysis using RNA-Seq at junction-level. The transcription start site of *LYST* is on the right-hand side. The track above shows the transcription levels assayed by RNA-Seq in LCLs (GM12878 cell line) from the ENCODE project. The SLE risk allele rs9872955 [C] leads to decreased expression of the depicted junction (chr1: 235915471–235916344). The cis-eQTL association P-value and JLIM P-value are shown.
(TIF)

S8 Fig Exon and intron numbers for *TYK2*. The transcription start site is on the right of the diagram. This corresponds to Fig 2.
(TIF)

S9 Fig Processing of genotype data and principle component analysis. Genotype data in VCF format of 1000Genomes individuals were downloaded from E-GEUV1 (ArrayExpress). Insertion-deletion sites were removed, and bi-allelic SNPs kept only. SNPs with HWE < 0.0001 were removed and the VCF converted to 0,1,2 format using PLINK. Principle component analysis was performed on genotype data using the R package SNPRelate on chromosome 20. The first 3 components were included in the eQTL regression model as well as the binary imputation status (see methods).
(TIF)

S10 Fig Overview of integrative cis-eQTL analysis pipeline using 20 autoimmune diseases. The 752-autoimmune disease associated SNPs per disease are documented in S1 Table and were LD pruned to 560 independent loci (see methods). Genotypes of 1000Genomes individuals were quality controlled and subset to regions of recombination hotspots. If the lead GWAS SNP was found between a recombination hotspot, then all SNPs were between the recombination hotspot intervals were used in the Regulatory Trait Concordance (RTC) analysis. If the lead GWAS SNP was found within a recombination hotspot itself, then all SNPs before or after the summit (including the between summit SNPs) were used in the RTC (upper-interval and lower-interval hotspot respectively). Normalized RNA-Seq expression data at gene-, isoform-, exon-, junction-, and intron-level were obtained for the 1000Genomes individuals of the Geuvadis cohort in lymphoblastoid cell lines. Disease associated SNPs with statistically significant association with gene expression (PBF < 0.05) and an RTC score > 0.95 were defined as causal cis-eQTLs.
(TIF)

S11 Fig Summary of genome-wide cis-eQTL analysis at multiple profiling types. This figure corresponds to the data presented in S8 Table. A target is a single gene, transcript, exon, junction, or intron, quantified using the corresponding profiling type. SNPs, genes, targets, and SNP-gene pairs are only counted once (distinct) if multiple SNP-gene pairs exist.
(TIF)

## References

[1] Fever FM. NIH Progress in Autoimmune Diseases Research. in National Institute of Health Publication. 2005; 17–7576.

[2] Parkes M, Cortes A, van Heel DA, Brown MA. Genetic insights into common pathways and complex relationships among immune-mediated diseases. Nat Rev Genet. Nature Publishing Group; 2013;14: 661–73. doi: 10.1038/nrg3502 23917628 10.1038/nrg3502

[3] Hindorff LA, Sethupathy P, Junkins HA, Ramos EM, Mehta JP, Collins FS, et al Potential etiologic and functional implications of genome-wide association loci for human diseases and traits. Proc Natl Acad Sci U S A. 2009;106: 9362–9367. doi: 10.1073/pnas.0903103106 19474294 10.1073/pnas.0903103106 PMC2687147

[4] Westra H-J, Franke L. From genome to function by studying eQTLs. Biochim Biophys Acta. Elsevier B.V.; 2014;1842: 1896–1902. doi: 10.1016/j.bbadis.2014.04.024 24798236 10.1016/j.bbadis.2014.04.024

[5] Klionsky DJ. Crohn’s disease, autophagy, and the Paneth cell. N Engl J Med. 2009;360: 1785–1786. doi: 10.1056/NEJMcibr0810347 19369659 10.1056/NEJMcibr0810347 PMC2832908

[6] Hu X, Kim H, Raj T, Brennan PJ, Trynka G, Teslovich N, et al Regulation of Gene Expression in Autoimmune Disease Loci and the Genetic Basis of Proliferation in CD4+ Effector Memory T Cells. PLoS Genet. 2014;10 doi: 10.1371/journal.pgen.1004404 24968232 10.1371/journal.pgen.1004404 PMC4072514

[7] Bentham J, Morris DL, Cunninghame Graham DS, Pinder CL, Tombleson P, Behrens TW, et al Genetic association analyses implicate aberrant regulation of innate and adaptive immunity genes in the pathogenesis of systemic lupus erythematosus. Nat Genet. Nature Publishing Group; 2015;47: 1457–1464. doi: 10.1038/ng.3434 26502338 10.1038/ng.3434 PMC4668589

[8] Fairfax BP, Knight JC. Genetics of gene expression in immunity to infection. Curr Opin Immunol. Elsevier Ltd; 2014;30: 63–71. doi: 10.1016/j.coi.2014.07.001 25078545 10.1016/j.coi.2014.07.001 PMC4426291

[9] Chun S, Casparino A, Patsopoulos NA, Croteau-chonka DC, Raby BA, Jager PL De, et al Limited statistical evidence for shared genetic effects of eQTLs and autoimmune-disease-associated loci in three major immune-cell types. NatGenet. 2017; doi: 10.1038/ng.3795 28218759 10.1038/ng.3795 PMC5374036

[10] Odhams CA, Cortini A, Chen L, Roberts AL, Viñuela A, Buil A, et al Mapping eQTLs with RNA-seq reveals novel susceptibility genes, non-coding RNAs and alternative-splicing events in systemic lupus erythematosus. Hum Mol Genet. 2017;26: ddw417 doi: 10.1093/hmg/ddw417 28062664 10.1093/hmg/ddw417 PMC5409091

[11] Trapnell C, Roberts A, Goff L, Pertea G, Kim D, Kelley DR, et al Differential gene and transcript expression analysis of RNA-seq experiments with TopHat and Cufflinks. Nat Protoc. 2012;7: 562–78. doi: 10.1038/nprot.2012.016 22383036 10.1038/nprot.2012.016 PMC3334321

[12] Li B, Dewey CN. RSEM: accurate transcript quantification from RNA-Seq data with or without a reference genome. BMC Bioinformatics. 2011;12: 323 doi: 10.1186/1471-2105-12-323 21816040 10.1186/1471-2105-12-323 PMC3163565

[13] Schuierer S, Roma G. The exon quantification pipeline (EQP): a comprehensive approach to the quantification of gene, exon and junction expression from RNA-seq data. Nucleic Acids Res. 2016; gkw538 doi: 10.1093/nar/gkw538 27302131 10.1093/nar/gkw538 PMC5027495

[14] Anders S, Pyl PT, Huber W. HTSeq-A Python framework to work with high-throughput sequencing data. Bioinformatics. 2015;31: 166–169. doi: 10.1093/bioinformatics/btu638 25260700 10.1093/bioinformatics/btu638 PMC4287950

[15] Anders S, Reyes A, Huber W. Detecting differential usage of exons from RNA-seq-npre20126837-2.pdf. Genome Res. 2012;12: 1088–9051. doi: 10.1101/gr.133744.111 10.1101/gr.133744.111 PMC3460195 22722343

[16] Montgomery SB, Sammeth M, Gutierrez-Arcelus M, Lach RP, Ingle C, Nisbett J, et al Transcriptome genetics using second generation sequencing in a Caucasian population. Nature. Nature Publishing Group; 2010;464: 773–777. doi: 10.1038/nature08903 20220756 10.1038/nature08903 PMC3836232

[17] Liao Y, Smyth GK, Shi W. FeatureCounts: An efficient general purpose program for assigning sequence reads to genomic features. Bioinformatics. 2014;30: 923–930. doi: 10.1093/bioinformatics/btt656 24227677 10.1093/bioinformatics/btt656

[18] Lappalainen T, Sammeth M, Friedländer MR, ‘t Hoen P a C, Monlong J, Rivas M a, et al Transcriptome and genome sequencing uncovers functional variation in humans. Nature. 2013;501: 506–11. doi: 10.1038/nature12531 24037378 10.1038/nature12531 PMC3918453

[19] Battle A, Mostafavi S, Zhu X, Potash JB, Weissman MM, McCormick C, et al Characterizing the genetic basis of transcriptome diversity through RNA-sequencing of 922 individuals. Genome Res. 2014;24: 14–24. doi: 10.1101/gr.155192.113 24092820 10.1101/gr.155192.113 PMC3875855

[20] Zhao S, Xi L, Zhang B. Union exon based approach for RNA-seq gene quantification: To be or not to be? PLoS One. 2015;10: e0141910 doi: 10.1371/journal.pone.0141910 26559532 10.1371/journal.pone.0141910 PMC4641603

[21] Li YI, Geijn B Van De, Raj A, Knowles D a, Petti A a, Golan D, et al RNA splicing is a primary link between genetic variation and disease. Science. 2016;352 doi: 10.1126/science.aad9417 27126046 10.1126/science.aad9417 PMC5182069

[22] Laiho A, Elo LL. A note on an exon-based strategy to identify differentially expressed genes in RNA-seq experiments. PLoS One. 2014;9: 1–12. doi: 10.1371/journal.pone.0115964 25541961 10.1371/journal.pone.0115964 PMC4277429

[23] Gaidatzis D, Burger L, Florescu M, Stadler MB. Analysis of intronic and exonic reads in RNA-seq data characterizes transcriptional and post-transcriptional regulation. Nat Biotech. Nature Publishing Group; 2015;33: 722–729. doi: 10.1038/nbt.3269 26098447 10.1038/nbt.3269

[24] Mortazavi A, Williams BA, McCue K, Schaeffer L, Wold B. Mapping and quantifying mammalian transcriptomes by RNA-Seq. Nat Methods. 2008;5: 621–628. doi: 10.1038/nmeth.1226 18516045 10.1038/nmeth.1226 PMC13303166

[25] Trynka G, Westra HJ, Slowikowski K, Hu X, Xu H, Stranger BE, et al Disentangling the Effects of Colocalizing Genomic Annotations to Functionally Prioritize Non-coding Variants within Complex-Trait Loci. Am J Hum Genet. The Authors; 2015;97: 139–152. doi: 10.1016/j.ajhg.2015.05.016 26140449 10.1016/j.ajhg.2015.05.016 PMC4572568

[26] Guthridge JM, Lu R, Sun H, Sun C, Wiley GB, Dominguez N, et al Two functional lupus-associated BLK promoter variants control cell-type- and developmental-stage-specific transcription. Am J Hum Genet. 2014;94: 586–598. doi: 10.1016/j.ajhg.2014.03.008 24702955 10.1016/j.ajhg.2014.03.008 PMC3980411

[27] Lewis MJ, Vyse S, Shields AM, Boeltz S, Gordon PA, Spector TD, et al UBE2L3 polymorphism amplifies NF-κB activation and promotes plasma cell development, linking linear ubiquitination to multiple autoimmune diseases. Am J Hum Genet. The Authors; 2015;96: 221–234. doi: 10.1016/j.ajhg.2014.12.024 25640675 10.1016/j.ajhg.2014.12.024 PMC4320258

[28] Kozyrev S V, Abelson A-K, Wojcik J, Zaghlool A, Linga Reddy MVP, Sanchez E, et al Functional variants in the B-cell gene BANK1 are associated with systemic lupus erythematosus. Nat Genet. 2008;40: 211–216. doi: 10.1038/ng.79 18204447 10.1038/ng.79

[29] Getnet D, Grosso JF, Goldberg M V., Harris TJ, Yen HR, Bruno TC, et al A role for the transcription factor Helios in human CD4+CD25+ regulatory T cells. Mol Immunol. Elsevier Ltd; 2010;47: 1595–1600. doi: 10.1016/j.molimm.2010.02.001 20226531 10.1016/j.molimm.2010.02.001 PMC3060613

[30] Kim H, Barnitz RA, Kreslavsky T, Brown FD, Moffett H, Lemieux ME, et al Stable inhibitory activity of regulatory T cells requires the transcription factor Helios. Science. 2015;350: 334–339. doi: 10.1126/science.aad0616 26472910 10.1126/science.aad0616 PMC4627635

[31] Sepulveda FE, Burgess A, Heiligenstein X, Goudin N, Ménager MM, Romao M, et al LYST Controls the Biogenesis of the Endosomal Compartment Required for Secretory Lysosome Function. Traffic. 2015;16: 191–203. doi: 10.1111/tra.12244 25425525 10.1111/tra.12244

[32] Li M, Hou Y, Wang J, Chen X, Shao ZM, Yin XM. Kinetics comparisons of mammalian Atg4 homologues indicate selective preferences toward diverse Atg8 substrates. J Biol Chem. 2011;286: 7327–7338. doi: 10.1074/jbc.M110.199059 21177865 10.1074/jbc.M110.199059 PMC3044989

[33] Prchal-Murphy M, Semper C, Lassnig C, Wallner B, Gausterer C, Teppner-Klymiuk I, et al TYK2 kinase activity is required for functional type I interferon responses in Vivo. PLoS One. 2012;7: 1–12. doi: 10.1371/journal.pone.0039141 22723949 10.1371/journal.pone.0039141 PMC3377589

[34] Farh KK, Marson A, Zhu J, Kleinewietfeld M, Housley WJ, Beik S, et al Genetic and epigenetic fine mapping of causal autoimmune disease variants. Nature. Nature Publishing Group; 2015;518: 337–343. doi: 10.1038/nature13835 25363779 10.1038/nature13835 PMC4336207

[35] Lappalainen T. Functional genomics bridges the gap between quantitative genetics and molecular biology. Genome Res. 2015;25: 1427–1431. doi: 10.1101/gr.190983.115 26430152 10.1101/gr.190983.115 PMC4579327

[36] Albert FW, Kruglyak L. The role of regulatory variation in complex traits and disease. Nat Rev Genet. Nature Publishing Group; 2015;16: 197–212. doi: 10.1038/nrg3891 25707927 10.1038/nrg3891

[37] Trynka G, Sandor C, Han B, Xu H, Stranger BE, Liu XS, et al Chromatin marks identify critical cell types for fine mapping complex trait variants. Nat Genet. Nature Publishing Group; 2013;45: 124–30. doi: 10.1038/ng.2504 23263488 10.1038/ng.2504 PMC3826950

[38] Kim-Hellmuth S, Bechheim M, Puetz B, Mohammadi P, Nedelec Y, Giangreco N, et al Genetic regulatory effects modified by immune activation contribute to autoimmune disease associations. Nat Commun. Springer US; 2017;8: 116376 doi: 10.1101/116376 10.1038/s41467-017-00366-1 PMC5559603 28814792

[39] Ongen H, Dermitzakis ET. Alternative Splicing QTLs in European and African Populations. Am J Hum Genet. The Authors; 2015;97: 567–575. doi: 10.1016/j.ajhg.2015.09.004 26430802 10.1016/j.ajhg.2015.09.004 PMC4596912

[40] Stegle O, Parts L, Durbin R, Winn J. A bayesian framework to account for complex non-genetic factors in gene expression levels greatly increases power in eQTL studies. PLoS Comput Biol. 2010;6: 1–11. doi: 10.1371/journal.pcbi.1000770 20463871 10.1371/journal.pcbi.1000770 PMC2865505

[41] Zheng X, Levine D, Shen J, Gogarten SM, Laurie C, Weir BS. A high-performance computing toolset for relatedness and principal component analysis of SNP data. Bioinformatics. 2012;28: 3326–3328. doi: 10.1093/bioinformatics/bts606 23060615 10.1093/bioinformatics/bts606 PMC3519454

[42] The GTEx Consortium. The Genotype-Tissue Expression (GTEx) project. Nat Genet. 2013;45: 580–585. doi: 10.1038/ng.2653 23715323 10.1038/ng.2653 PMC4010069

[43] Uhlén M, Fagerberg L, Hallström BM, Lindskog C, Oksvold P, Mardinoglu A, et al Proteomics. Tissue-based map of the human proteome. Science. 2015;347: 1260419 doi: 10.1126/science.1260419 25613900 10.1126/science.1260419

[44] Hamosh A, Scott AF, Amberger JS, Bocchini CA, McKusick VA. Online Mendelian Inheritance in Man (OMIM), a knowledgebase of human genes and genetic disorders. Nucleic Acids Res. 2005;33: 514–517. doi: 10.1093/nar/gki033 15608251 10.1093/nar/gki033 PMC539987

[45] Huttlin EL, Ting L, Bruckner RJ, Gebreab F, Gygi MP, Szpyt J, et al The BioPlex Network: A Systematic Exploration of the Human Interactome. Cell. 2015;162: 425–440. doi: 10.1016/j.cell.2015.06.043 26186194 10.1016/j.cell.2015.06.043 PMC4617211

[46] Nica AC, Montgomery SB, Dimas AS, Stranger BE, Beazley C, Barroso I, et al Candidate causal regulatory effects by integration of expression QTLs with complex trait genetic associations. PLoS Genet. 2010;6: e1000895 doi: 10.1371/journal.pgen.1000895 20369022 10.1371/journal.pgen.1000895 PMC2848550

[47] McVean GA. The fine-scale structure of recombination rate variation in the human genome. Science (80-). 2004;304: 581 Available: http://dx.doi.org/10.1126/science.1092500 10.1126/science.1092500 15105499

[48] Shabalin AA. Matrix eQTL: ultra fast eQTL analysis via large matrix operations. Bioinformatics. 2012;28: 1353–1358. doi: 10.1093/bioinformatics/bts163 22492648 10.1093/bioinformatics/bts163 PMC3348564

[49] Kent WJ, Sugnet CW, Furey TS, Roskin KM. The Human Genome Browser at UCSC W. J Med Chem. 2002;19: 1228–31. doi: 10.1101/gr.229102 10.1101/gr.229102 PMC186604 12045153

[50] Pruim RJ, Welch RP, Sanna S, Teslovich TM, Chines PS, Gliedt TP, et al LocusZoom: Regional visualization of genome-wide association scan results. Bioinformatics. 2010;26: 2336–2337. doi: 10.1093/bioinformatics/btq419 20634204 10.1093/bioinformatics/btq419 PMC2935401

