# Supplementary material for: Profiling RNA-Seq at multiple resolutions markedly increases the number of causal eQTLs in autoimmune disease
Source: PLoS Genet. 2017 Oct 23;13(10):e1007071. doi: 10.1371/journal.pgen.1007071 (PMC5695635; doi:10.1371/journal.pgen.1007071)
Supplement: S1 Table — Associations taken from the Bentham & Morris et al 2015 SLE GWAS in persons of European descent (4,036 cases and 6,969 controls). See original publication for full details. Only non-MHC, MAF > 5%, non-conditional associations were kept for eQTL analysis (leaving these 38 loci in total). (PDF) [file pgen.1007071.s001.pdf]

S1 Table

SLE GWAS in persons of European Descent (38 loci taken forward for *cis*-eQTL analysis).

SNP	Chr	hg19 position	Candidate gene	*P*-value	Odds Ratio (95% CI)	Ancestral Allele
rs2476601	chr1	114377568	*PTPN22*	1.10×10^{-28}	1.43 (1.34–1.53)	G
rs1801274	chr1	161479745	*FCGR2A*	1.04×10^{-12}	1.16 (1.11–1.21)	A
rs704840	chr1	173226195	*TNFSF4*	3.12×10^{-19}	1.22 (1.17–1.27)	G
rs17849501	chr1	183542323	*SMG7, NCF2*	3.45×10^{-88}	2.10 (1.95–2.26)	C
rs3024505	chr1	206939904	*IL10*	4.64×10^{-9}	1.17 (1.11–1.24)	G
rs9782955	chr1	236039877	*LYST*	1.25×10^{-9}	1.16 (1.11–1.22)	C
rs2111485	chr2	163110536	*IFIH1*	1.27×10^{-11}	1.15 (1.11–1.20)	A
rs7574865	chr2	191964633	*STAT4*	5.59×10^{-122}	1.73 (1.65–1.81)	T
rs3768792	chr2	213871709	*IKZF2*	1.21×10^{-13}	1.24 (1.17–1.31)	A
rs9311676	chr3	58470351	*ABHD6, PXK*	3.06×10^{-14}	1.17 (1.13–1.22)	C
rs564799	chr3	159728987	*IL12A*	1.54×10^{-9}	1.14 (1.09–1.18)	C
rs10028805	chr4	102737250	*BANK1*	4.31×10^{-17}	1.20 (1.15–1.25)	A
rs7726414	chr5	133431834	*TCF7, SKP1*	4.44×10^{-16}	1.45 (1.32–1.58)	C
rs10036748	chr5	150458146	*TNIP1*	1.27×10^{-45}	1.38 (1.32–1.45)	T
rs2431697	chr5	159879978	*MIR146A*	8.01×10^{-28}	1.26 (1.21–1.31)	C
rs11755393	chr6	34824636	*UHRF1BP1*	7.55×10^{-9}	1.14 (1.09–1.19)	G
rs6568431	chr6	106588806	*PRDM1, ATG5*	5.04×10^{-14}	1.21 (1.15–1.27)	C
rs6920220	chr6	138006504	*TNFAIP3*	1.97×10^{-31}	1.83 (1.65–2.02)	G
rs849142	chr7	28185891	*JAZF1*	8.61×10^{-11}	1.14 (1.10–1.19)	T
rs4917014	chr7	50305863	*IKZF1*	6.39×10^{-14}	1.18 (1.13–1.24)	T
rs10488631	chr7	128594183	*IRF5*	9.37×10^{-110}	1.92 (1.81–2.03)	T
rs2736340	chr8	11343973	*BLK*	6.28×10^{-20}	1.29 (1.22–1.37)	C
rs2663052	chr10	50069395	*WDFY4*	5.25×10^{-9}	1.16 (1.10–1.22)	G
rs4948496	chr10	63805617	*ARID5B*	1.04×10^{-10}	1.14 (1.10–1.19)	C
rs4963128	chr11	589564	*IRF7*	8.81×10^{-10}	1.23 (1.15–1.31)	T
rs2732549	chr11	35088399	*CD44*	1.20×10^{-23}	1.24 (1.19–1.29)	A
rs3794060	chr11	71187679	*DHCR7, NADSYN1*	1.32×10^{-20}	1.23 (1.18–1.29)	C
rs7941765	chr11	128499000	*ETS1, FLI1*	1.35×10^{-10}	1.14 (1.10–1.19)	C
rs10774625	chr12	111910219	*SH2B3*	4.09×10^{-9}	1.13 (1.08–1.18)	G
rs1059312	chr12	129278864	*SLC15A4*	1.48×10^{-13}	1.17 (1.12–1.21)	A
rs4902562	chr14	68731458	*RAD51B*	6.15×10^{-10}	1.14 (1.09–1.19)	G
rs2289583	chr15	75311036	*CSK*	6.22×10^{-15}	1.19 (1.14–1.24)	C
rs9652601	chr16	11174365	*CIITA, SOCS1*	7.42×10^{-17}	1.21 (1.15–1.26)	G
rs11860650	chr16	31326706	*ITGAM*	3.39×10^{-76}	1.71 (1.61–1.81)	C
rs11644034	chr16	85972612	*IRF8*	9.58×10^{-18}	1.25 (1.19–1.32)	G
rs2286672	chr17	4712617	*PLD2*	2.93×10^{-9}	1.25 (1.16–1.35)	C
rs2304256	chr19	10475652	*TYK2*	3.50×10^{-13}	1.24 (1.17–1.31)	C
rs7444	chr22	21976934	*UBE2L3*	1.84×10^{-22}	1.27 (1.21–1.33)	C

Associations taken from the *Bentham & Morris et al 2015* SLE GWAS in persons of European descent (4,036 cases and 6,969 controls). See original publication for full details. Only non-MHC, MAF > 5%, non-conditional associations were kept for eQTL analysis (leaving these 38 loci in total).
