# Supplementary material for: Profiling RNA-Seq at multiple resolutions markedly increases the number of causal eQTLs in autoimmune disease
Source: PLoS Genet. 2017 Oct 23;13(10):e1007071. doi: 10.1371/journal.pgen.1007071 (PMC5695635; doi:10.1371/journal.pgen.1007071)
Supplement: S2 Table — The lead SNPs from the Bentham and Morris et al 2015 GWAS in persons of European descent were functionally annotated by cis-eQTL analysis in the Geuvadis RNA-Seq cohort in lymphoblastoid cell lines using RNA-Seq quantification profiled at five resolutions (gene, transcript, exon, junction, and intron). Only SNPs reaching genome-wide significance, not conditional peaks, outside of the major histocompatibility complex loci, and with minor allele frequency > 5% were included leaving 38 SLE lead SNPs in total. All SLE loci were densely imputed to the 1000 Genomes Phase 3 Imputation Panel as described in methods. All 38 loci (+/-100kb of each lead SNP) comprised a nominally significant cis-eQTL (P<0.01) for at least one gene within +/-500kb of the lead SNP at each resolution of RNA-Seq. Evidence of a single shared causal variant at each locus was assessed using the Joint Likelihood Mapping (JLIM) algorithm as described in methods. Causal cis-eQTLs are defined where the disease association is consistent with a single shared effect for at least one cis-eQTL (P<0.01 and JLIM FDR adjusted P<0.05). Level: RNA-Seq quantification type, Target: The expression target–defined by chromosome and genomic coordinate (hg19). IndexRs: the rs ID of the SLE GWAS SNP, idxP: The P-value of SLE association derived from the GWAS, idx2bp: the SLE association P-value of the most associated SNP within +/-100kb of the lead SNP (may be different due to the reporting of the most likely causal SNP from the original GWAS, idx2P: the P-value of the most associated SNP, minP2: The cis-eQTL P-value of the most associated SNP with the expression target, STAT: The JLIM statistic, p: The JLIM P-value, FDR: The false discovery rate adjusted P-value. (PDF) [file pgen.1007071.s002.pdf]

S2 Table

SLE associated *cis*-eQTL associations deemed to be causal as defined by the JLIM pipeline (this is the output from JLIM).

Level	Target	Name	idxRS	chrom	idxBP	idxP	idx2BP	idx2P	minP2	STAT	p	FDR
Exon	ENSG00000154319.10_11278972_11282145	*FAM167A*	rs2736340	8	11343973	2.14E-16	11339965	1.35E-18	5.10E-46	46.71504784	0	0
Trans	ENST00000284486.4	*FAM167A*	rs2736340	8	11343973	2.14E-16	11339965	1.35E-18	4.43E-45	48.80642864	0	0
Gene	ENSG00000154319.10	*FAM167A*	rs2736340	8	11343973	2.14E-16	11339965	1.35E-18	2.31E-40	42.24461359	0	0
Exon	ENSG00000154319.10_11301540_11302395	*FAM167A*	rs2736340	8	11343973	2.14E-16	11339965	1.35E-18	4.63E-33	26.5489187	0	0
Exon	ENSG00000136573.6_11417842_11418961	*BLK*	rs2736340	8	11343973	2.14E-16	11339965	1.35E-18	1.41E-31	14.15188659	0	0
Exon	ENSG00000136573.6_11407668_11407771	*BLK*	rs2736340	8	11343973	2.14E-16	11339965	1.35E-18	4.41E-30	15.35338136	0	0
Exon	ENSG00000136573.6_11412252_11412398	*BLK*	rs2736340	8	11343973	2.14E-16	11339965	1.35E-18	5.59E-30	8.78106877	0	0
Exon	ENSG00000136573.6_11414167_11414346	*BLK*	rs2736340	8	11343973	2.14E-16	11339965	1.35E-18	1.14E-29	10.05135904	0	0
Exon	ENSG00000136573.6_11420488_11420619	*BLK*	rs2736340	8	11343973	2.14E-16	11339965	1.35E-18	6.02E-29	9.697875438	0	0
Exon	ENSG00000136573.6_11415471_11415547	*BLK*	rs2736340	8	11343973	2.14E-16	11339965	1.35E-18	6.03E-29	10.58889714	0	0
Junction	8_11418961_11420488	*BLK*	rs2736340	8	11343973	2.14E-16	11339965	1.35E-18	7.60E-28	10.480584	0	0
Junction	8_11407771_11412252	*BLK*	rs2736340	8	11343973	2.14E-16	11339965	1.35E-18	1.65E-27	13.92766832	0	0
Junction	8_11412993_11414167	*BLK*	rs2736340	8	11343973	2.14E-16	11339965	1.35E-18	6.43E-27	3.659807232	0	0
Junction	8_11406631_11407668	*BLK*	rs2736340	8	11343973	2.14E-16	11339965	1.35E-18	6.47E-27	14.9105389	0	0
Gene	ENSG00000136573.6	*BLK*	rs2736340	8	11343973	2.14E-16	11339965	1.35E-18	3.29E-26	9.266974735	0	0
Exon	ENSG00000136573.6_11403561_11403612	*BLK*	rs2736340	8	11343973	2.14E-16	11339965	1.35E-18	6.39E-26	10.33062052	0	0
Exon	ENSG00000136573.6_11412841_11412993	*BLK*	rs2736340	8	11343973	2.14E-16	11339965	1.35E-18	1.42E-25	0.935131085	4.00E-04	0.04872
Exon	ENSG00000136573.6_11405541_11405634	*BLK*	rs2736340	8	11343973	2.14E-16	11339965	1.35E-18	3.68E-25	7.036497058	0	0
Junction	8_11400856_11403561	*BLK*	rs2736340	8	11343973	2.14E-16	11339965	1.35E-18	5.18E-25	7.581413942	0	0
Junction	8_11403612_11405541	*BLK*	rs2736340	8	11343973	2.14E-16	11339965	1.35E-18	6.35E-25	7.970289938	0	0
Exon	ENSG00000136573.6_11406533_11406631	*BLK*	rs2736340	8	11343973	2.14E-16	11339965	1.35E-18	7.44E-25	9.678824947	0	0
Junction	8_11414346_11415471	*BLK*	rs2736340	8	11343973	2.14E-16	11339965	1.35E-18	9.84E-25	8.137591462	0	0
Junction	8_11415547_11418811	*BLK*	rs2736340	8	11343973	2.14E-16	11339965	1.35E-18	1.25E-24	8.259710303	0	0
Junction	8_11405634_11406533	*BLK*	rs2736340	8	11343973	2.14E-16	11339965	1.35E-18	1.83E-24	4.015497492	0	0
Exon	ENSG00000136573.6_11400733_11400856	*BLK*	rs2736340	8	11343973	2.14E-16	11339965	1.35E-18	3.04E-24	7.703840438	0	0
Intron	8_11415547_11417842	*BLK*	rs2736340	8	11343973	2.14E-16	11339965	1.35E-18	3.14E-24	15.8312491	0	0
Intron	8_11418961_11420488	*BLK*	rs2736340	8	11343973	2.14E-16	11339965	1.35E-18	6.13E-24	11.91425144	0	0
Junction	8_11412398_11412841	*BLK*	rs2736340	8	11343973	2.14E-16	11339965	1.35E-18	3.08E-23	0.893095144	2.00E-04	0.035825
Junction	8_11282145_11301540	*FAM167A*	rs2736340	8	11343973	2.14E-16	11339965	1.35E-18	1.59E-22	16.81180857	0	0
Junction	8_11352100_11400733	*BLK*	rs2736340	8	11343973	2.14E-16	11339965	1.35E-18	1.33E-21	7.531245764	0	0
Exon	ENSG00000136573.6_11351510_11352100	*BLK*	rs2736340	8	11343973	2.14E-16	11339965	1.35E-18	1.64E-21	10.02643884	0	0
Trans	ENST00000526097.1	*BLK*	rs2736340	8	11343973	2.14E-16	11339965	1.35E-18	1.01E-19	5.784627111	0	0
Junction	8_11420619_11421412	*BLK*	rs2736340	8	11343973	2.14E-16	11339965	1.35E-18	5.06E-19	4.399510844	0	0
Intron	8_11414346_11415471	*BLK*	rs2736340	8	11343973	2.14E-16	11339965	1.35E-18	6.48E-19	5.986960326	0	0
Exon	ENSG00000154319.10_11324135_11324276	*FAM167A*	rs2736340	8	11343973	2.14E-16	11339965	1.35E-18	1.06E-16	13.07603944	0	0
Junction	8_11302317_11324135	*FAM167A*	rs2736340	8	11343973	2.14E-16	11339965	1.35E-18	1.06E-15	9.786471892	0	0
Intron	8_11367400_11400733	*BLK*	rs2736340	8	11343973	2.14E-16	11339965	1.35E-18	2.23E-15	4.423513021	0	0
Intron	8_11304130_11324135	*FAM167A*	rs2736340	8	11343973	2.14E-16	11339965	1.35E-18	7.42E-15	8.121289646	0	0
Exon	ENSG00000185651.10_21975804_21978323	*UBE2L3*	rs7444	22	21976934	2.64E-13	21929566	9.72E-14	9.94E-15	5.887594907	0	0
Trans	ENST00000458578.2	*UBE2L3*	rs7444	22	21976934	2.64E-13	21929566	9.72E-14	5.92E-14	9.106307634	0	0
Intron	8_11407771_11412252	*BLK*	rs2736340	8	11343973	2.14E-16	11339965	1.35E-18	1.06E-13	5.513216363	0	0
Intron	8_11420619_11421412	*BLK*	rs2736340	8	11343973	2.14E-16	11339965	1.35E-18	1.21E-12	3.125500681	0	0
Intron	8_11282145_11301129	*FAM167A*	rs2736340	8	11343973	2.14E-16	11339965	1.35E-18	6.58E-12	1.880658068	0	0

Intron	8_11352100_11366659	*BLK*	rs2736340	8	11343973	2.14E-16	11339965	1.35E-18	7.31E-12	4.169880095	0	0
Trans	ENST00000533828.1	*BLK*	rs2736340	8	11343973	2.14E-16	11339965	1.35E-18	1.52E-11	5.657495318	0	0
Trans	ENST00000526778.1	*BLK*	rs2736340	8	11343973	2.14E-16	11339965	1.35E-18	5.85E-11	3.144335221	1.00E-04	0.0181
Intron	19_10473333_10475290	*TYK2*	rs2304256	19	10475652	1.54E-12	10466123	6.91E-13	2.24E-09	1.9265087	2.00E-04	0.0232
Exon	ENSG00000105397.7_10475527_10475724	*TYK2*	rs2304256	19	10475652	1.54E-12	10466123	6.91E-13	2.58E-09	3.196078064	0	0
Exon	ENSG00000185651.10_21965146_21965332	*UBE2L3*	rs7444	22	21976934	2.64E-13	21929566	9.72E-14	3.04E-09	2.82372309	0	0
Trans	ENST00000525206.1	*BLK*	rs2736340	8	11343973	2.14E-16	11339965	1.35E-18	1.12E-08	4.971535832	0	0
Intron	8_11412993_11414166	*BLK*	rs2736340	8	11343973	2.14E-16	11339965	1.35E-18	3.41E-08	3.739940315	0	0
Trans	ENST00000292779.3	*CCDC116*	rs7444	22	21976934	2.64E-13	21929566	9.72E-14	1.45E-06	1.532076909	3.00E-04	0.048266667
Intron	2_213881768_213886189	*IKZF2*	rs3768792	2	21387170	3.78E-08	21389023	2.04E-08	1.15E-05	0.952921334	2.00E-04	0.0232
Exon	ENSG00000153064.7_102750965_102751363	*BANK1*	rs10028805	4	10273725	4.51E-10	10275609	8.16E-11	1.85E-05	0.57870071	4.00E-04	0.04872
Junction	8_11352100_11366659	*BLK*	rs2736340	8	11343973	2.14E-16	11339965	1.35E-18	2.08E-05	1.70950024	0	0
Gene	ENSG00000161180.5	*CCDC116*	rs7444	22	21976934	2.64E-13	21929566	9.72E-14	2.50E-05	1.159090612	5.00E-04	0.0439
Exon	ENSG00000029725.11_5241314_5241433	*RABEP1*	rs2286672	17	4712617	0.00033644	4697255	2.45E-05	7.41E-05	1.537192432	4.00E-04	0.04872
Exon	ENSG00000186517.8_161019214_161019479	*ARHGAP30*	rs1801274	1	16147974	5.56E-11	16147881	3.59E-12	0.000114	1.019783352	2.00E-04	0.029
Intron	1_114292211_114300321	*PHTF1*	rs2476601	1	11437756	8.38E-13	11437756	8.38E-13	0.000144	3.897248985	1.00E-04	0.012373333
Exon	ENSG00000030419.11_213886368_213886444	*IKZF2*	rs3768792	2	21387170	3.78E-08	21389023	2.04E-08	1.96E-04	1.16986173	3.00E-04	0.039717391
Junction	4_102993557_102994841	*BANK1*	rs10028805	4	10273725	4.51E-10	10275609	8.16E-11	0.000253	0.516404878	2.00E-04	0.035825
Exon	ENSG00000153064.7_102993502_102993557	*BANK1*	rs10028805	4	10273725	4.51E-10	10275609	8.16E-11	0.000348	0.57083328	4.00E-04	0.04872
Junction	19_10657929_10659580	*ATG4D*	rs2304256	19	10475652	1.54E-12	10466123	6.91E-13	0.000385	0.788939164	2.00E-04	0.035825
Exon	ENSG00000161180.5_21990721_21991616	*CCDC116*	rs7444	22	21976934	2.64E-13	21929566	9.72E-14	0.000495	0.590162924	4.00E-04	0.04872
Junction	1_235915471_235916344	*LYST*	rs9782955	1	23603987	0.00079168	23603427	0.00050178	0.001296	1.058660842	2.00E-04	0.035825

The lead SNPs from the *Bentham and Morris et al 2015* GWAS in persons of European descent were functionally annotated by *cis*-eQTL analysis in the Geuvadis RNA-Seq cohort in lymphoblastoid cell lines using RNA-Seq quantification profiled at five resolutions (gene, transcript, exon, junction, and intron). Only SNPs reaching genome-wide significance, not conditional peaks, outside of the major histocompatibility complex loci, and with minor allele frequency > 5% were included leaving 38 SLE lead SNPs in total. All SLE loci were densely imputed to the 1000 Genomes Phase 3 Imputation Panel as described in methods.

All 38 loci (+/-100kb of each lead SNP) comprised a nominally significant *cis*-eQTL ($P<0.01$) for at least one gene within +/-500kb of the lead SNP at each resolution of RNA-Seq. Evidence of a single shared causal variant at each locus was assessed using the Joint Likelihood Mapping (JLIM) algorithm as described in methods.

Causal cis-eQTLs are defined where the disease association is consistent with a single shared effect for at least one *cis*-eQTL ($P<0.01$ and JLIM FDR adjusted $P<0.05$).

Level: RNA-Seq quantification type, Target: The expression target – defined by chromosome and genomic coordinate (hg19). IndexRs: the rs ID of the SLE GWAS SNP, idxP: The *P*-value of SLE association derived from the GWAS, idx2bp: the SLE association *P*-value of the most associated SNP within +/-100kb of the lead SNP (may be different due to the reporting of the most likely causal SNP from the original GWAS, idx2P: the P-value of the most associated SNP, minP2: The *cis*-eQTL *P*-value of the most associated SNP with the expression target, STAT: The JLIM statistic, p: The JLIM *P*-value, FDR: The false discovery rate adjusted *P*-value.
