# Supplementary material for: Profiling RNA-Seq at multiple resolutions markedly increases the number of causal eQTLs in autoimmune disease
Source: PLoS Genet. 2017 Oct 23;13(10):e1007071. doi: 10.1371/journal.pgen.1007071 (PMC5695635; doi:10.1371/journal.pgen.1007071)
Supplement: S4 Table — Using publically available resources, we systematically annotated the twelve SLE associated genes that were classified as being modulated by causal cis-eQTLs. The expression profiles at RNA-level across multiple cell and tissue types were interrogated in GTEx and the Human Protein Atlas—with the top three cell/tissue types documented per gene. We noted using Online Mendelian Inheritance in Man any gene-phenotype relationships by caused by allelic variants and any immune-related phenotypes of animal models. Protein-protein interactions of candidate genes were taken from the BioPlex v2.0 interaction network. Using the ImmunoBase resource, we looked up each gene and noted if the gene had been prioritized as the ‘candidate gene’ within the susceptibility locus per disease. Finally, we counted the number publications from PubMed found using the keywords ‘gene name AND SLE’. We have highlighted in bold and underlined the candidate genes that we are sceptical about due to the lack of functional support and the known functional consequences at these loci. Although the cis-eQTLs for these genes are classified as statistically having the same underlying causal variant as the disease association–our functional genomic data do not robustly support these genes as likely to be involved in pathogenesis. It is possible that these effects are secondary to the pathogenic effect i.e. carried as a passenger on the same functional haplotype but do not contribute to autoimmunity. (PDF) [file pgen.1007071.s004.pdf]

S4 Table

Functional annotation of SLE candidate genes detected by *cis*-eQTL analysis using RNA-Seq.

SNP	Gene	Entrez Description	GTEX	Human Protein Atlas	OMIM Allelic Variants	Animal model	BioPlex Protein Interaction	GWAS associations	PUBMED	Nearest Gene
rs2476601	***PHTF1***	Putative homeodomain transcription factor 1	Cerebellum, Pituitary, LCLs	Parathyroid gland, Bone marrow	NA	NA	GPR156, LYPD3, CAPN5	ATD, CRO, JIA, RA, SLE, T1D, AA, VIT	0	*PTPN22*
rs1801274	***ARHGAP30***	Rho GTPase activating protein 30	Whole blood, Spleen, LCLs	Lymph node, Spleen, Appendix	NA	NA	NA	CRO, UC, IBD, SLE	0	*FCGR2A*
rs9782955	*LYST*	Lysosomal trafficking regulator	Whole blood, Brain, LCLs	Bone marrow, Spleen, Appendix	Chediak-higashi syndrome	Affected mice segregated a seizure phenotype and grey coat colour. Melanosomes of melanocytes associated with hair follicles, the choroid layer of the eye, and neural tube-derived pigment epithelium of the retina were larger and irregularly shaped in affected mice compared with wildtype controls. Secretory vesicles in dermal mast cells of mutant skin were also enlarged.	NA	SLE	1	*LYST*
rs3768792	*IKZF2*	IKAROS family zinc finger 2	LCLs, Whole blood, Oesophagus	Oesophagus, Thyroid gland, Parathyroid gland	NA	Ikzf2 -/- mice had increased numbers of activated T and B cells at 5 months of age, and autoimmune disease was apparent by 6 to 8 months of age. Autoimmunity occurred earlier and was exacerbated by infection with lymphocytic choriomeningitis virus. Ikzf2 -/- Tregs developed an unstable phenotype during inflammatory responses, with reduced Foxp3 expression and increased effector cytokine expression secondary to diminished activation of the Stat5 pathway.	IKZF1, IKZF5	SLE	4	*IKZF2*
rs10028805	*BANK1*	B-cell scaffold protein with ankyrin repeats 1	Spleen, Small intestine, Thyroid	Lymph node, Tonsil, Spleen	Systemic lupus erythematosus	Bank-deficient mice had higher levels of mature B cells and spontaneous GC B cells, particularly in response to T-dependent antigens, as well as higher serum IgG2a. Bank-deficient B cells had an enhanced proliferative response to Cd40.	NA	CRO, SLE, IBD, SSC	30	*BANK1*
rs2736340	*BLK*	BLK proto-oncogene, Src family tyrosine kinase	LCLs, Spleen, Small intestine	Lymph node, Tonsil, Spleen	Maturity-onset diabetes of the young	SYK controls pre-B cell development but does not affect NFKB induction. Saijo et al. (2003) showed that mice triple-deficient in the Src family protein tyrosine kinases (SFKs) Blk, Fyn, and Lyn but not single-deficient or Syk-deficient mice, had impaired Nfkb induction and B-cell development.	SCGB1A1, RNASE3, C20orf177, APBB3, EDEM2, SYCP3, SENP1	RA, SLE, SJO, SSC	52	*BLK*
	FAM167A	Family with sequence similarity 167-member A	Thyroid, Fibroblasts, Pituitary	Thyroid gland, Cerebral gland, Cortex	NA	NA	>40 interactions (49)	RA, SLE	6	*BLK*
rs2286672	***RABEP1***	Rabaptin, RAB GTPase binding effector protein 1	LCLs, Spinal cord, Substanina nigra	Parathyroid gland, Cerebral cortex, Brest	NA	NA	SYNC, KIAA1984, HERC2, RABGEF1, GGA2, C15orf27, EXOC1, NEFL	RA, SLE	0	*PLD2*
rs2304256	*TYK2*	Tyrosine kinase 2	Spleen, Whole blood, LCLs	Spleen, Lymph node, Bone marrow	Immunodeficiency-35	Karaghiosoff et al. (2000) generated Tyk2 -/- mice. In contrast to other Janus kinase family members, where inactivation leads to complete loss of the respective cytokine receptor signal, Tyk2 -/- mice showed reduced responses to Ifna/Ifnb and Il12 and selective deficiency in Stat3 activation in these pathways.	GPR156, CDH8, GMNN, C14orf179, EFNB2, MAS1, CD79A, TNFRSF13B, LRRC46, C10orf47, HAVCR2, RNF13, TMCO3, C16orf71, LGALS3BP, PMEL, NLGN3, TNFRSF19, IL4R	AS, CEL, CRO, JIA, MS, PBC, PSO, RA, SLE, T1D, UC, IBD	20	*TYK2*
	ATG4D	Autophagy related 4D cysteine peptidase	Skin, Colon, Small intestine	Small intestine, Duodenum, Bone marrow	NA	NA	GOPC, P4HA2, IMPDH1	MS, PSO, RA, SLE	0	*TYK2*
rs7444	*UBE2L3*	Ubiquitin conjugating enzyme E2 L3	LCLs, Cortex, Fibroblasts	Cortex, Smooth muscle, Thyroid gland	NA	NA	ARIH1	CEL, CRO, JIA, MS, PSO, RA, SLE, UC, IBD	12	*UBE2L3*
	CCDC116	Coiled-coil domain containing 116	Testis, LCLs	Testis, LCLs	NA	NA	PAPSS1, GNL1	CEL, CRO, JIA, MS, PSO, RA, SLE, UC, IBD	0	*UBE2L3*

Using publically available resources, we systematically annotated the twelve SLE associated genes that were classified as being modulated by causal cis-eQTLs. The expression profiles at RNA-level across multiple cell and tissue types were interrogated in GTEx and the Human Protein Atlas - with the top three cell/tissue types documented per gene. We noted using Online Mendelian Inheritance in Man any gene-phenotype relationships by caused by allelic variants and any immune-related phenotypes of animal models. Protein-protein interactions of candidate genes were taken from the BioPlex v2.0 interaction network. Using the ImmunoBase resource, we looked up each gene and noted if the gene had been prioritized as the 'candidate gene' within the susceptibility locus per disease. Finally, we counted the number publications from PubMed found using the keywords 'gene name AND SLE'.

We have highlighted in **bold** and underlined the candidate genes that we are sceptical about due to the lack of functional support and the known functional consequences at these loci. Although the *cis*-eQTLs for these genes are classified as statistically having the same underlying causal variant as the disease association – our functional genomic data do not robustly support these genes as likely to be involved in pathogenesis. It is possible that these effects are secondary to the pathogenic effect i.e. carried as a passenger on the same functional haplotype but do not contribute to autoimmunity.
