# Supplementary material for: Profiling RNA-Seq at multiple resolutions markedly increases the number of causal eQTLs in autoimmune disease
Source: PLoS Genet. 2017 Oct 23;13(10):e1007071. doi: 10.1371/journal.pgen.1007071 (PMC5695635; doi:10.1371/journal.pgen.1007071)
Supplement: S5 Table — This table shows how may expression elements (i.e. number of exons, junctions, introns etc.) that are deemed to have a causal cis-eQTL association with the SLE associated SNP (taken from Table 3). A dashed line indicates that no causal association exists at that particular quantification type. For example, rs3768792 is a causal cis-eQTL for IKZF2 at both exon- and intron-level. Out of the five exons of IKZF2 that are included in the cis-eQTL analysis (some may have been dropped form analysis due to low expression etc.), only one shows a causal cis-eQTL association with rs3768792. The same is true for one of the fifteen introns of IKZF2. For BANK1, we were able to resolve to two exons. (PDF) [file pgen.1007071.s005.pdf]

S5 Table

Number of expression elements that are deemed to have a causal association with the SLE risk SNP

Lead SNP	Gene	Gene	Transcript	Exon	Junction	Intron
rs2476601	*PHTF1*	-	-	-	-	1/10
rs1801274	*ARHGAP30*	-	-	1/12	-	-
rs9782955	*LYST*	-	-	-	1/34	-
rs3768792	*IKZF2*	-	-	1/5	-	1/15
rs10028805	*BANK1*	-	-	2/16	-	-
	BLK	1/1	4/8	13/13	13/14	8/11
rs2736340	*FAM167A*	1/1	1/1	3/3	2/2	2/4
rs2286672	*RABEP1*	-	-	1/18	-	-
	TYK2	-	-	1/23	-	1/8
rs2304256	*ATG4D*	-	-	-	1/8	-
	UBE2L3	-	1/3	2/4	-	-
rs7444	*CCDC116*	1/1	-	1/1	-	-

This table shows how may expression elements (i.e. number of exons, junctions, introns etc.) that are deemed to have a causal *cis*-eQTL association with the SLE associated SNP (taken from Table 3). A dashed line indicates that no causal association exists at that particular quantification type. For example, rs3768792 is a causal *cis*-eQTL for *IKZF2* at both exon- and intron-level. Out of the five exons of *IKZF2* that are included in the *cis*-eQTL analysis (some may have been dropped form analysis due to low expression etc.), only one shows a causal *cis*-eQTL association with rs3768792. The same is true for one of the fifteen introns of *IKZF2*. For *BANK1*, we were able to resolve to two exons.
