# Supplementary material for: Profiling RNA-Seq at multiple resolutions markedly increases the number of causal eQTLs in autoimmune disease
Source: PLoS Genet. 2017 Oct 23;13(10):e1007071. doi: 10.1371/journal.pgen.1007071 (PMC5695635; doi:10.1371/journal.pgen.1007071)
Supplement: S6 Table — Associations refers to the number of unique susceptibility loci identified per disease across studies of the same disease. Studies consist of Genome-wide Association Studies (GWAS), Immunochip studies, and meta-analyses/replication. Date refers to date of publication. (PDF) [file pgen.1007071.s006.pdf]

S6 Table

Curated studies of the ImmunoBase Resource

Disease	Associations	Study-type	Author	Ref.	Date
Alopecia Areata	13	GWAS	Petukhova, L	[1]	2010
		Immunochip	Redler, S	[2]	2014
		Meta-analysis	Betz, RC	[3]	2015
Ankylosing Spondylitis	23	GWAS	TASC	[4]	2010
		ImmunoChip	IGAS	[5]	2013
Autoimmune Thyroid Disease	8	ImmunoChip	Cooper, JD	[6]	2012
Celiac Disease	41	GWAS	Dubois, PC	[7]	2010
		ImmunoChip	Trynka, G	[8]	2011
Celiac Disease, Multiple Sclerosis, Rheumatoid Arthritis, Type 1 Diabetes	*NA*	GWAS	Fortune, MD	[9]	2015
Crohn's Disease	122	GWAS	Franke, A	[10]	2010
Crohn's Disease, Inflammatory Bowel Disease, Ulcerative Colitis	*NA*	Meta-analysis	Jostins, L	[11]	2012
Crohn's Disease, Inflammatory Bowel Disease, Ulcerative Colitis	*NA*	ImmunoChip	Liu, JZ	[12]	2015
Crohn's Disease, Multiple Sclerosis	*NA*	Replication	Uslu, VV	[13]	2014
Crohn's Disease, Rheumatoid Arthritis, Type 1 Diabetes	*NA*	GWAS	WTCCC	[14]	2007
IgE and Allergic Sensitization	10	GWAS	Bønnelykke, K	[15]	2013
Juvenile Idiopathic Arthritis	23	ImmunoChip	Hinks, A	[16]	2013
Multiple Sclerosis	106	GWAS	Sanna, S	[17]	2010
		GWAS	IMSGC	[18]	2011
		GWAS	Patsopoulos, NA	[19]	2011
		GWAS	IMSGC	[20]	2013
		ImmunoChip	IMSGC	[21]	2013
Narcolepsy	3	ImmunoChip	Faraco, J	[22]	2013
Primary Biliary Cirrhosis	31	ImmunoChip	Liu, JZ	[23]	2012
Primary Sclerosing Cholangitis	12	ImmunoChip	Liu, JZ	[24]	2013
		GWAS	Cordell, HJ	[25]	2015
Psoriasis	35	GWAS	WTCCC	[26]	2010
		ImmunoChip	Tsoi, LC	[27]	2012
Rheumatoid Arthritis	81	Meta-analysis	Stahl, EA	[28]	2010
		ImmunoChip	Eyre, S	[29]	2012
		Meta-analysis	Okada, Y	[30]	2013
Sjogren Syndrome	8	GWAS	Lessard, CJ	[31]	2013
Systemic Lupus Erythematosus	47	GWAS	Gateva, V	[32]	2009
		GWAS	Armstrong, DL	[33]	2014
		GWAS	Bentham, J	[34]	2015
Systemic Scleroderma	6	ImmunoChip	Mayes, MD	[35]	2014
Type 1 Diabetes	57	GWAS	Todd, JA	[36]	2007
		Meta-analysis	Cooper, JD	[37]	2008
		GWAS	Barrett, JC	[38]	2009
		GWAS	Bradfield, JP	[39]	2011
		Replication	Evangelou, M	[40]	2014
		ImmunoChip	Onengut-Gumuscu, S	[41]	2015
Ulcerative Colitis	102	GWAS	Anderson, CA	[42]	2011
Vitiligo	25	Meta-analysis	Jin, Y	[43]	2012

Associations refers to the number of unique susceptibility loci identified per disease across studies of the same disease. Studies consist of Genome-wide Association Studies (GWAS), Immunochip studies, and meta-analyses/replication. Date refers to date of publication.

References to Immunobase association studies:

1. Petukhova L, Duvic M, Hordinsky M, Norris D, Price V, Shimomura Y, et al. Genome-wide association study in alopecia areata implicates both innate and adaptive immunity. Nature. Nature Publishing Group; 2010;466: 113–7. doi:10.1038/nature09114
2. Redler S, Angisch M, Heilmann S, Wolf S, Barth S, Basmanav BF, et al. Immunochip-based analysis: High-density genotyping of immune-related loci sheds further light on the autoimmune genetic architecture of alopecia areata. J Invest Dermatol. 2015;135: 919–921. doi:10.1038/jid.2014.459
3. Betz RC, Petukhova L, Ripke S, Huang H, Menelaou A, Redler S, et al. Genome-wide meta-analysis in alopecia areata resolves HLA associations and reveals two new susceptibility loci. Nat Commun. 2015;6: 5966. doi:10.1038/ncomms6966
4. Reveille JD, Sims A-M, Danoy P, Evans DM, Leo P, Pointon JJ, et al. Genome-wide association study of ankylosing spondylitis identifies non-MHC susceptibility loci. Nat Genet. 2010;42: 123–7. doi:10.1038/ng.513
5. Cortes A, Hadler J, Pointon JP, Robinson PC, Karaderi T, Leo P, et al. Identification of multiple risk variants for ankylosing spondylitis through high-density genotyping of immune-related loci. Nat Genet. 2013;45: 730–8. doi:10.1038/ng.2667
6. Cooper JD, Simmonds MJ, Walker NM, Burren O, Brand OJ, Guo H, et al. Seven newly identified loci for autoimmune thyroid disease. Hum Mol Genet. 2012;21: 5202–5208. doi:10.1093/hmg/dds357
7. Dubois PCA, Trynka G, Franke L, Hunt KA, Romanos J, Curtotti A, et al. Multiple common variants for celiac disease influencing immune gene expression. Nat Genet. Nature Publishing Group; 2010;42: 295–302. doi:10.1038/ng.543
8. Trynka G, Hunt KA, Bockett NA, Romanos J, Mistry V, Szperl A, et al. Dense genotyping identifies and localizes multiple common and rare variant association signals in celiac disease. Nat Genet. Nature Publishing Group; 2011;43: 1193–1201. doi:10.1038/ng.998
9. Fortune MD, Guo H, Burren O, Schofield E, Walker NM, Ban M, et al. Statistical colocalization of genetic risk variants for related autoimmune diseases in the context of common controls. Nat Genet. 2015;47: 839–846. doi:10.1038/ng.3330
10. Franke A, McGovern DPB, Barrett JC, Wang K, Radford-Smith GL, Ahmad T, et al. Genome-wide meta-analysis increases to 71 the number of confirmed Crohn's disease susceptibility loci. Nat Genet. Nature Publishing Group; 2010;42: 1118–25. doi:10.1038/ng.717
11. Jostins L, Ripke S, Weersma RK, Duerr RH, McGovern DP, Hui KY, et al. Host-microbe interactions have shaped the genetic architecture of inflammatory bowel disease. Nature. Nature Publishing Group; 2012;491: 119–24. doi:10.1038/nature11582
12. Liu JZ, van Sommeren S, Huang H, Ng SC, Alberts R, Takahashi A, et al. Association analyses identify 38 susceptibility loci for inflammatory bowel disease and highlight shared genetic risk across populations. Nat Genet. 2015;47: 979–989. doi:10.1038/ng.3359
13. Uslu VV, Petretich M, Ruf S, Langenfeld K, Fonseca NA, Marioni JC, et al. Long-range enhancers regulating Myc expression are required for normal facial morphogenesis. Nat Genet. Nature Publishing Group; 2014;46: 1–24. doi:10.1038/ng.2971
14. Wellcome T, Case T, Consortium C. Genome-wide association study of 14,000 cases of seven common diseases and 3,000 shared controls. Nature. 2007;447: 661–78. doi:10.1038/nature05911
15. Bønnelykke K, Matheson MC, Pers TH, Granell R, Strachan DP, Alves AC, et al. Meta-analysis of genome-wide association studies identifies ten loci influencing allergic sensitization. Nat Genet. Nature Publishing Group; 2013;45: 902–6. doi:10.1038/ng.2694
16. Hinks A, Cobb J, Marion MC, Prahalad S, Sudman M, Bowes J, et al. Dense genotyping of immune-related disease regions identifies 14 new susceptibility loci for juvenile idiopathic arthritis. Nat Genet. Nature Publishing Group; 2013;45: 664–9. doi:10.1038/ng.2614
17. Sanna S, Pitzalis M, Zoledziewska M, Zara I, Sidore C, Murru R, et al. Variants within the immunoregulatory CBLB gene are associated with multiple sclerosis. Nat Genet. 2010;42: 495–7. doi:10.1038/ng.584
18. Sawcer S, Hellenthal G, Pirinen M, Spencer CCA, Patsopoulos N a, Moutsianas L, et al. Genetic risk and a primary role for cell-mediated immune mechanisms in multiple sclerosis. Nature. 2011;476: 214–9. doi:10.1038/nature10251
19. Patsopoulos NA, Esposito F, Reischl J, Lehr S, Bauer D, Heubach J, et al. Genome-wide meta-analysis identifies novel multiple sclerosis susceptibility loci. Ann Neurol. 2011;70: 897–912. doi:10.1002/ana.22609
20. Lill CM, Schjeide BMM, Graetz C, Ban M, Alcina A, Ortiz MA, et al. MANBA, CXCR5, SOX8, RPS6KB1 and ZBTB46 are genetic risk loci for multiple sclerosis. Brain. 2013;136: 1778–1782. doi:10.1093/brain/awt101

21. Beecham AH, Patsopoulos NA, Xifara DK, Davis MF, Kemppinen A, Cotsapas C, et al. Analysis of immune-related loci identifies 48 new susceptibility variants for multiple sclerosis. Nat Genet. 2013;45: 1353–60. doi:10.1038/ng.2770
22. Faraco J, Lin L, Kornum BR, Kenny EE, Trynka G, Einen M, et al. ImmunoChip Study Implicates Antigen Presentation to T Cells in Narcolepsy. PLoS Genet. 2013;9. doi:10.1371/journal.pgen.1003270
23. Liu JZ, Almarri MA, Gaffney DJ, Mells GF, Jostins L, Cordell HJ, et al. Dense fine-mapping study identifies new susceptibility loci for primary biliary cirrhosis. Nat Genet. 2012;44: 1137–41. doi:10.1038/ng.2395
24. Liu JZ, Hov JR, Folseraas T, Ellinghaus E, Rushbrook SM, Doncheva NT, et al. Dense genotyping of immune-related disease regions identifies nine new risk loci for primary sclerosing cholangitis. Nat Genet. 2013;45: 670–5. doi:10.1038/ng.2616
25. Cordell HJ, Han Y, Mells GF, Li Y, Hirschfield GM, Greene CS, et al. International genome-wide meta-analysis identifies new primary biliary cirrhosis risk loci and targetable pathogenic pathways. Nat Commun. Nature Publishing Group; 2015;6: 8019. doi:10.1038/ncomms9019
26. Genetic Analysis of Psoriasis Consortium & the Wellcome Trust Case Control Consortium 2, Strange A, Capon F, Spencer CC, Knight J, Weale ME, et al. A genome-wide association study identifies new psoriasis susceptibility loci and an interaction between HLA-C and ERAP1. Nat Genet. 2010;42: 985–90. doi:10.1038/ng.694
27. Tsoi LC, Spain SL, Knight J, Ellinghaus E, Stuart PE, Capon F, et al. Identification of 15 new psoriasis susceptibility loci highlights the role of innate immunity. Nat Genet. 2012;44: 1341–8. doi:10.1038/ng.2467
28. Stahl EA, Raychaudhuri S, Remmers EF, Xie G, Eyre S, Thomson BP, et al. Genome-wide association study meta-analysis identifies seven new rheumatoid arthritis risk loci. Nat Genet. 2010;42: 508–514. doi:10.1038/ng.582
29. Eyre S, Bowes J, Diogo D, Lee A, Barton A, Martin P, et al. High-density genetic mapping identifies new susceptibility loci for rheumatoid arthritis. Nat Genet. Nature Publishing Group; 2012;44: 1336–40. doi:10.1038/ng.2462
30. Okada Y, Wu D, Trynka G, Raj T, Terao C, Ikari K, et al. Genetics of rheumatoid arthritis contributes to biology and drug discovery. Nature. Nature Publishing Group; 2014;506: 376–381. doi:10.1038/nature12873
31. Lessard CJ, Li H, Adrianto I, Ice JA, Rasmussen A, Grundahl KM, et al. Variants at multiple loci implicated in both innate and adaptive immune responses are associated with Sjogren's syndrome. Nat Genet. 2013;45: 1284–1292. doi:10.1038/ng.2792
32. Gateva V, Sandling JK, Hom G, Taylor KE, Chung SA, Sun X, et al. A large-scale replication study identifies TNIP1, PRDM1, JAZF1, UHRF1BP1 and IL10 as risk loci for systemic lupus erythematosus. Nat Genet. Nature Publishing Group; 2009;41: 1228–33. doi:10.1038/ng.468
33. Armstrong DL, Zidovetzki R, Alarcón-Riquelme ME, Tsao BP, Criswell LA, Kimberly RP, et al. GWAS identifies novel SLE susceptibility genes and explains the association of the HLA region. Genes Immun. 2014;15: 347–54. doi:10.1038/gene.2014.23
34. Bentham J, Morris DL, Cunninghame Graham DS, Pinder CL, Tombleson P, Behrens TW, et al. Genetic association analyses implicate aberrant regulation of innate and adaptive immunity genes in the pathogenesis of systemic lupus erythematosus. Nat Genet. Nature Publishing Group; 2015;47: 1457–1464. doi:10.1038/ng.3434
35. Mayes MD, Bossini-Castillo L, Gorlova O, Martin JE, Zhou X, Chen W V., et al. Immunochip analysis identifies multiple susceptibility loci for systemic sclerosis. Am J Hum Genet. 2014;94: 47–61. doi:10.1016/j.ajhg.2013.12.002
36. Todd JA, Walker NM, Cooper JD, Smyth DJ, Downes K, Plagnol V, et al. Robust associations of four new chromosome regions from genome-wide analyses of type 1 diabetes. Nat Genet. 2007;39: 857–864. doi:10.1038/ng2068
37. Cooper JD, Smyth DJ, Smiles AM, Plagnol V, Walker NM, Allen JE, et al. Meta-analysis of genome-wide association study data identifies additional type 1 diabetes risk loci. Nat Genet. 2008;40: 1399–401. doi:10.1038/ng.249
38. Barrett JC, Clayton DG, Concannon P, Akolkar B, Cooper JD, Erlich HA, et al. Genome-wide association study and meta-analysis find that over 40 loci affect risk of type 1 diabetes. NatGenet. 2009;41: 703–707. doi:10.1038/ng.381
39. Bradfield JP, Qu HQ, Wang K, Zhang H, Sleiman PM, Kim CE, et al. A genome-wide meta-analysis of six type 1 diabetes cohorts identifies multiple associated loci. PLoS Genet. 2011;7. doi:10.1371/journal.pgen.1002293
40. Evangelou M, Smyth DJ, Fortune MD, Burren OS, Walker NM, Guo H, et al. A Method for gene-based pathway analysis using genomewide association study summary statistics reveals nine new type 1

diabetes associations. Genet Epidemiol. 2014;38: 661–670. doi:10.1002/gepi.21853
41. Onengut-Gumuscu S, Chen W, Burren O, Cooper NJ, Quinlan AR, Mychaleckyj JC, et al. Fine mapping of type 1 diabetes susceptibility loci and evidence for colocalization of causal variants with lymphoid gene enhancers. Nat Genet. Nature Publishing Group; 2015;47: 381–6. doi:10.1038/ng.3245
42. Anderson CA, Boucher G, Lees CW, Franke A, D'Amato M, Taylor KD, et al. Meta-analysis identifies 29 additional ulcerative colitis risk loci, increasing the number of confirmed associations to 47. Nat Genet. 2011;43: 246–52. doi:10.1038/ng.764
43. Jin Y, Birlea SA, Fain PR, Ferrara TM, Ben S, Riccardi SL, et al. Genome-wide association analyses identify 13 new susceptibility loci for generalized vitiligo. Nat Genet. Nature Publishing Group; 2012;44: 676–80. doi:10.1038/ng.2272
