# Supplementary material for: Profiling RNA-Seq at multiple resolutions markedly increases the number of causal eQTLs in autoimmune disease
Source: PLoS Genet. 2017 Oct 23;13(10):e1007071. doi: 10.1371/journal.pgen.1007071 (PMC5695635; doi:10.1371/journal.pgen.1007071)
Supplement: S8 Table — The results of this table are depicted in S11 Fig. A genome-wide cis-eQTL analysis was performed as described in methods for all common SNPs (MAF > 5%) against gene quantifications profiled at gene-, transcript-, exon-, junction-, and intron-level. ‘TOTAL’ refers to the total number of elements tested in a genome-wide setting. The number of SNPs is different per quantification type as the analysis is run in cis, meaning only expression elements within +/-500kb of each SNP are considered; therefore, if there is no expression element within this distance, the SNP is not included in the analysis. ‘Genes’ refers to the number of distinct (unique) genes tested against, and ‘targets’ refers to the number of individual genes, transcripts, exons, junctions, and introns tested against measured using the corresponding profiling type. ‘SIGNIFICANT’ refers to the number of SNPs, genes etc. that pass a genome-wide false discovery rate (FDR) multiple testing threshold of 5%. ‘PERCENTAGE SIGNIFICANT’ refers to the percentage of SNPs, genes etc. that are significant as a percentage of the total tested. (PDF) [file pgen.1007071.s008.pdf]

S8 Table

Summary results of genome-wide *cis*-eQTL analysis

	Test	Gene	Transcript	Exon	Junction	Intron
**TOTAL**	**SNPs**	1745261	2813231	3337666	3264551	3611418
	**Genes**	14546	13783	14042	10564	13469
	**Targets**	14546	63438	141117	127404	93747
	**Distinct SNP-Gene pairs**	3016012	6759383	10220224	9090904	9603702
	**Total SNP-Target pairs**	3016012	9072417	31227090	24439851	17057283
**SIGNIFICANT (FDR < 0.05)**	**SNPs**	458654	429686	1256346	935370	1144452
	**Genes**	8351	8874	12918	9659	11111
	**Targets**	8351	17218	89099	63839	42950
	**Distinct SNP-Gene pairs**	610696	586659	2158552	1417712	1756270
	**Total SNP-Target pairs**	610696	763559	7524183	935370	2972825
**PERCENTAGE SIGNIFICANT**	**SNPs**	26.28	15.27	37.64	28.65	31.69
	**Genes**	57.41	64.38	92.00	91.43	82.49
	**Targets**	57.41	27.14	63.14	50.11	45.81
	**Distinct SNP-Gene pairs**	20.25	8.68	21.12	15.59	18.29
	**Total SNP-Target pairs**	20.25	8.42	24.10	3.83	17.43

The results of this table are depicted in S11 Fig. A genome-wide *cis*-eQTL analysis was performed as described in methods for all common SNPs (MAF > 5%) against gene quantifications profiled at gene-, transcript-, exon-, junction-, and intron-level. 'TOTAL' referes to the total number of elements tested in a genome-wide setting. The number of SNPs is different per quantification type as the analysis is run in *cis*, meaning only expression elements within +/-500kb of each SNP are considered; therefore, if there is no expression element within this distance, the SNP is not included in the analysis. 'Genes' referes to the number of distinct (unique) genes tested against, and 'targets' refers to the number of individual genes, transcripts, exons, junctions, and introns tested against measured using the corresponding profiling type. 'SIGNIFICANT' refers to the number of SNPs, genes etc. that pass a genome-wide false discovery rate (FDR) multiple testing threshold of 5%. 'PERCENTAGE SIGNIFICANT' refers to the percentage of SNPs, genes etc. that are significant as a percentage of the total tested.
