# Supplementary figures and images for: Profiling RNA-Seq at multiple resolutions markedly increases the number of causal eQTLs in autoimmune disease
Source: PLoS Genet. 2017 Oct 23;13(10):e1007071. doi: 10.1371/journal.pgen.1007071 (PMC5695635; doi:10.1371/journal.pgen.1007071)

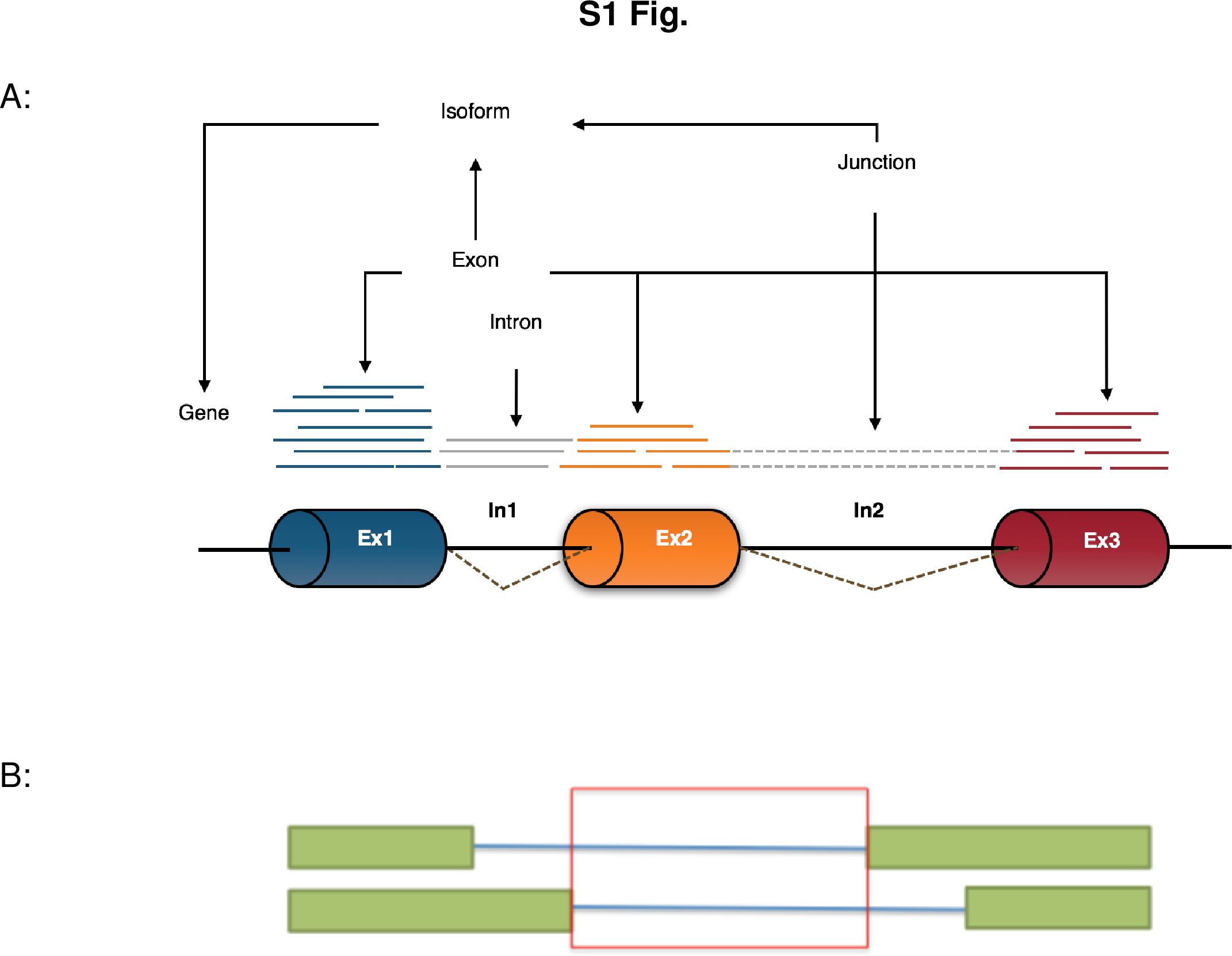

Supplement: S1 Fig — The following text (*) has been lifted from the supplementary material from Lappalainen et al 2013 regarding how expression using RNA-Seq was estimated across the five RNA-Seq quantification types (gene-, transcript-, exon-, junction-, and intron-level; S1A Fig). Further information is available on the Geuvadis wiki page: http://geuvadiswiki.crg.es/index.php/Main_Page and on the Flux Capacitor webpage for the Geuvadis Project: http://sammeth.net/confluence/display/FLUX/Geuvadis+Quantifications. In this work, normalised RNA-Seq expression data of 373 lymphoblastoid cell lines from four European sub-populations (CEU, GBR, FIN, TSI) of the 1000Genomes Project (Geuvadis) were obtained from EBI ArrayExpress (E-GEUV-1). Quantification was performed at gene-, transcript-, exon-, junction-, and intron-level as described below. Quantifications were corrected for sequencing depth and gene length (RPKM). Only expression elements quantified in > 50% of individuals were kept and Probabilistic Estimation of Expression Residuals (PEER) was used to remove technical variation and expression residuals transformed to a standard normal distribution. (*) Quantifications of transcripts and splice junctions by the Flux Capacitor approach are based on the annotation‐mapped genomic mappings considering transcript structures of the GENCODE transcriptome annotation: mappings of read pairs that were completely included within the annotated exon boundaries and paired in the expected orientation have been considered. Reads belonging to single transcripts were predicted by deconvolution according to observations of paired reads mapping across all exonic segments of a locus. Gene quantifications were calculated as the sum of all transcript RPKMs per gene. Annotated splice junctions were quantified using split read information, counting the number of reads supporting a given junction. Exon quantifications were calculated for protein‐coding and lincRNA transcripts. All overlapping exons of a gene we [file pgen.1007071.s010.tif]

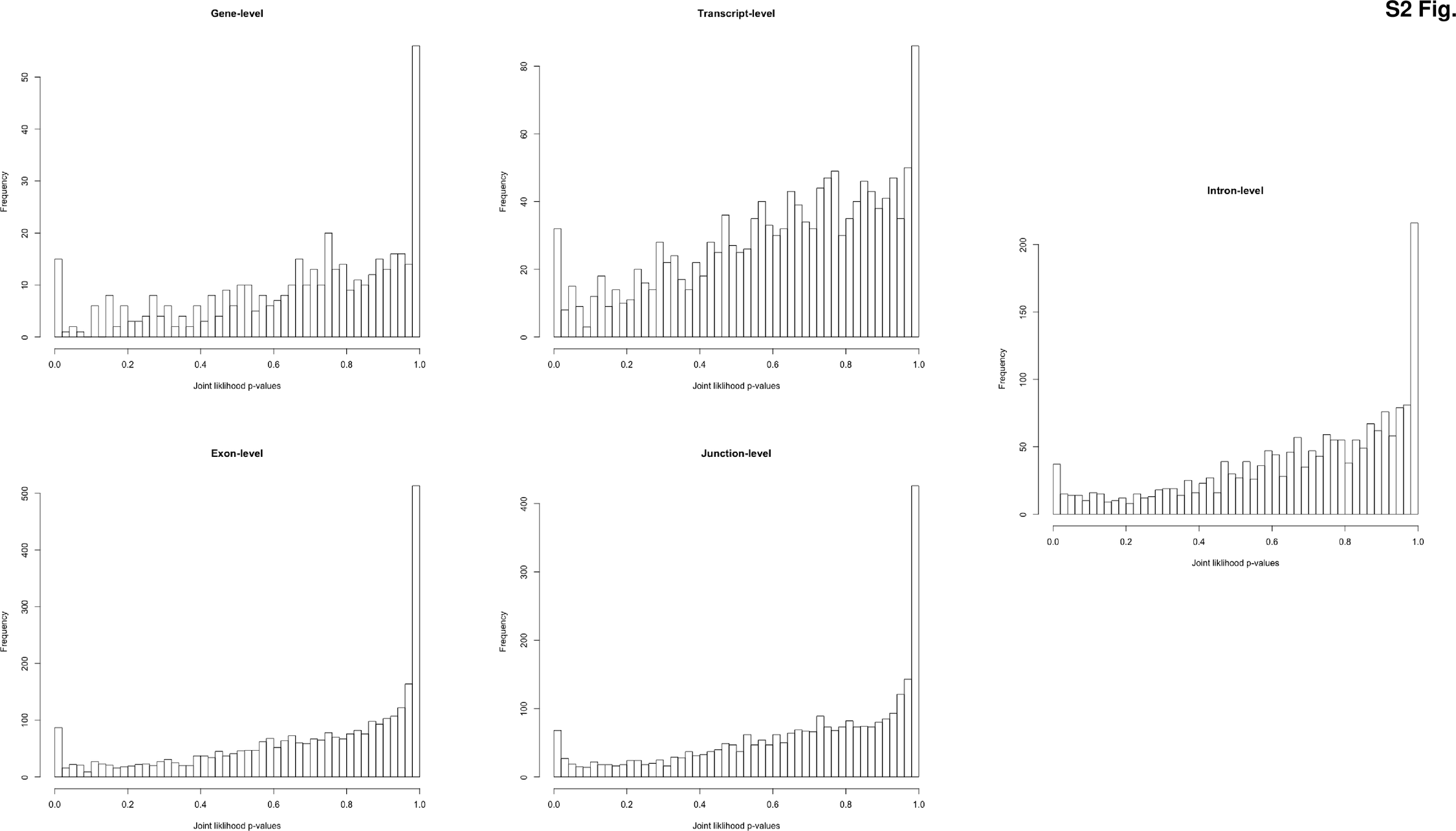

Supplement: S2 Fig — (TIF) [file pgen.1007071.s011.tif]

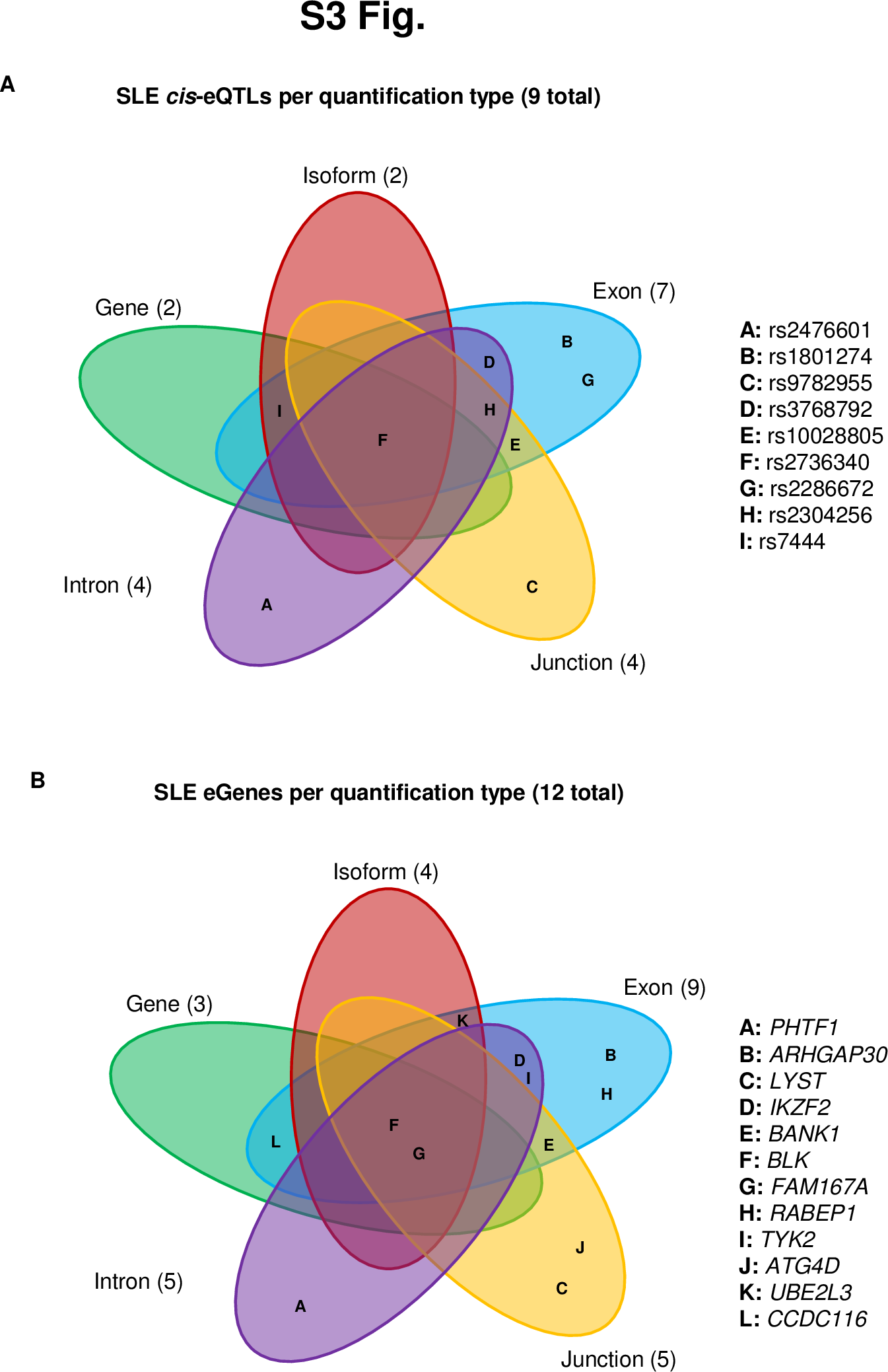

Supplement: S3 Fig — (TIF) [file pgen.1007071.s012.tif]

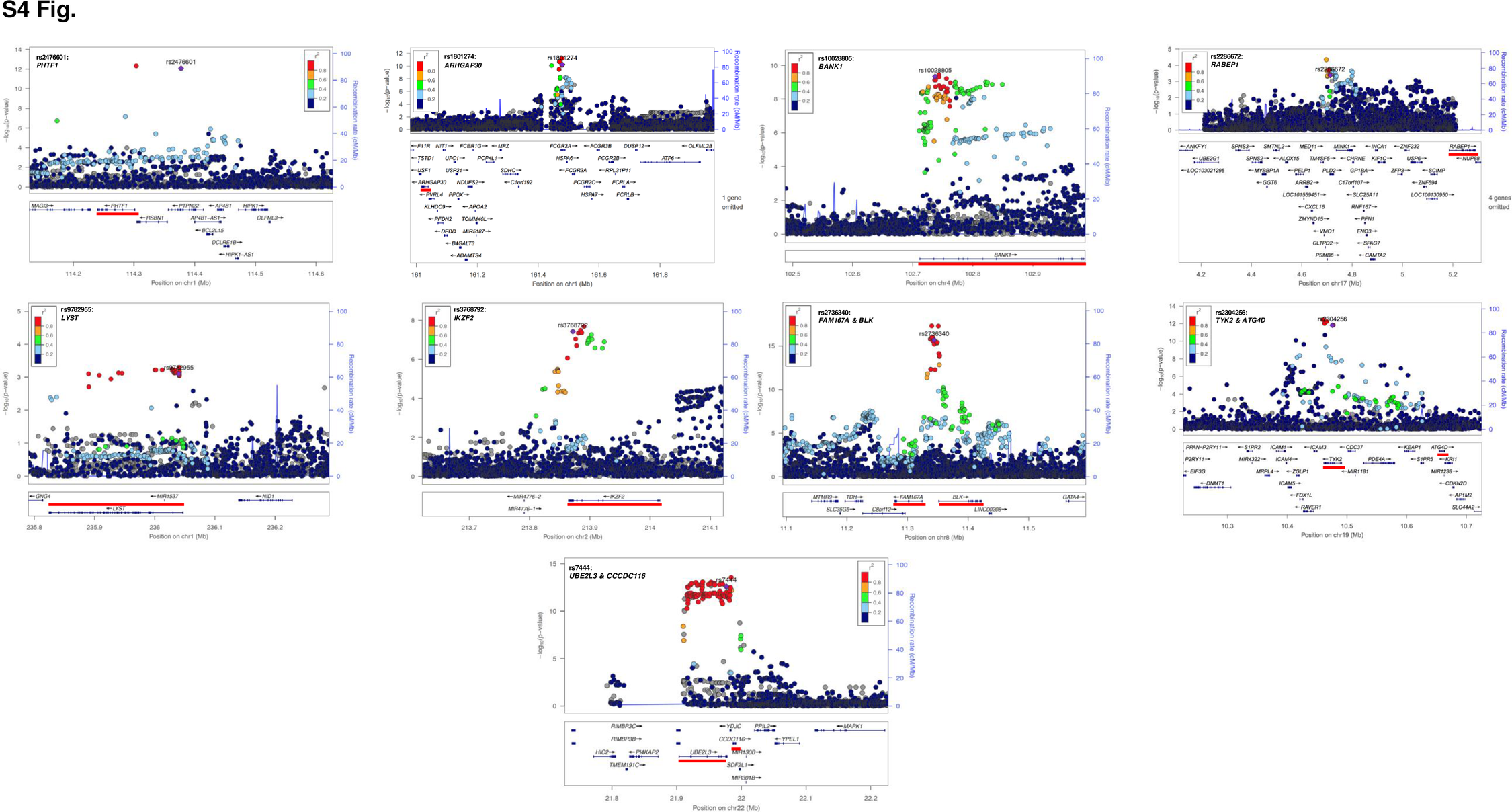

Supplement: S4 Fig — Showing the nine loci that are causal cis-eQTLs and candidate genes from JLIM analysis. The full results of this analysis are in Table 3 of the manuscript and the summary results from the GWAS as provided in S1 Table. Candidate genes are highlighted in red. (TIF) [file pgen.1007071.s013.tif]

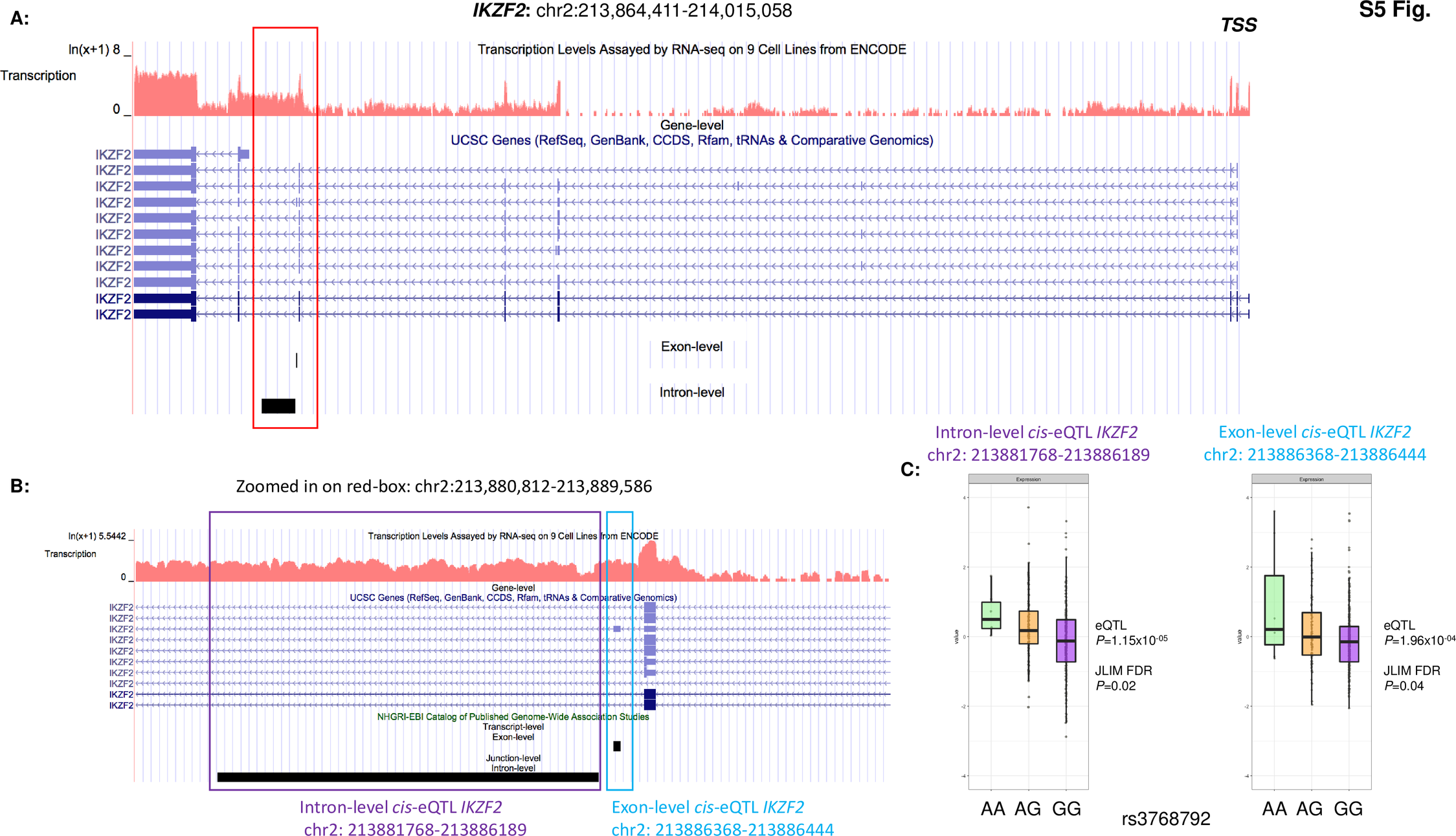

Supplement: S5 Fig — Full results of the causal cis-eQTL associations are found in S2 Table. This figure shows how cis-eQTL analysis can be used to resolve to a single expression element targeted by a disease associated SNP. (A) The genomic coordinates and isoform structure of SLE candidate gene IKZF2 detected by cis-eQTL analysis using RNA-Seq at exon-level and intron-level. The transcription start site of IKZF2 is on the right-hand side. In the red box is the single exon and single intron modulated by causal cis-eQTL rs3768792 –these are shown in the black boxes. (B) A zoomed in view of the red box showing the exon, coordinates: chr2: 213886368–213886444 and intron, coordinates: chr2: 213881768–213886189. The track above shows the transcription levels assayed by RNA-Seq in LCLs (GM12878 cell line) from the ENCODE project–the affected intron is clearly transcribed. (C) The SLE risk allele rs3768792 [A] leads to increased expression of both the depicted exon and the intron of IKZF2. The cis-eQTL association P-value and JLIM P-value are shown. (TIF) [file pgen.1007071.s014.tif]

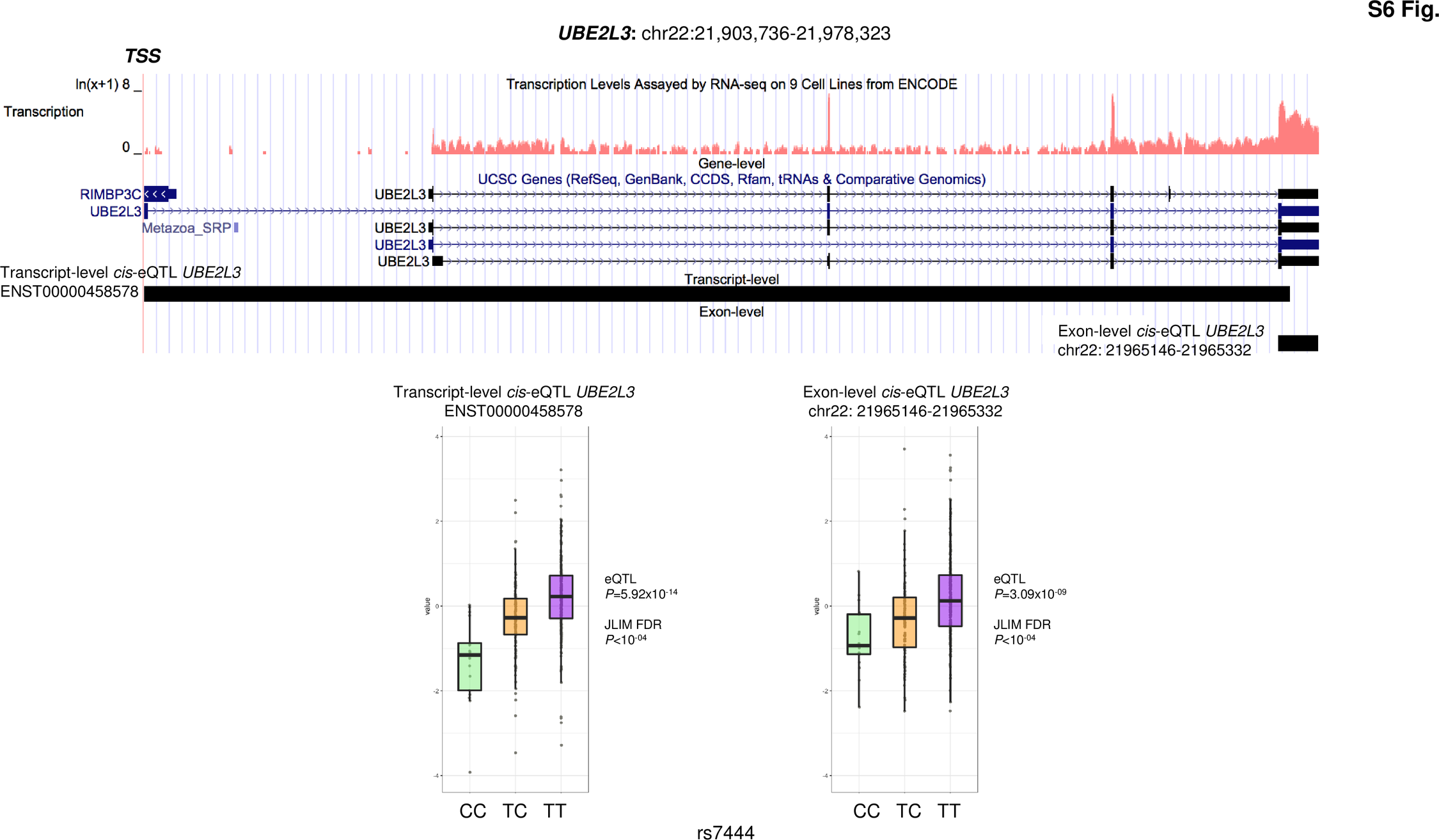

Supplement: S6 Fig — Full results of the causal cis-eQTL associations are found in S2 Table. This figure shows how cis-eQTL analysis can be used to resolve to a single expression element targeted by a disease associated SNP. (A) The genomic coordinates and isoform structure of SLE candidate gene UBE2L3 detected by cis-eQTL analysis using RNA-Seq at exon-level and transcript-level. The transcription start site of UBE2L3 is on the left-hand side. The track above shows the transcription levels assayed by RNA-Seq in LCLs (GM12878 cell line) from the ENCODE project. The SLE risk allele rs7444 [T] leads to increased expression of both the depicted transcript (ENST00000458578) and the exon of UBE2L3. The cis-eQTL association P-value and JLIM P-value are shown. (TIF) [file pgen.1007071.s015.tif]

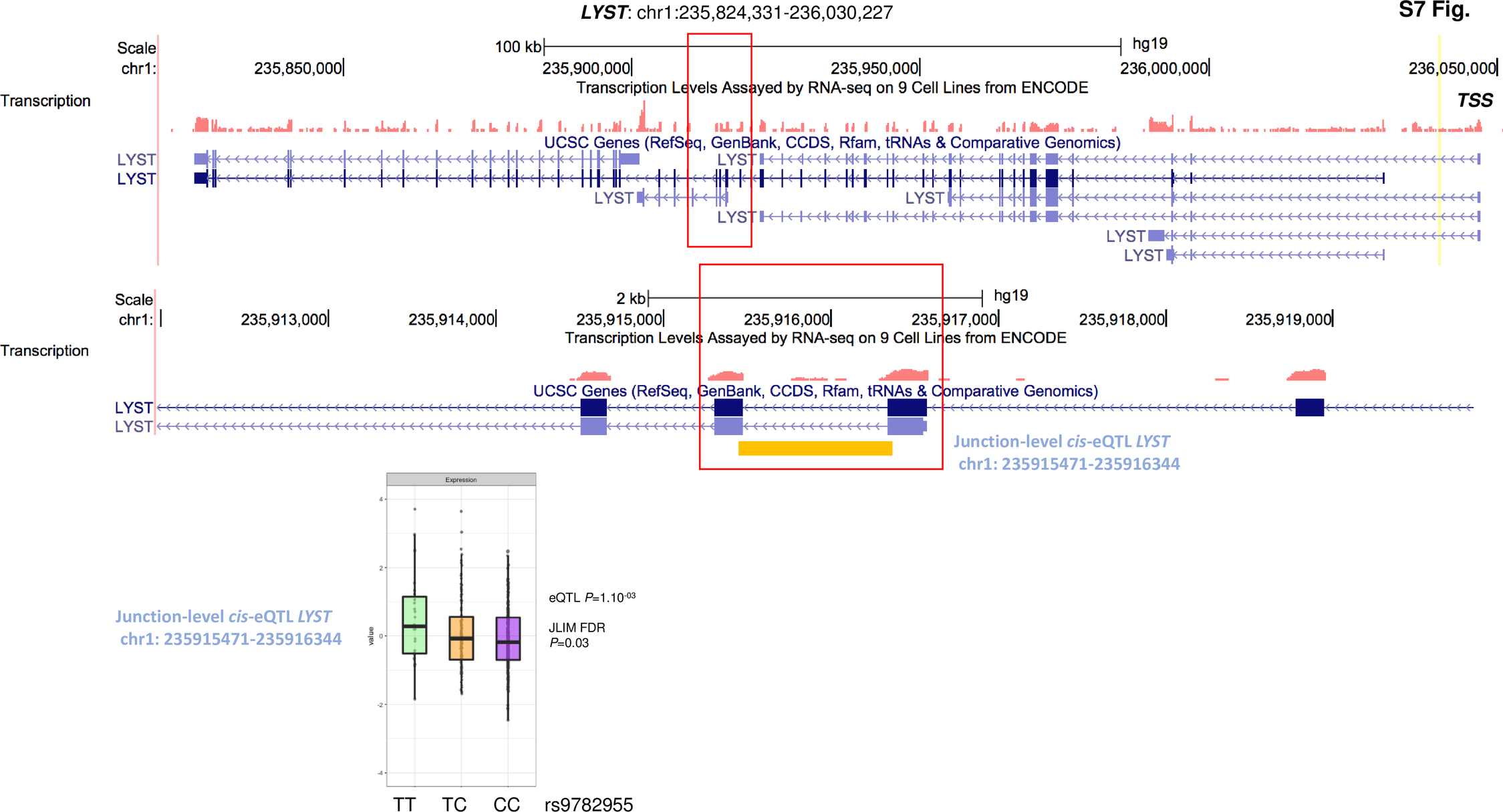

Supplement: S7 Fig — Full results of the causal cis-eQTL associations are found in S2 Table. This figure shows how cis-eQTL analysis can be used to resolve to a single expression element targeted by a disease associated SNP. Top panel—the genomic coordinates and isoform structure of SLE candidate gene LYST detected by cis-eQTL analysis using RNA-Seq at junction-level. The transcription start site of LYST is on the right-hand side. The track above shows the transcription levels assayed by RNA-Seq in LCLs (GM12878 cell line) from the ENCODE project. The SLE risk allele rs9872955 [C] leads to decreased expression of the depicted junction (chr1: 235915471–235916344). The cis-eQTL association P-value and JLIM P-value are shown. (TIF) [file pgen.1007071.s016.tif]

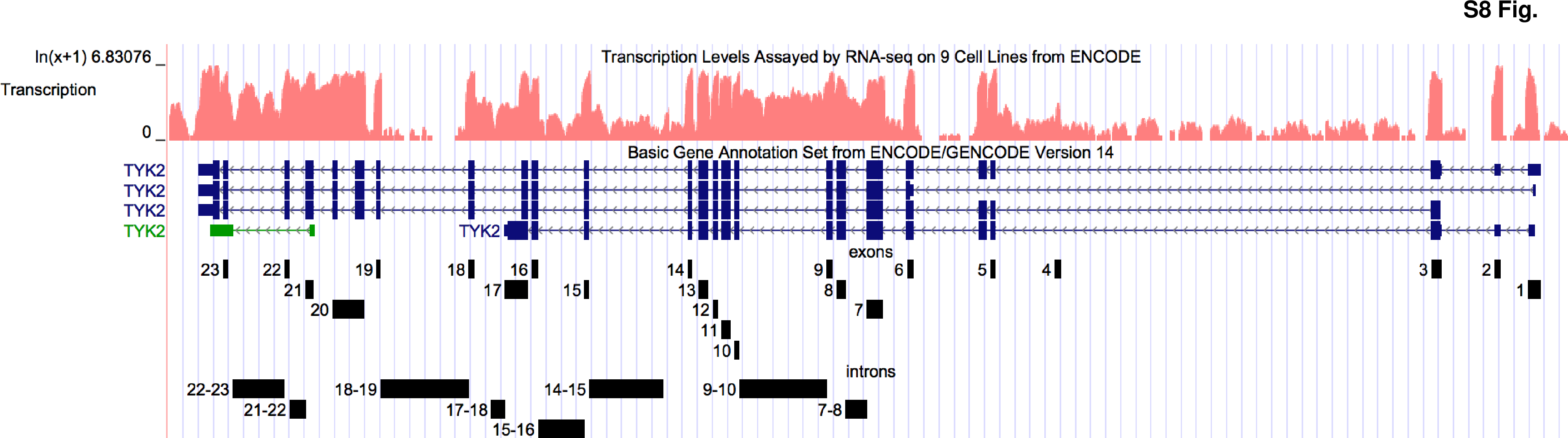

Supplement: S8 Fig — The transcription start site is on the right of the diagram. This corresponds to Fig 2. (TIF) [file pgen.1007071.s017.tif]

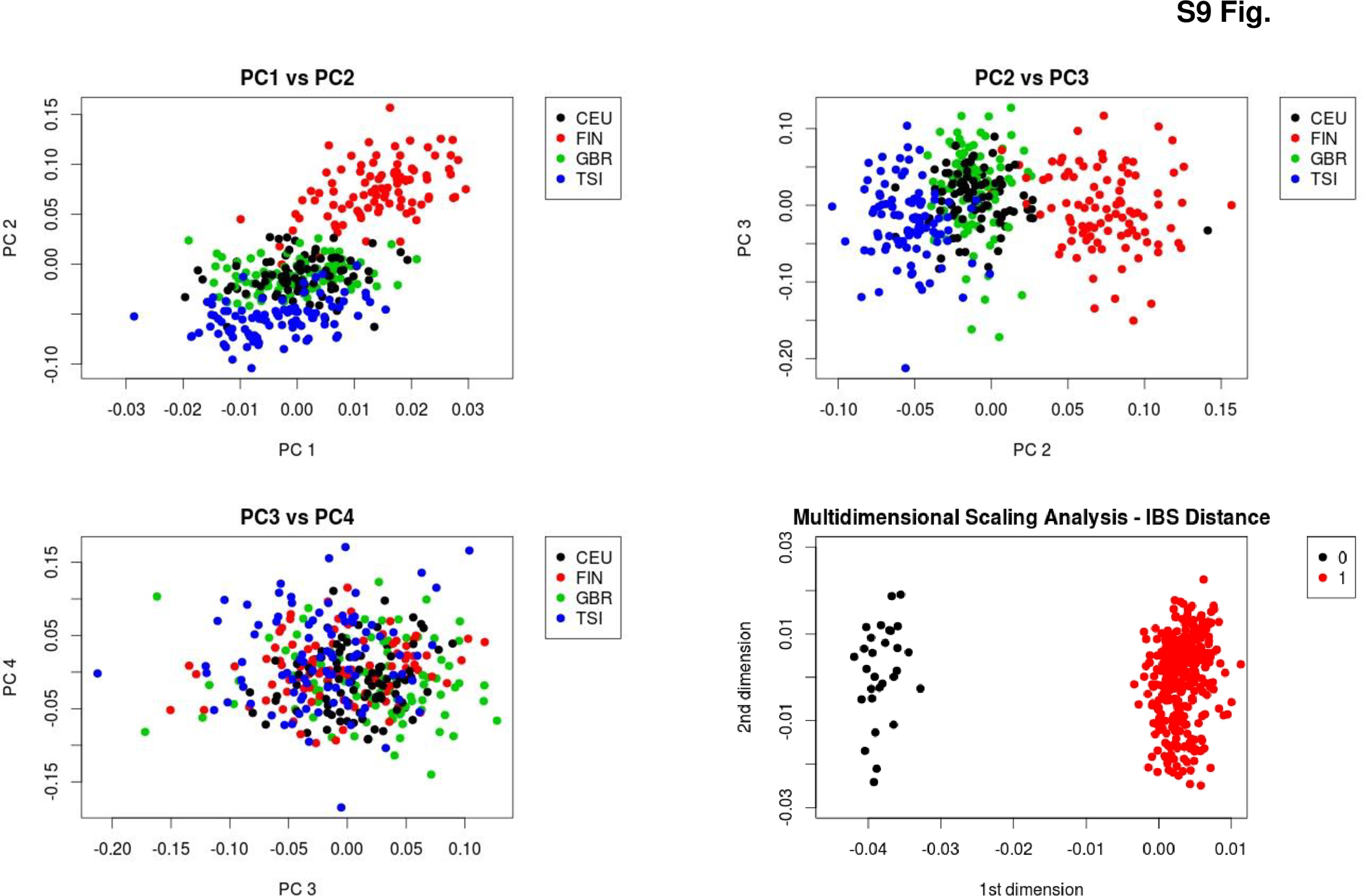

Supplement: S9 Fig — Genotype data in VCF format of 1000Genomes individuals were downloaded from E-GEUV1 (ArrayExpress). Insertion-deletion sites were removed, and bi-allelic SNPs kept only. SNPs with HWE < 0.0001 were removed and the VCF converted to 0,1,2 format using PLINK. Principle component analysis was performed on genotype data using the R package SNPRelate on chromosome 20. The first 3 components were included in the eQTL regression model as well as the binary imputation status (see methods). (TIF) [file pgen.1007071.s018.tif]

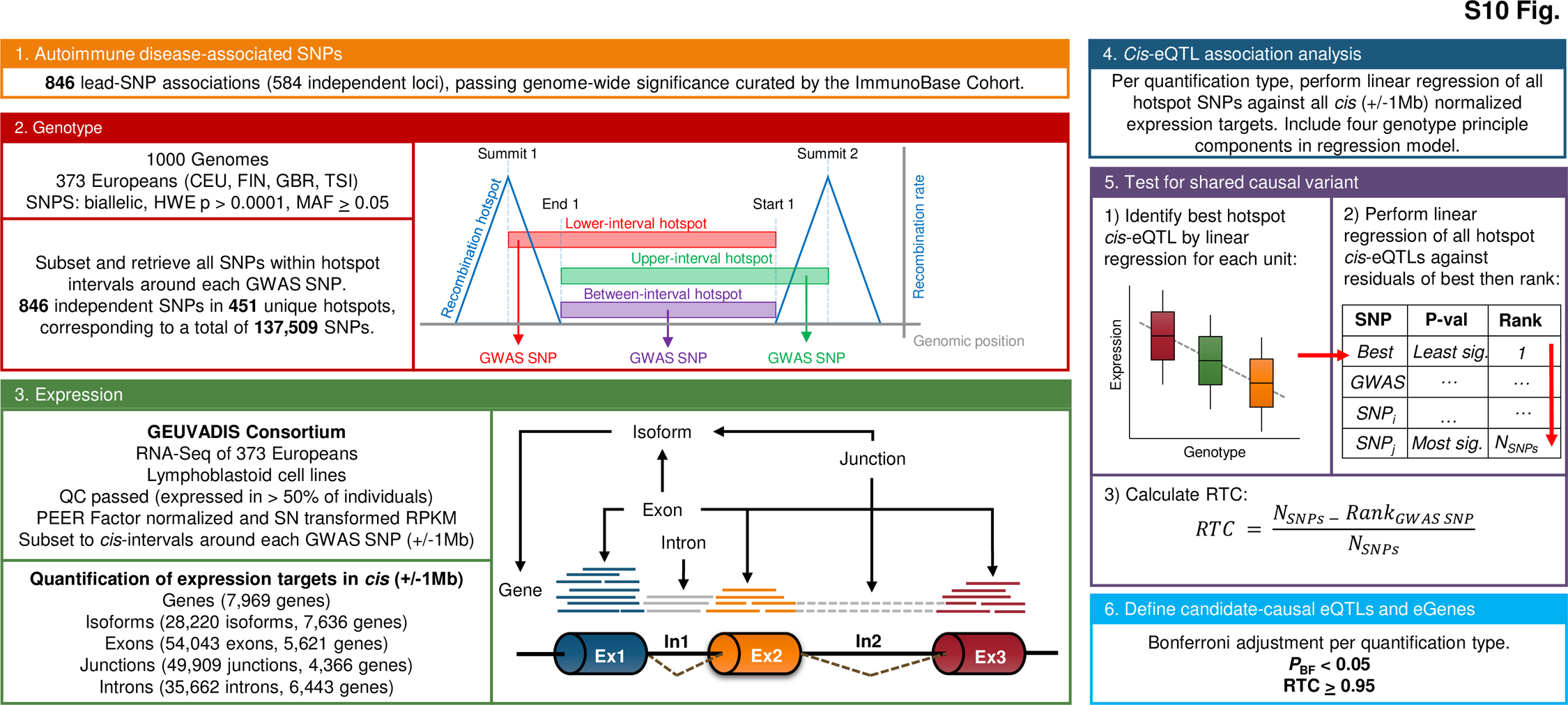

Supplement: S10 Fig — The 752-autoimmune disease associated SNPs per disease are documented in S1 Table and were LD pruned to 560 independent loci (see methods). Genotypes of 1000Genomes individuals were quality controlled and subset to regions of recombination hotspots. If the lead GWAS SNP was found between a recombination hotspot, then all SNPs were between the recombination hotspot intervals were used in the Regulatory Trait Concordance (RTC) analysis. If the lead GWAS SNP was found within a recombination hotspot itself, then all SNPs before or after the summit (including the between summit SNPs) were used in the RTC (upper-interval and lower-interval hotspot respectively). Normalized RNA-Seq expression data at gene-, isoform-, exon-, junction-, and intron-level were obtained for the 1000Genomes individuals of the Geuvadis cohort in lymphoblastoid cell lines. Disease associated SNPs with statistically significant association with gene expression (PBF < 0.05) and an RTC score > 0.95 were defined as causal cis-eQTLs. (TIF) [file pgen.1007071.s019.tif]

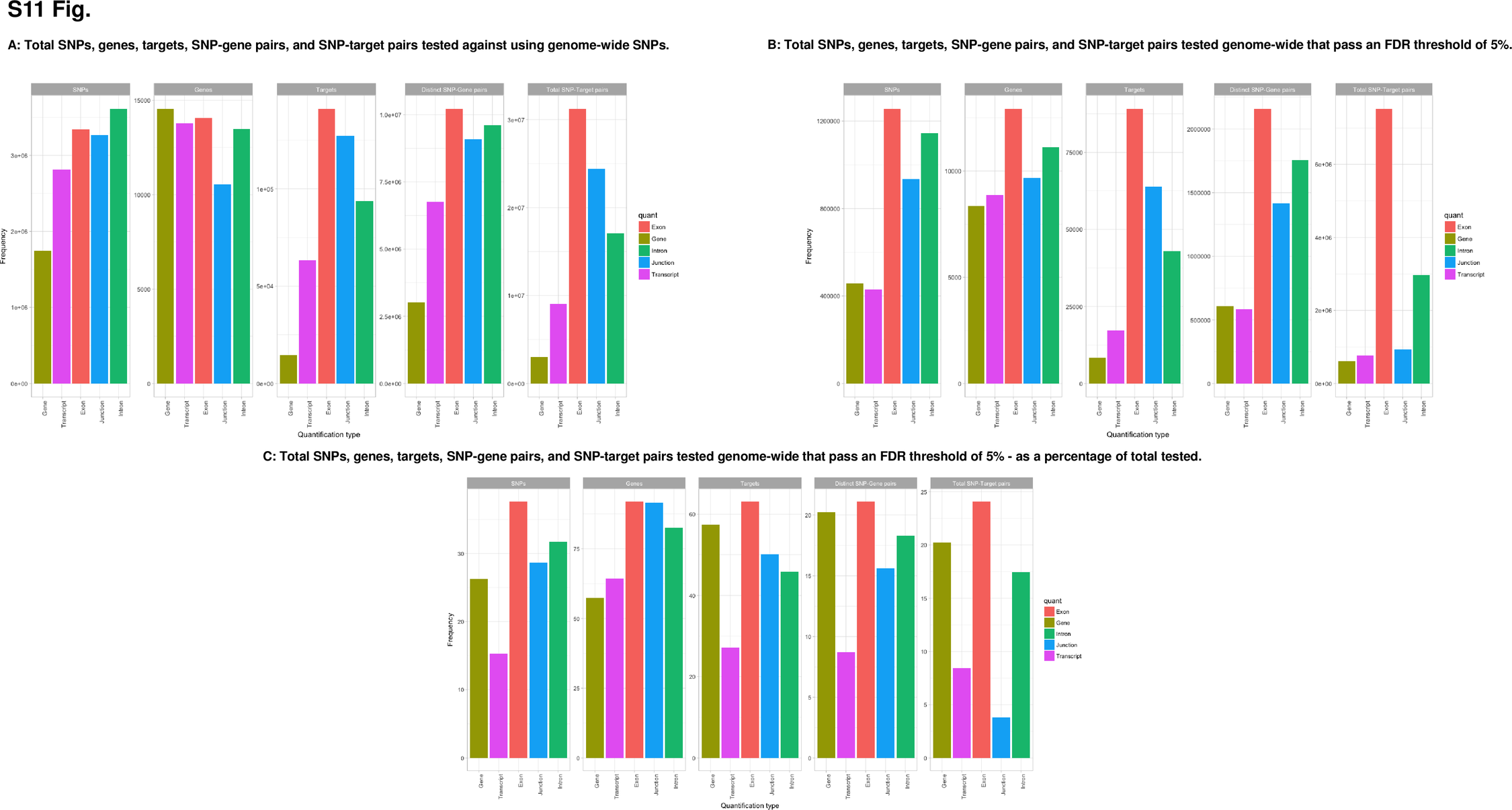

Supplement: S11 Fig — This figure corresponds to the data presented in S8 Table. A target is a single gene, transcript, exon, junction, or intron, quantified using the corresponding profiling type. SNPs, genes, targets, and SNP-gene pairs are only counted once (distinct) if multiple SNP-gene pairs exist. (TIF) [file pgen.1007071.s020.tif]
